# Supplementary material for: IRE1 RNase controls CD95-mediated cell death
Source: EMBO Rep. 2024 Feb 21;25(4):13. doi: 10.1038/s44319-024-00095-9 (PMC11014915; doi:10.1038/s44319-024-00095-9)
Supplement: Supplementary file 1 — Appendix [file 44319_2024_95_MOESM1_ESM.pdf]

Appendix for article "IRE1 RNase controls CD95-mediated cell death"

Appendix Figure S1 .... **2**
Appendix Figure S2 .... **3**
Appendix Figure S3 .... **4**
Appendix Figure S4 .... **5**
Appendix Figure S5 .... **6**
Appendix Table S1 .... **8**
Appendix Table S2 .... **9**

Appendix Figure S1

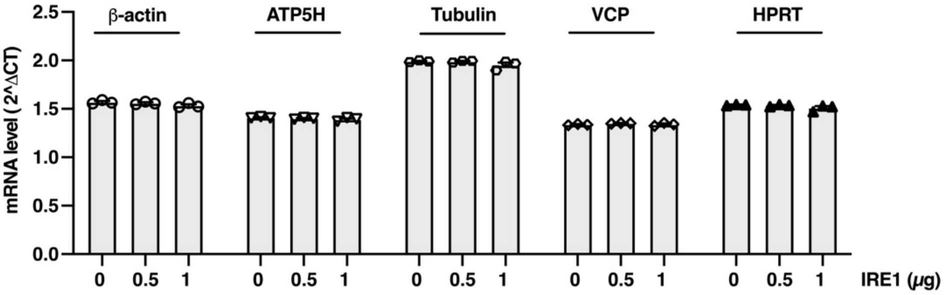

Appendix Figure S1- IRE1 does not cleave all RNAs *in vitro*. RNA (2 µg) extracted from U87 cells was incubated with the indicated amounts of recombinant IRE1 for 1 hour. The indicated mRNAs were then quantified by RT-qPCR and normalized to GAPDH. Mean ± SEM, n=3.

Appendix Figure S2

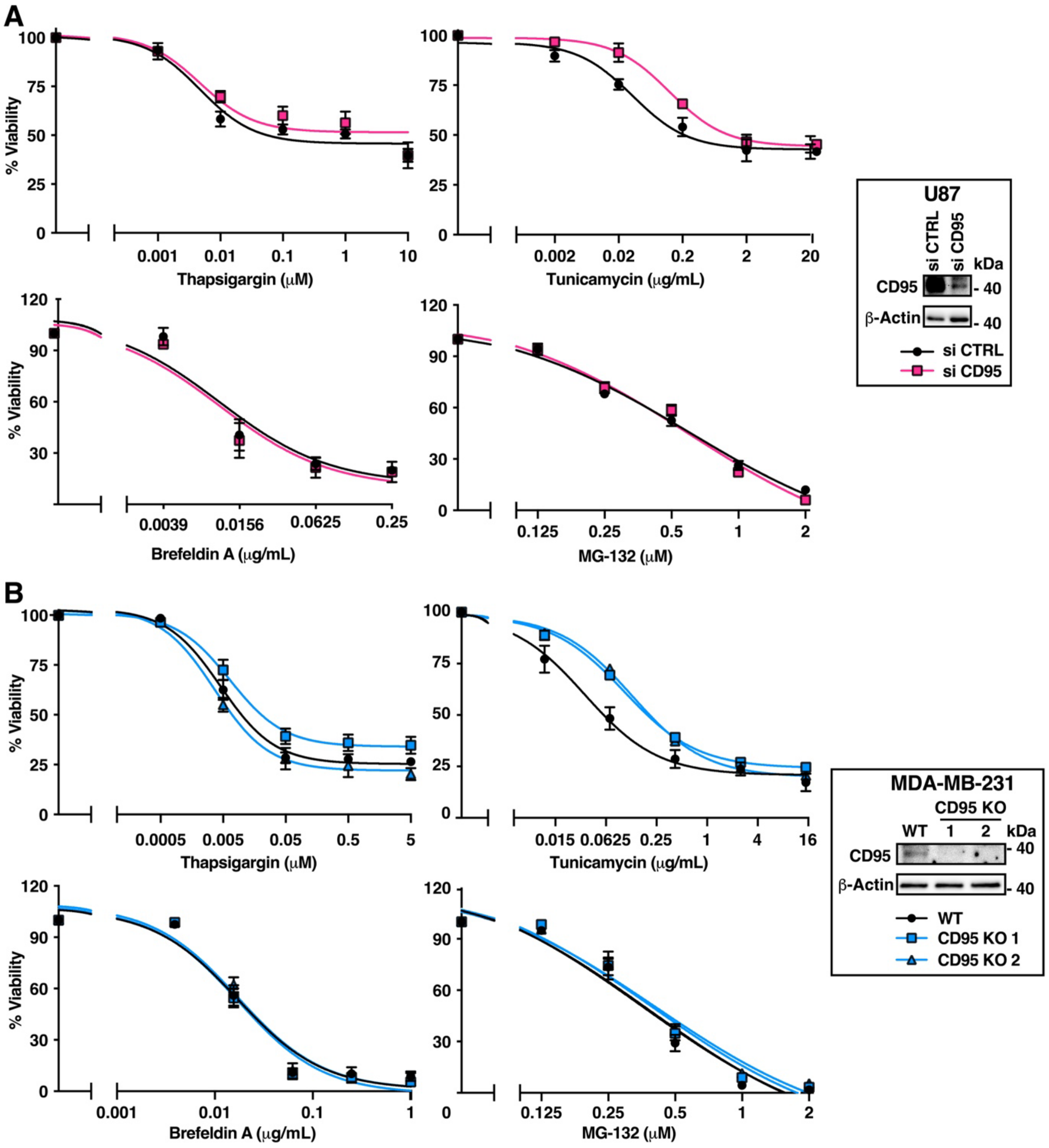

Appendix Figure S2- CD95 is not a general determinant of ER stress-induced cell death. A. U87 transfected with control (CTRL) or CD95-targeting siRNAs were treated with the indicated ER stress inducers for 48 hours. Viability was assessed using MTT assay. Mean ± SEM, n=3-4 independent experiments. **B.** MDA-MB-231 WT or CD95 KO clones were treated with the indicated ER stress inducers for 48 hours. Viability was assessed using MTT assay. Mean ± SEM, n=4-5 independent experiments. **A, B.** Insets: lysates were analysed using western blot using the indicated antibodies.

Appendix Figure S3

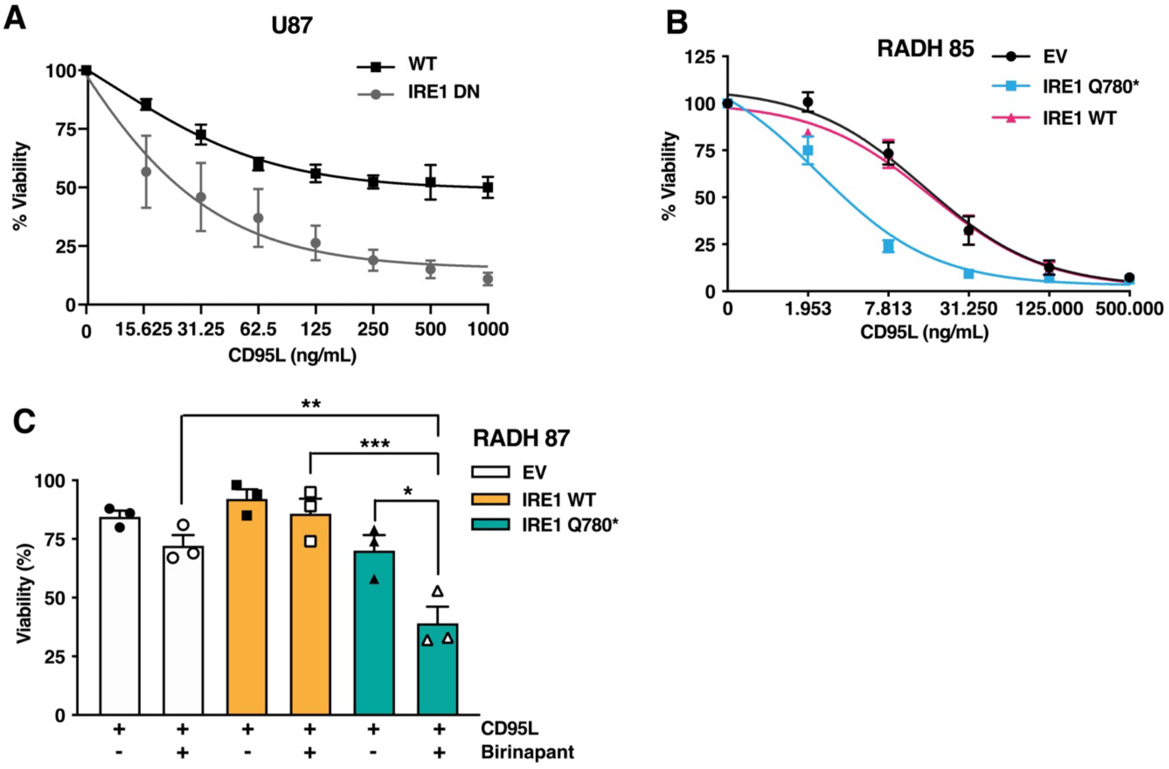

Appendix Figure S3- IRE1 RNase activity limits CD95L-induced loss of viability. A. U87 WT or expressing IRE1DN were treated with the indicated concentrations of CD95L for 24 hours. Viability was determined using MTT assay and normalized to untreated cell values. Mean ± SEM of 3 independent experiments. **B.** RADH85 control (EV), stably expressing IRE1Q780* or IRE1WT were treated with the indicated concentrations of CD95L for 24 hours. Viability was determined using MTT assay and normalised to untreated cells values. Mean ± SEM of 3 independent experiments. **C.** RADH87 control (EV), stably expressing IRE1Q780* or IRE1WT were pre-treated with 100 nM (2X) Birinapant and further treated with 1 µg/mL CD95L for 24 hours. Viability was determined using MTT assay and normalised to untreated cells values. Mean ± SEM of 3 independent experiments. *p=0.0127, *** p=0.0004, ** p= 0.0081., one-way ANOVA with Tukey multiple comparison correction.

Appendix Figure S4

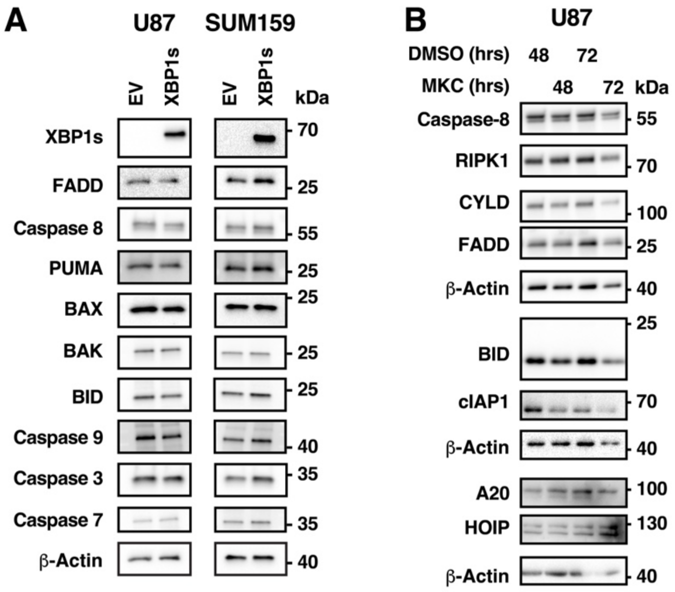

Appendix Figure S4- IRE1 RNase activity controls CD95 expression in GB and TNBC cell lines. A. U87 or SUM159 cells were transfected with a plasmid coding for FLAG-XBP1s (XBP1s) or an empty vector (EV). 48 hours later, cell lysates were analysed using western blot. One experiment representative of three independent ones is shown. The experiment presented in S4A (U87 cell panel) is the same as depicted in Figure 5B. The experiment presented in S4A (SUM 159 cell panel) is the same as depicted in Figure EV4B. **B.** U87 cells were treated with DMSO or MKC-8866 (30 μM) for the indicated times. Lysates were analysed using western blot. One experiment representative of three independent ones is shown. The experiment presented in S4B is the same as depicted in Figure 5C.

Appendix Figure S5

```
human      -----AATAGTTTTAGGATTTCA---------------------AAAAATTTGCAGAGAT      -1466
mouse      GGCTATCTAGTCTCAGGTTCTTGGGTAGCGTCAGGTTTATGTTCTATCTTGTGGAGTGGG      -1440
                  **** * *** * *                        *   * ** ** *

human      AATACAGAGAATGCCCATATACCATCCTCCTTATCCCACTTCTTTTTGTGTCTATTAGAT      -1406
mouse      GCTAAAGTCAAATCCAGTATTGATGG--GTTGCTCCCACAAAC-TTTGTGC-CACTATAT      -1386
             ** **  **  **  ***          *  ******     ******   * ** **

human      GCTCAGAGTGTGTGCACAAGGCTGGCACGCCCAGGGTCTTCCTCATGGCACTAACAGTCT      -1346
mouse      -------------GC---------------------ACTAGCTCATCTCTCTAGCAGGAC      -1360
                        **                      **  *****  * *** ***

human      ACTGAAAGGTGGAACAGAGACAAGCCTATCAACACCTACAAGACTGGTGGTAAGTGCAGT      -1286
mouse      A-------------------------------------------------------CCAT      -1355
           *                                                       *  *

human      GACAGATGCAAAACACAGGGTGATGGAAAGCCCTCAGGAGGGTAACCTAAC--CTAGATT      -1228
mouse      CATAGATC------AAAGGCTTTGGGGTAGAGG---TGAGGTTTACCTTTCTCCTTTAGT      -1302
            * ****       * *** *   **  **       **** * ****  *  **  * *

human      TGAGGGCCCAAACAGGCTCCAGAAGAAAA--TGTCAACTGAGAGGAAGCCTGAAGGATGA      -1171
mouse      AGCAT-GCAGAATATCTTCCAGTACCAAAGATGTTGGCAT---GTAGGGATGAAGGCTCT      -1246
            *     *  ** *   ***** *  ***  ***   *     * * *  ****** *

human      ACAGTGGGCTAAGCAAAGGGTTATT------AA----TGTGTTATTAATGGGTT----GA      -1125
mouse      ATG------TAGGCACTTTCTTCTTGCTCTCAACTTCTCTATATTCAATGAGTTGTGTGG      -1192
           *        ** ***     ** **      **    * * *  * **** ***    *

human      ATCTAATTGGGAAGGG------------------AGAGAGGTTGCAG---------AGTG      -1092
mouse      GTCTCAGTTTGTGGGGAGCAATCTATAGTCTTGGCAACAGGCTGCATTGTTTGGGGACTC      -1132
            *** * *  *  ***                    * *** ****          * *

human      AGGTGCAGAGCTTGGTGG---ACGATGCC----------------------AAAGGAATA      -1058
mouse      CCATGCAACTCTTTTTGGCCAACGATTCAATTAGATGCAACCCCATCCAGGAAAGGAAAA      -1072
              ****   ***  ***   ***** *                       ******* *

human      CTGA--------------------------------------------------------      -1054
mouse      CTTCATTTGATGACAAGAGATGTTCACTTGTGGCTCTGTCTCCCCCCATTATTTGGCTAT      -1012
           **

human      ------------------------------------------------------------      -1054
mouse      AGATCACCTTCATGTATGCTTATACTTTAGGAAGACTCTGCTAGGTTTCCATACCTCTCA      -952


human      ---AACCTTTAGTGTGTCCAGTCTGGAACTGCATCCAAATTCAGGTTCAGTAATGATGTC      -997
mouse      AATGCCCTTTAATTTTAGCTGTCTCTCCCTGTATTCCCATTCATCGTCT-----------      -903
                ****** * *   * ****    *** ** *  *****   **

human      ATTATCCAAACATACCTTCTGTAAAATTCATGCTAAACTACCTAAGAGCTATCTACCGTT      -937
mouse      ------------------------------------------------------------      -903


human      CCAAAGCAATAGTGACTTTGAACAGTGTTCACCAGAGCACGAAAGAATTACAAGATTTTT      -877
mouse      -------------TCCTTTCCC-CTCCCCCACTTGATCTTCCCATCATAACTACCTATTT      -857
                          ****          ***  ** *     *  ** ** *  * ***

human      ---------TTTTAAAGAAAATTGGCC-------------AGGA-AATAATGAGTAACGA      -840
mouse      TATTCCTCTTCCTTAATAAGATCTTTCTGTCCCCTCCCCTAGTCCCTTACTCTATATCTA      -797
                    *  * ** ** **    *             **     ** *   ** * *

human      AGGACAGGAAGTAATTGTGAATGTT-------------TAATATAGCTGGGGCTATGCGA      -793
mouse      ACCCCC----GTGGTTCTAAAGATTCTGGCTTGGTCCCTGAGTTAAC---TGCTGTGTTA      -744
           *   *     **  ** * **  **             * *  ** *    *** **  *

human      TTTGGCTTAAGTTGTTAGCTTTGTTTTCCTCTTGAGAAATAAAAACTAAGGGGCCCT-CC      -733
mouse      TTTGTCATAGTCATTTACCTTCATTCTCCATTTGGGAAACGAAGC--CAGGGACATTCCC      -686
           **** * **     *** ***  ** ***  *** ****  **     **** *  * **

human      CTTTTCAGAGCCCTATGGCGCAACATCTGTACTTTTTCATATGGTTAACTGTCCATTCCA      -673
mouse      CTCATCAGGATCCCAAAGCCTATTATCTCTACCTCTGC-TGTGG-TGATTGTCACTTCAT      -628
           **  ****   ** *  **  *  **** *** * * * * *** * * ****  ***

human      GAAACGTCTGTGAGC------------------------------------------CTC      -655
mouse      GTATCATATATGAGCTCTGTGGAGGGACCTCCCTCATCAGAATCCTAGGGTGTGTACCTC      -568
           * * * * * *****                                          ***
```

```
human      TCATGTTGCAGCCACAACATGGACAGCCCAGTCAAATGCCCC----------GCAAGTCT     -605
mouse      CTCTGTGGTAATTA-------------CCATTCAGAGGCCCCAGAAGCCCCAGCAGACCT     -521
              *** * *   *             *** *** * *****          ***   **

human      TTCTCTGAGT-------------------------------GACTCCAGCAATTAGCCAA     -576
mouse      GTCACTGACAAAATATTGAAAGTGAAGGCAAAATGGTCTTTGTCTCCAGCAATCAGCCAA     -461
            ** ****                                 * ********** ******

human      GGCTCCTGTACCCAGGCAGGACCTCTGCGCTCTGAGCTCCATTCTCCTTCAAGACCT---     -519
mouse      AGT----------------------------------------CCAATTCAATACAGAGT     -441
            *                                         *   ***** **

human      CCCCAA--CTTCCCAGGTTGAACTACAGCAGAAGCCTTTAGAAAGGGCAGGAGGCCGGCT     -461
mouse      TCAGAATTCCTTCCAGGGTGAG-------------------------CAGCAGGCAGAAA     -406
            *  **  * * ***** ***                          *** **** *

human      CTCGAGGTCCTCACCTGAAGTGAGCATGCCAGCCACTGCAGGAACGCCCCGGGACAGGAA     -401
mouse      --AAAAAATCTCACTTGACCTGAGGGTGT----GACTGTGGAAGCGCCCAGGATGGGGAA     -352
               *    ***** ***  ****  **      ****  * * ***** **    ****

human      TGCCCATTTGTGCAACGAACCCTGACTCCTTCCTCACCCTGACTTCTCCCCCTCCCTACC     -341
mouse      TGCCCATTTATGCAATCAAGCCCTGCTTGGGCCCCCTCCTCCCCCGCCCCCCACCCCCAC     -292
           ********* *****  ** **   **    ** *  ***  *    ***** ***   *

human      CGCGCGCAGGCCAAG------TTGCTGAATCAATGGAGCCCTCCCCAACCCGGGCGTTCC     -287
mouse      CCCGCTCAGGCCGCCTGTGCAGTGGTGAGTCAGTGG-------------------GTTTC     -251
           * *** ******          ** *** *** ***                   *** *

human      CCAGCGAGGCTTCCTTCCCATCCTCCTGACCACCGGGGCTTTTCGTGAGCTCGTCTCTGA     -227
mouse      CCCGGGAGACCAGCAGAACTTTCTCGCAACCCTTGGGGCTTTACG-AAGATTGTGTTTGG     -192
           ** * *** *   *    * * ***   ***   ******** **  ** * ** * **

human      TCTCGCGC------AAGAGTGACACACAGGTGTTCAAAGACGCTTCTGGGGAGT------     -179
mouse      TCCCTCAAAAGAAAAAAAGTGACATACACGTGTTCAAAGCGATTTCTGGGAAGACCTGAA     -132
           ** * *        ** ******* *** **********    ******* **

human      ---GAGGGAAGCGGTTTACGAGTGACTTGGCTGGAGCCTCAGGGGCGGGCACTGGCACGG     -122
mouse      TAGGAGCGAAGCGGTTTGTGCGTGCC-------------AGGGGGCGGGCCTAGGCAAAG     -85
              *** ********** *  *** *               *********   ****  *

human      AACACACCCTGAGGCCAGCCCTGGCTGCCCAGGCGGAGCTGCCTCTTCTCCCGCGGGTTG     -62
mouse      TACATACCCACAGGCAGTCTAGAGCTG-----------------------------GTGG     -54
            *** ****  ****   *    ****                             ** *

human      GTGGACCCGCTCAGT----ACGGAGTTGGGGAAGCTCTTTCACTTCGGAGGATTGCTCAA     -6
mouse      GTGGGGCCGCAGGCTGCCCACACAGGCC-GCCCGCTGTTTTCCC--------TTGCT--G     -6
           ****  ****    *    **  **    *   *** ***  *         *****

human      CAACCATGCTGGGCATCTGGACCCTC 21
mouse      CAGACATGCTGTGGATCTGGGCTGTC 21
           **  ******* * ****** *  **
```

Appendix Figure S5- Putative XBP1s-binding sites on human and mouse promoters of *FAS* gene. Promoter regions (up to -1500 bp from the start codon in green) of human and mouse *FAS* genes were aligned and tested for XBP1s binding sites using Clustal Omega and TBbind tools. Start codons and predicted XBP1s sites described in Appendix Table 1 were underlined in yellow (human) or blue (mouse).

Appendix Table S1

	Putative site	Position (from the start codon)	TFbind score
Human			
site1	CGGTTTACGAGTGACTT	-170	0.750641
site2	TTATCCCACTTCTTTTT	-1436	0.744017
Mouse			
site1	ACATACACGTGTTCAAA	-171	0.817094
site2	GACATACACGTGTTCAA	-172	0.805342
site3	ATGTTCACTTGTGGCTC	-1053	0.745726
site4	GGGTAGAGGTGAGGTTT	-1335	0.745940

Appendix Table S1- Putative XBP1s-binding sites on human and mouse promoters of *FAS* gene. Promoter regions (-1500 bp from the start codon in green) of human and mouse *FAS* genes were tested for XBP1s binding sites using the TFBind tool.

Appendix Table S2- GB transcriptome samples ID used in this study

https://pubmed.ncbi.nlm.nih.gov/24120142/

STUDY_ID	SAMPLE_ID
gbm_tcga_pub2013	TCGA-02-0047-01
gbm_tcga_pub2013	TCGA-02-0055-01
gbm_tcga_pub2013	TCGA-06-0125-01
gbm_tcga_pub2013	TCGA-06-0130-01
gbm_tcga_pub2013	TCGA-06-0132-01
gbm_tcga_pub2013	TCGA-06-0157-01
gbm_tcga_pub2013	TCGA-06-0178-01
gbm_tcga_pub2013	TCGA-06-0190-01
gbm_tcga_pub2013	TCGA-06-0210-01
gbm_tcga_pub2013	TCGA-06-0219-01
gbm_tcga_pub2013	TCGA-06-0686-01
gbm_tcga_pub2013	TCGA-06-0743-01
gbm_tcga_pub2013	TCGA-06-0744-01
gbm_tcga_pub2013	TCGA-06-0878-01
gbm_tcga_pub2013	TCGA-06-2562-01
gbm_tcga_pub2013	TCGA-06-2563-01
gbm_tcga_pub2013	TCGA-06-5411-01
gbm_tcga_pub2013	TCGA-06-5412-01
gbm_tcga_pub2013	TCGA-06-5415-01
gbm_tcga_pub2013	TCGA-12-1597-01
gbm_tcga_pub2013	TCGA-12-3650-01
gbm_tcga_pub2013	TCGA-14-0781-01
gbm_tcga_pub2013	TCGA-14-0817-01
gbm_tcga_pub2013	TCGA-14-1034-01
gbm_tcga_pub2013	TCGA-15-0742-01
gbm_tcga_pub2013	TCGA-15-1444-01
gbm_tcga_pub2013	TCGA-19-1390-01
gbm_tcga_pub2013	TCGA-19-2619-01
gbm_tcga_pub2013	TCGA-19-2624-01
gbm_tcga_pub2013	TCGA-19-2629-01
gbm_tcga_pub2013	TCGA-26-1442-01
gbm_tcga_pub2013	TCGA-26-5135-01
gbm_tcga_pub2013	TCGA-27-1830-01
gbm_tcga_pub2013	TCGA-27-1831-01
gbm_tcga_pub2013	TCGA-27-1837-01
gbm_tcga_pub2013	TCGA-27-2521-01
gbm_tcga_pub2013	TCGA-28-2499-01
gbm_tcga_pub2013	TCGA-28-5209-01
gbm_tcga_pub2013	TCGA-28-5215-01
gbm_tcga_pub2013	TCGA-28-5216-01
gbm_tcga_pub2013	TCGA-32-1980-01
gbm_tcga_pub2013	TCGA-32-2616-01
gbm_tcga_pub2013	TCGA-41-2571-01
gbm_tcga_pub2013	TCGA-41-2572-01
gbm_tcga_pub2013	TCGA-41-5651-01
