# Supplementary figures and images for: IRE1 RNase controls CD95-mediated cell death
Source: EMBO Rep. 2024 Feb 21;25(4):13. doi: 10.1038/s44319-024-00095-9 (PMC11014915; doi:10.1038/s44319-024-00095-9)

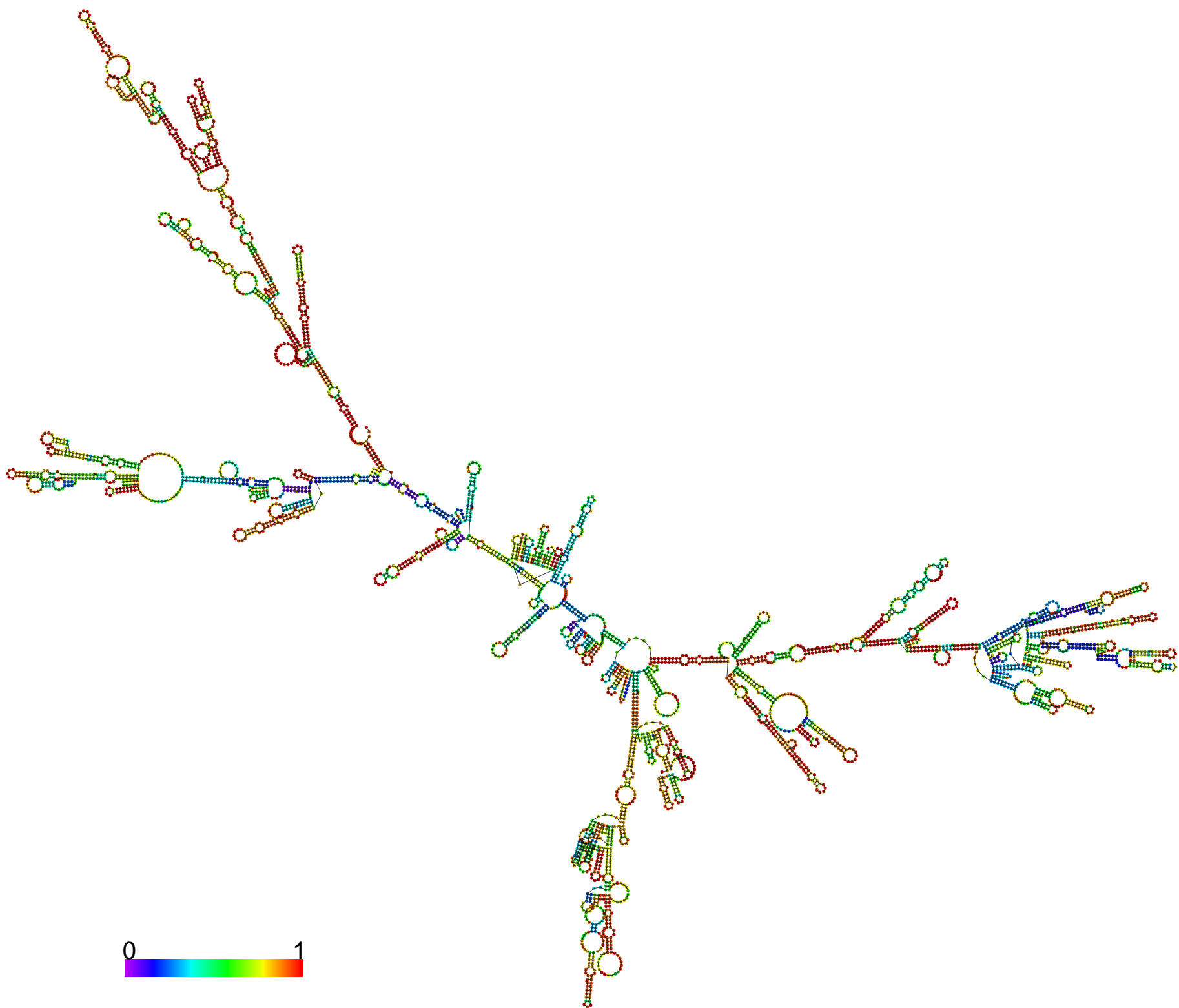

Supplement: Supplementary file 3 — Source Data Fig. 2 [file 44319_2024_95_MOESM3_ESM.zip › Source data Figure 2/Source dataFigure 2B.pdf]

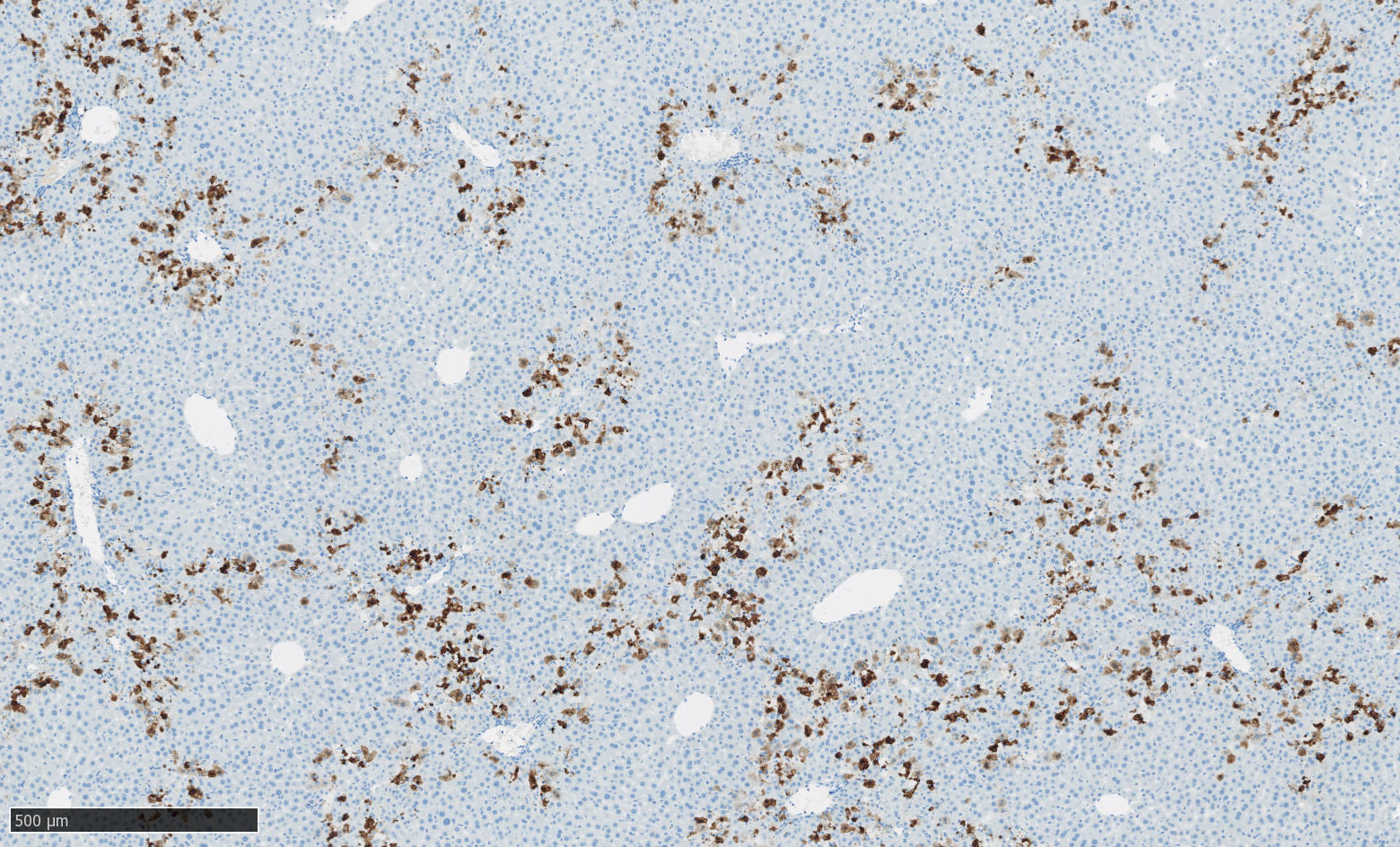

Supplement: Supplementary file 5 — Source Data Fig. 4 [file 44319_2024_95_MOESM5_ESM.zip › Source data Figure 4/Figure 4BC/886_CC3.jpg]

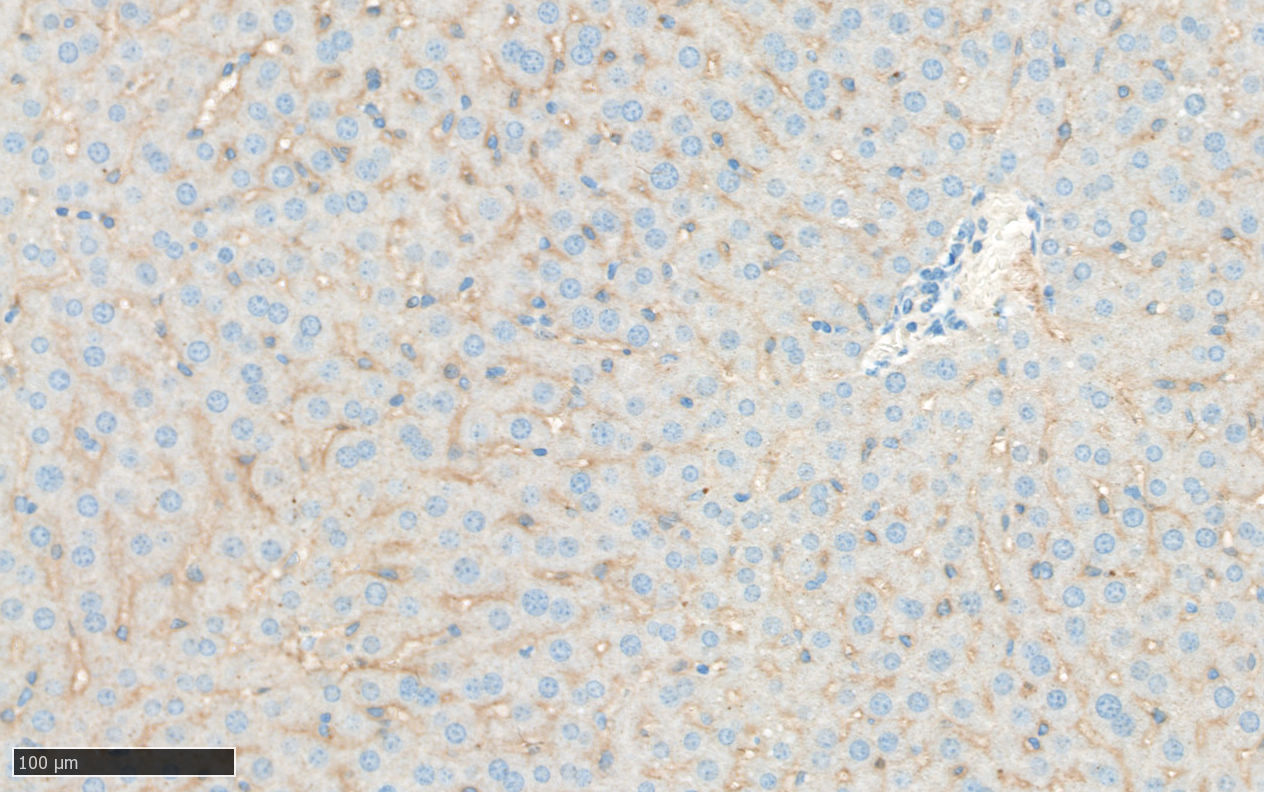

Supplement: Supplementary file 5 — Source Data Fig. 4 [file 44319_2024_95_MOESM5_ESM.zip › Source data Figure 4/Figure 4BC/791_represCD95x20.jpg]

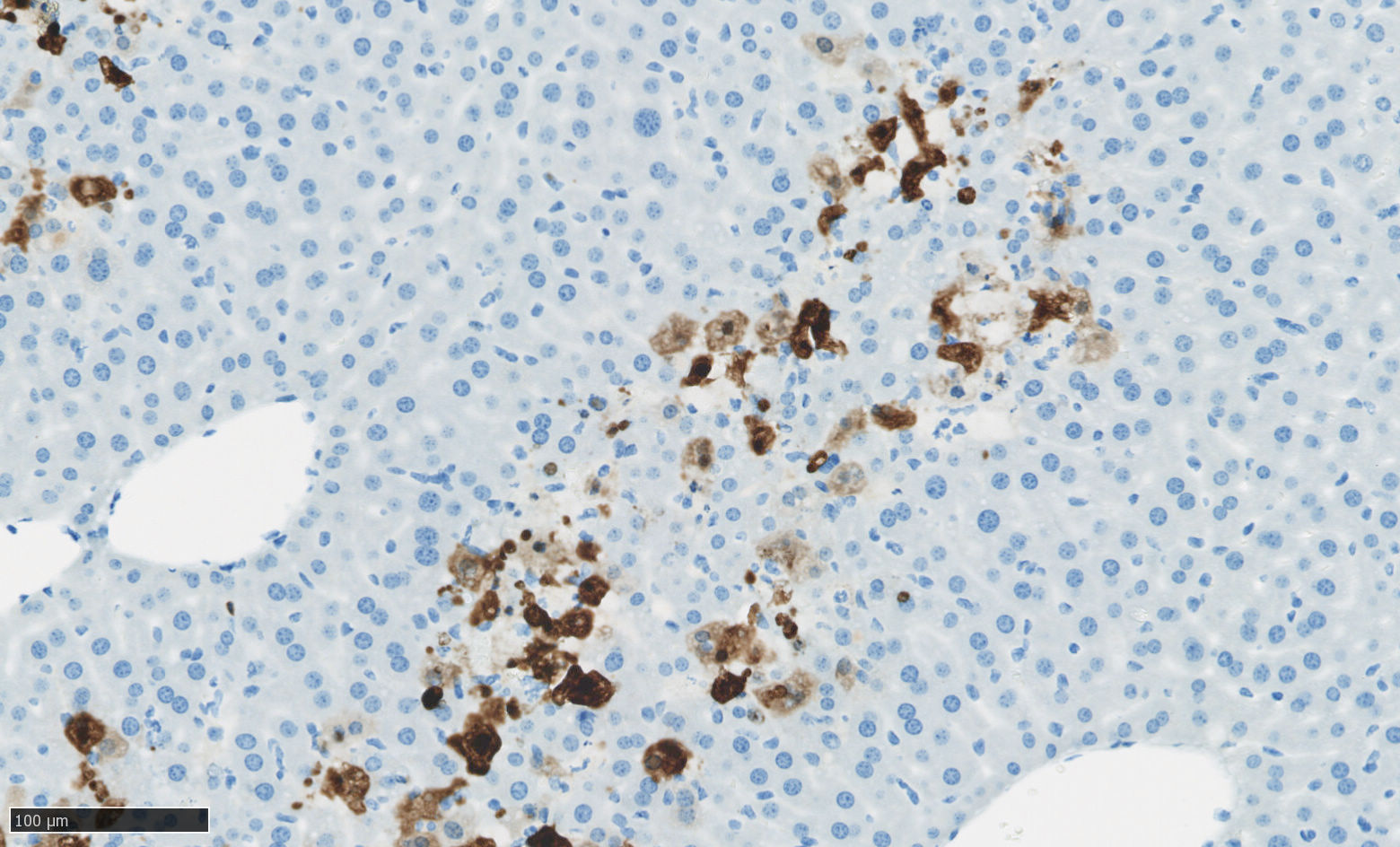

Supplement: Supplementary file 5 — Source Data Fig. 4 [file 44319_2024_95_MOESM5_ESM.zip › Source data Figure 4/Figure 4BC/886_CC3_zoom.jpg]

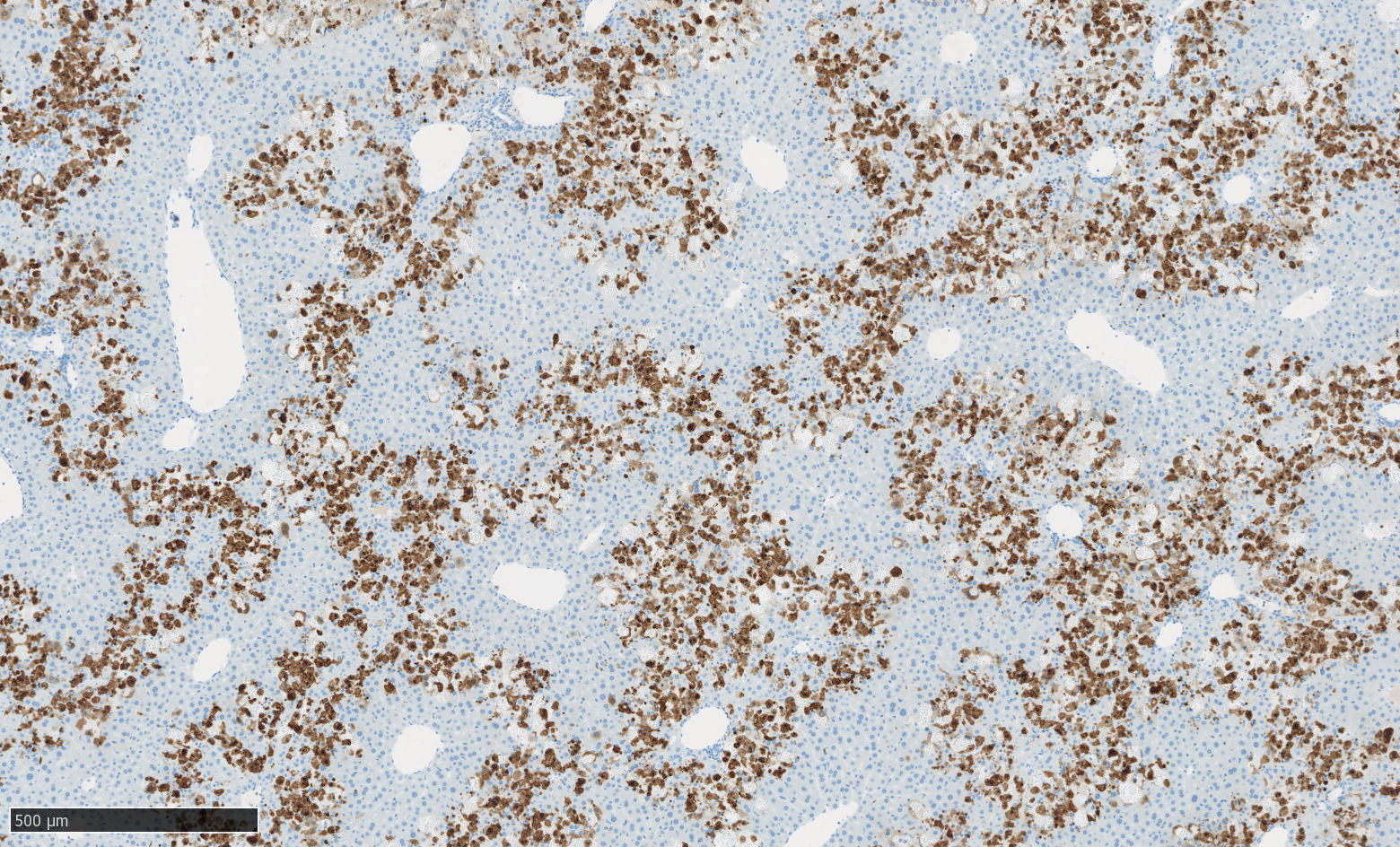

Supplement: Supplementary file 5 — Source Data Fig. 4 [file 44319_2024_95_MOESM5_ESM.zip › Source data Figure 4/Figure 4BC/869_CC3.jpg]

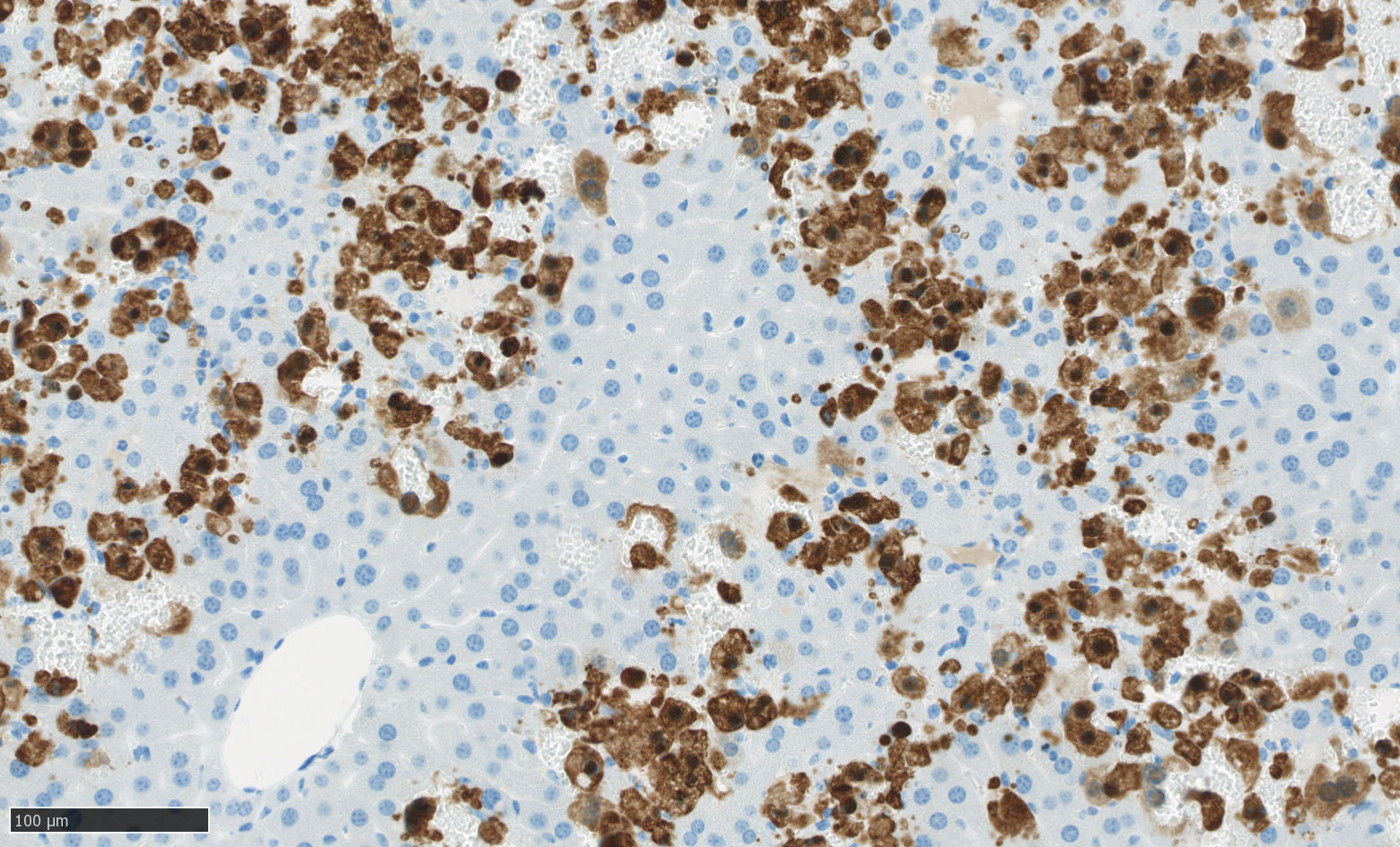

Supplement: Supplementary file 5 — Source Data Fig. 4 [file 44319_2024_95_MOESM5_ESM.zip › Source data Figure 4/Figure 4BC/869_CC3_zoom.jpg]

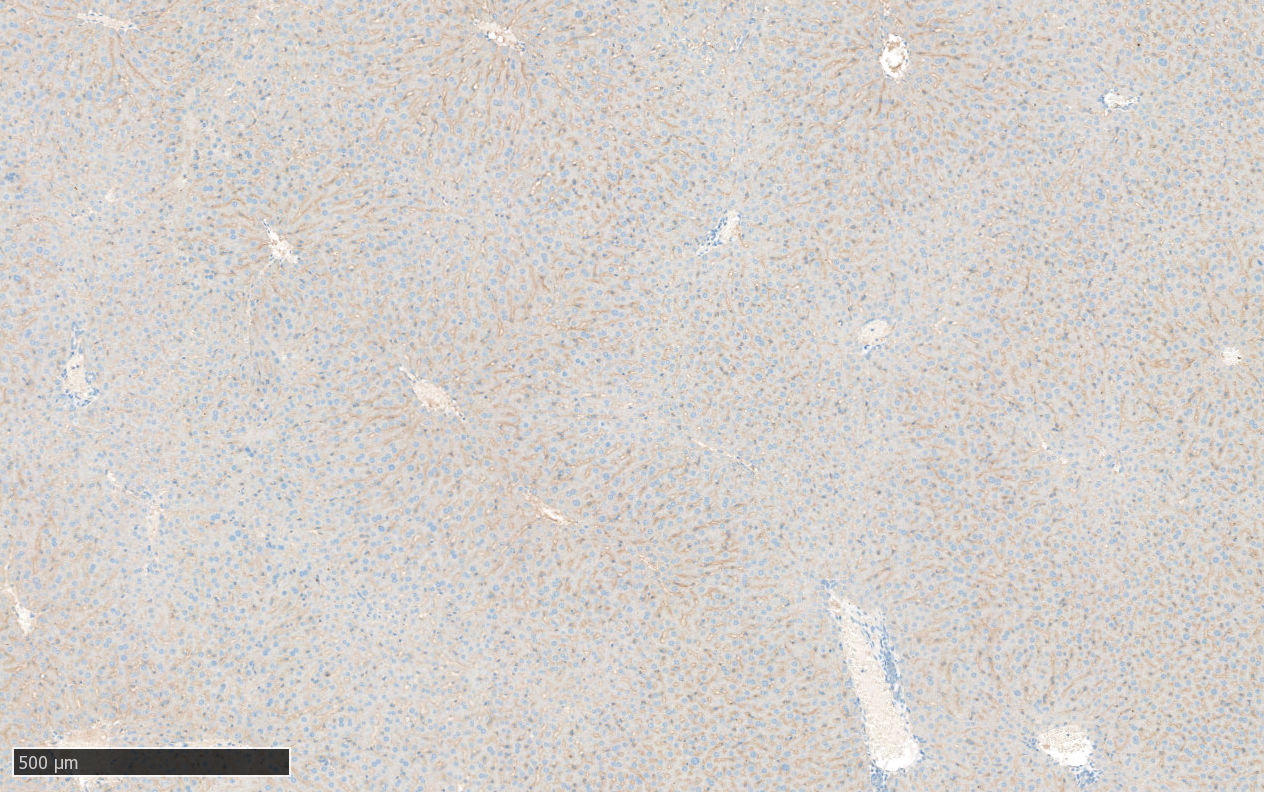

Supplement: Supplementary file 5 — Source Data Fig. 4 [file 44319_2024_95_MOESM5_ESM.zip › Source data Figure 4/Figure 4BC/791_represx5.jpg]

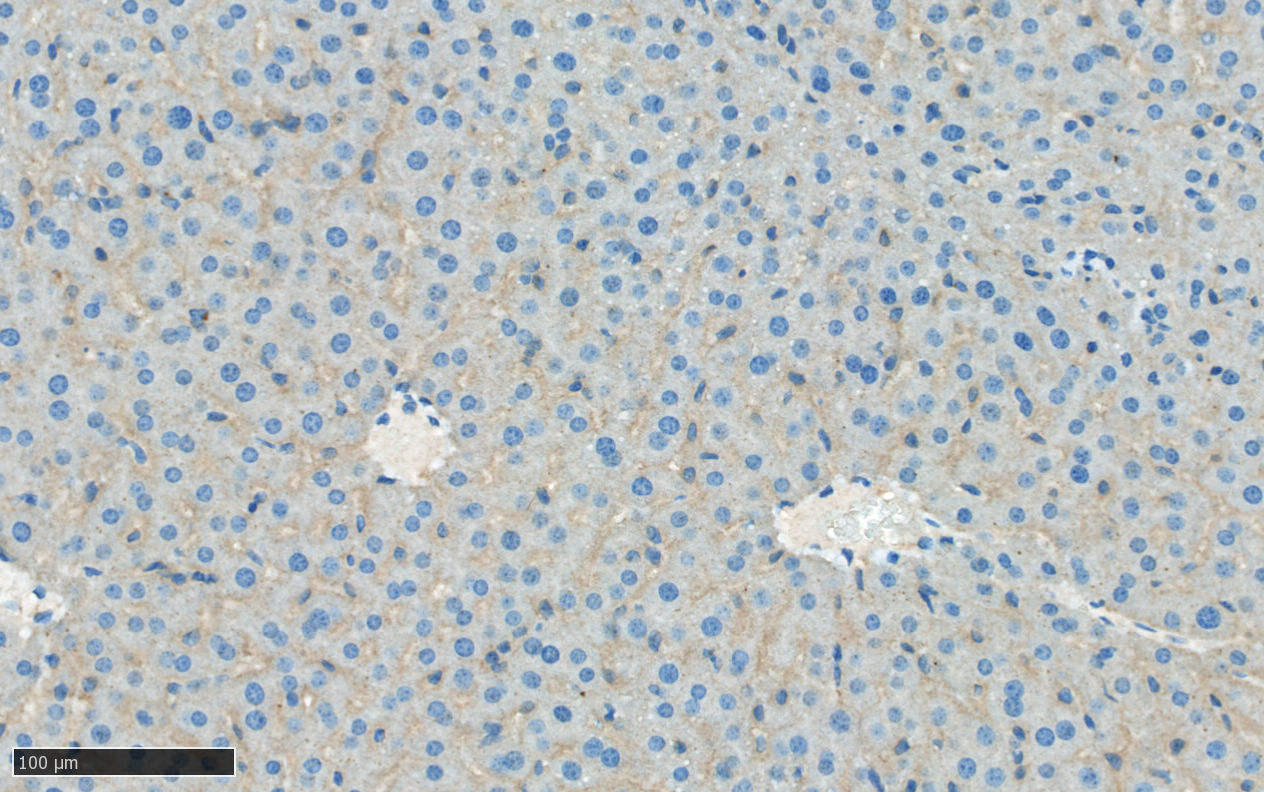

Supplement: Supplementary file 5 — Source Data Fig. 4 [file 44319_2024_95_MOESM5_ESM.zip › Source data Figure 4/Figure 4BC/823_represx20.jpg]

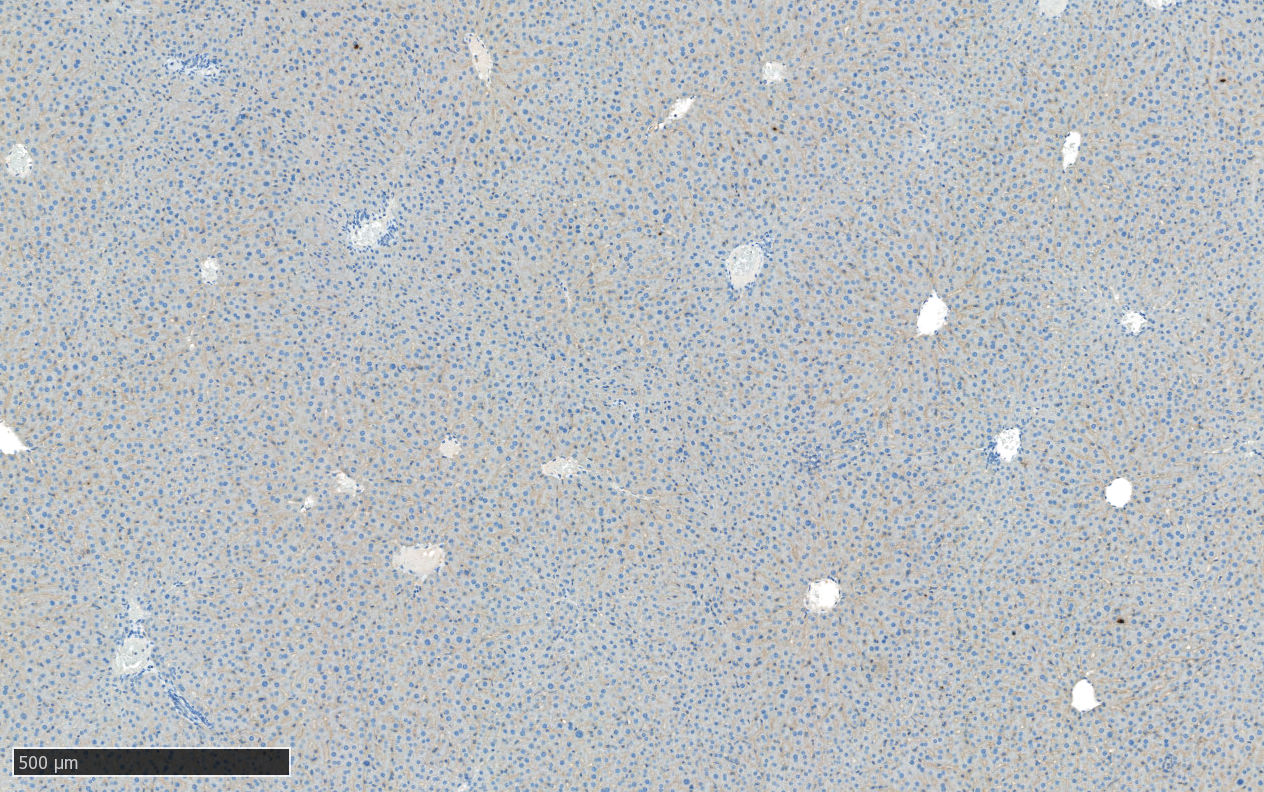

Supplement: Supplementary file 5 — Source Data Fig. 4 [file 44319_2024_95_MOESM5_ESM.zip › Source data Figure 4/Figure 4BC/823_repres_CD95x5.jpg]

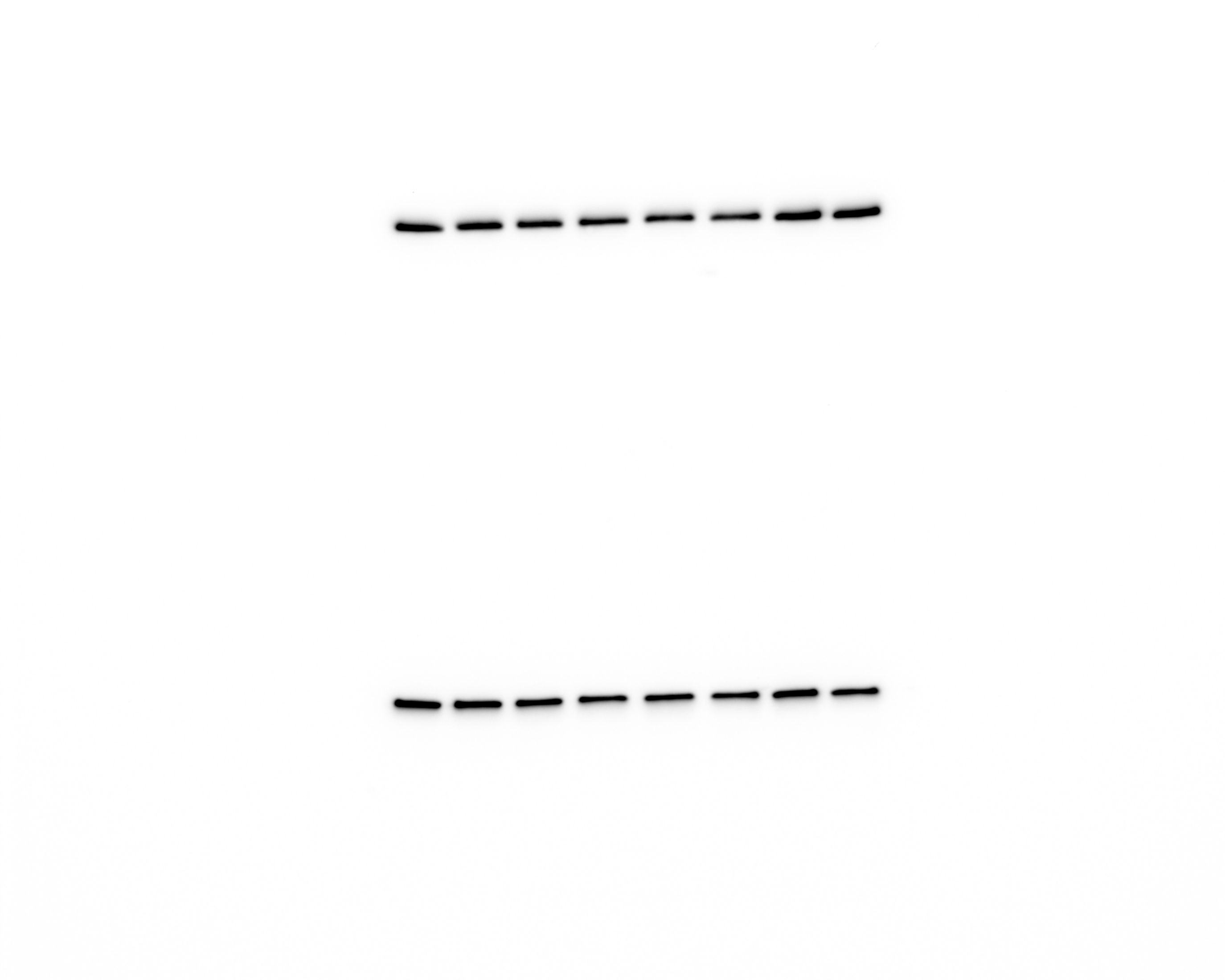

Supplement: Supplementary file 7 — Appendix and EV Figures Source Data [file 44319_2024_95_MOESM7_ESM.zip › Figure_EV4_SD/EV4A source data/EV4A individual files/actin-1.tif]

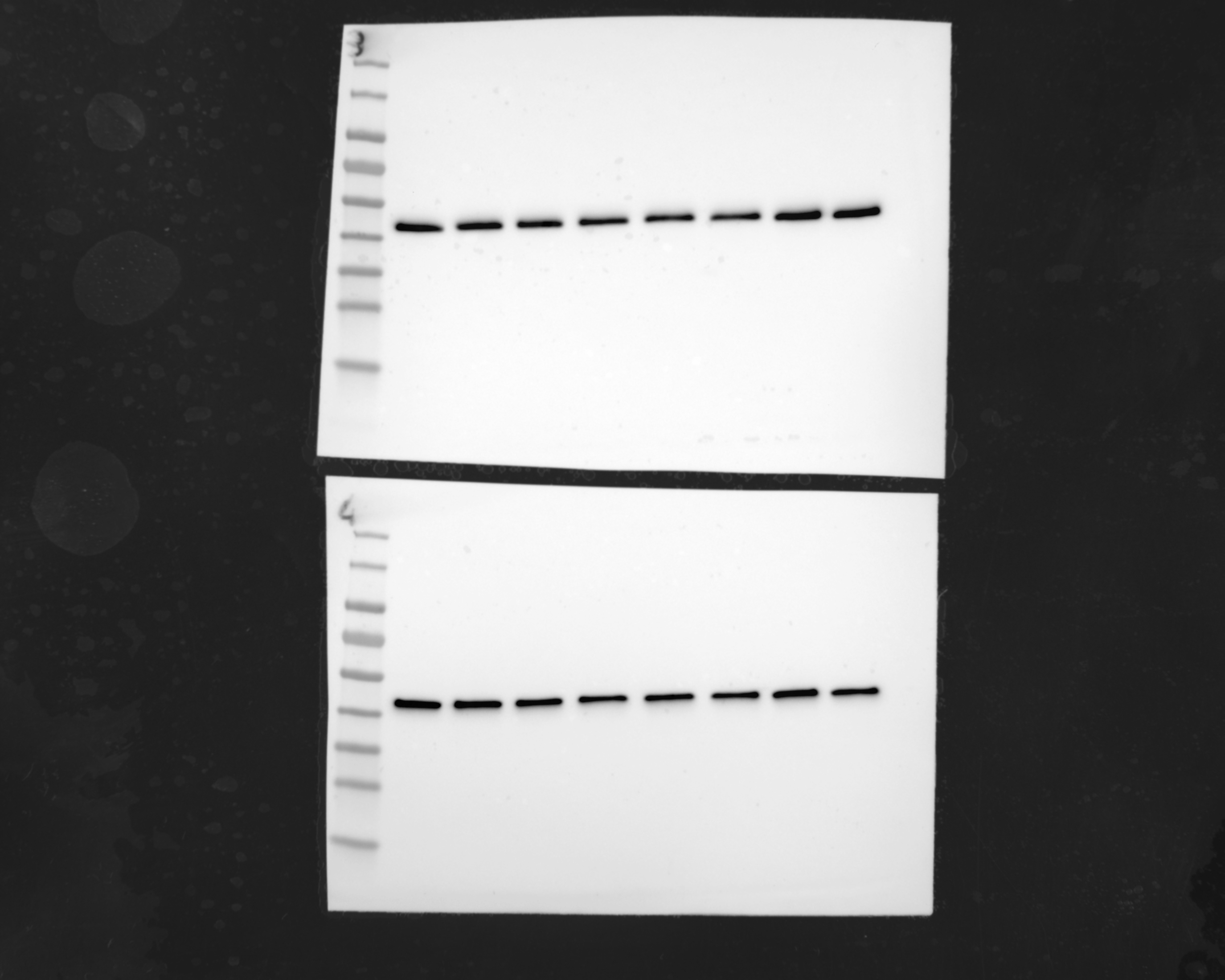

Supplement: Supplementary file 7 — Appendix and EV Figures Source Data [file 44319_2024_95_MOESM7_ESM.zip › Figure_EV4_SD/EV4A source data/EV4A individual files/actin-mark.tif]

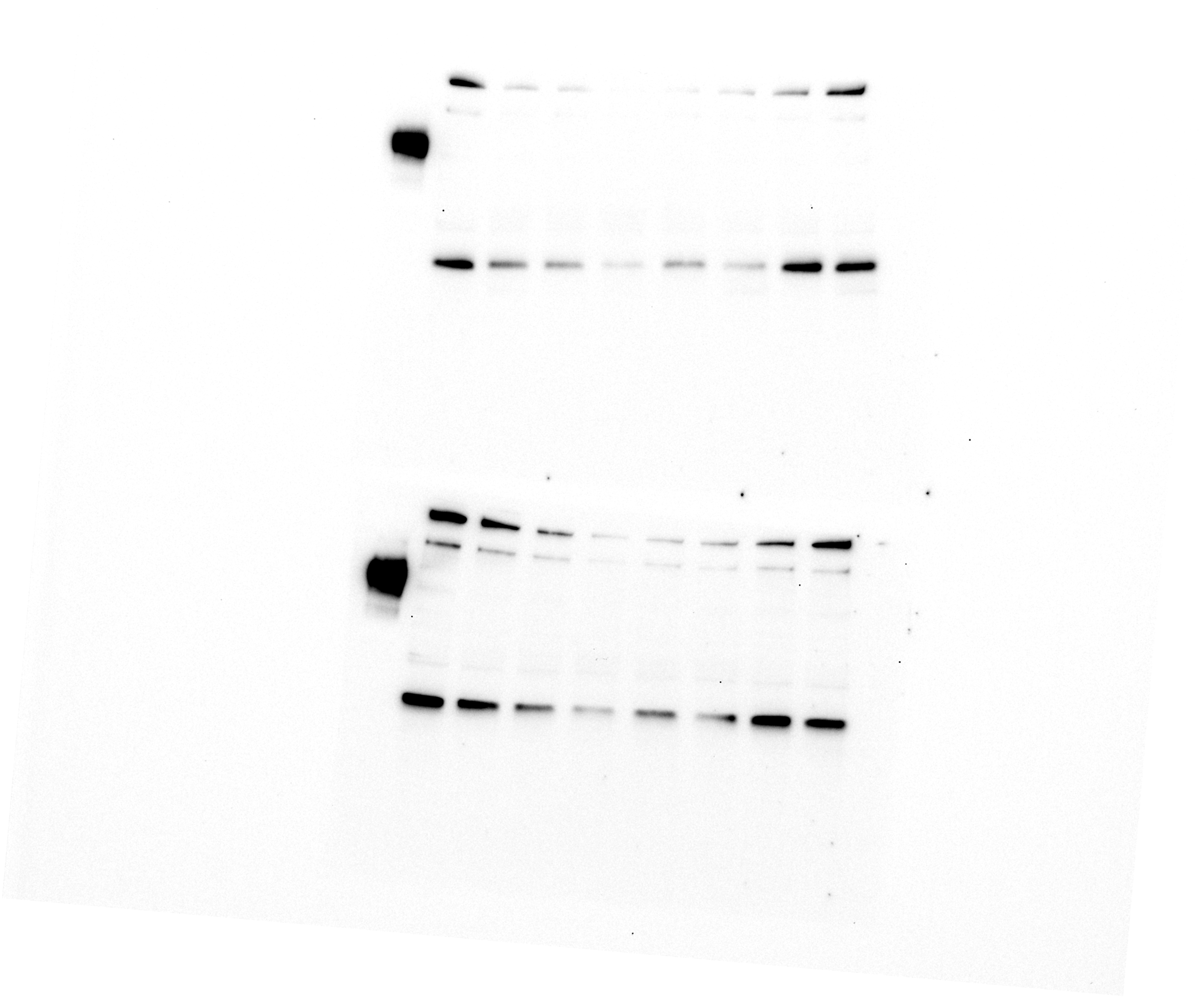

Supplement: Supplementary file 7 — Appendix and EV Figures Source Data [file 44319_2024_95_MOESM7_ESM.zip › Figure_EV4_SD/EV4A source data/EV4A individual files/CD95-3rot.tif]

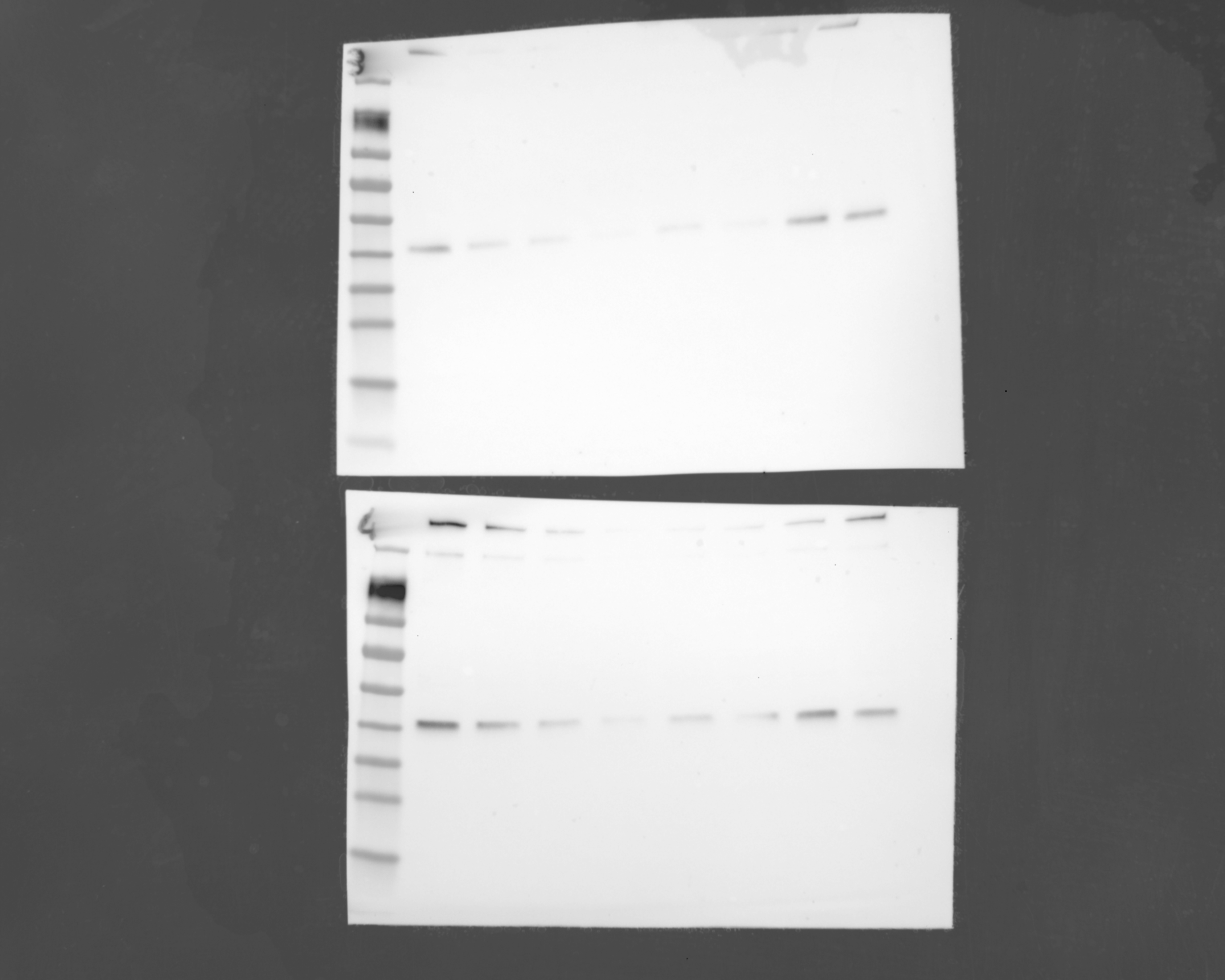

Supplement: Supplementary file 7 — Appendix and EV Figures Source Data [file 44319_2024_95_MOESM7_ESM.zip › Figure_EV4_SD/EV4A source data/EV4A individual files/CD95-mark.tif]

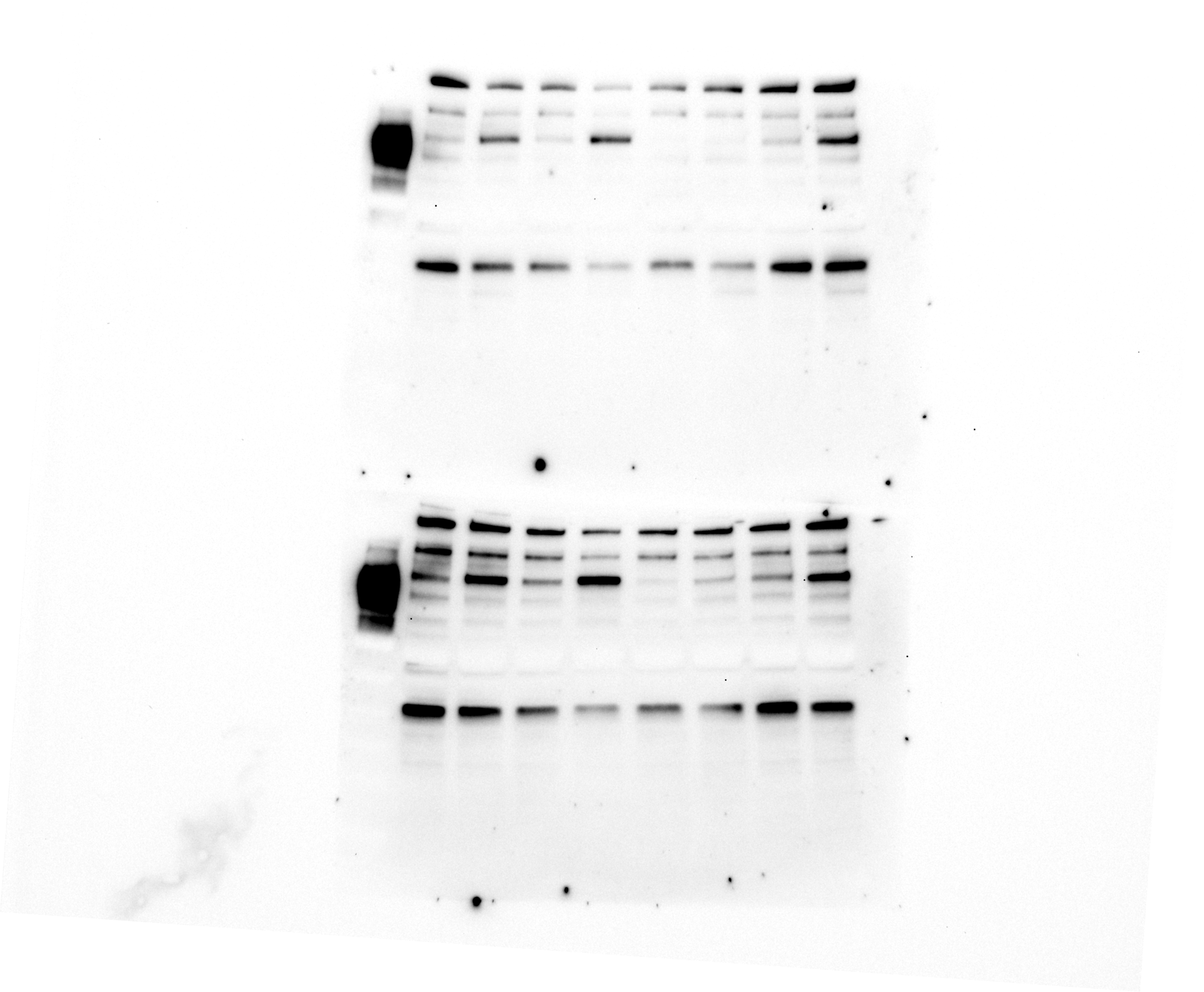

Supplement: Supplementary file 7 — Appendix and EV Figures Source Data [file 44319_2024_95_MOESM7_ESM.zip › Figure_EV4_SD/EV4A source data/EV4A individual files/ire1-1rot.tif]

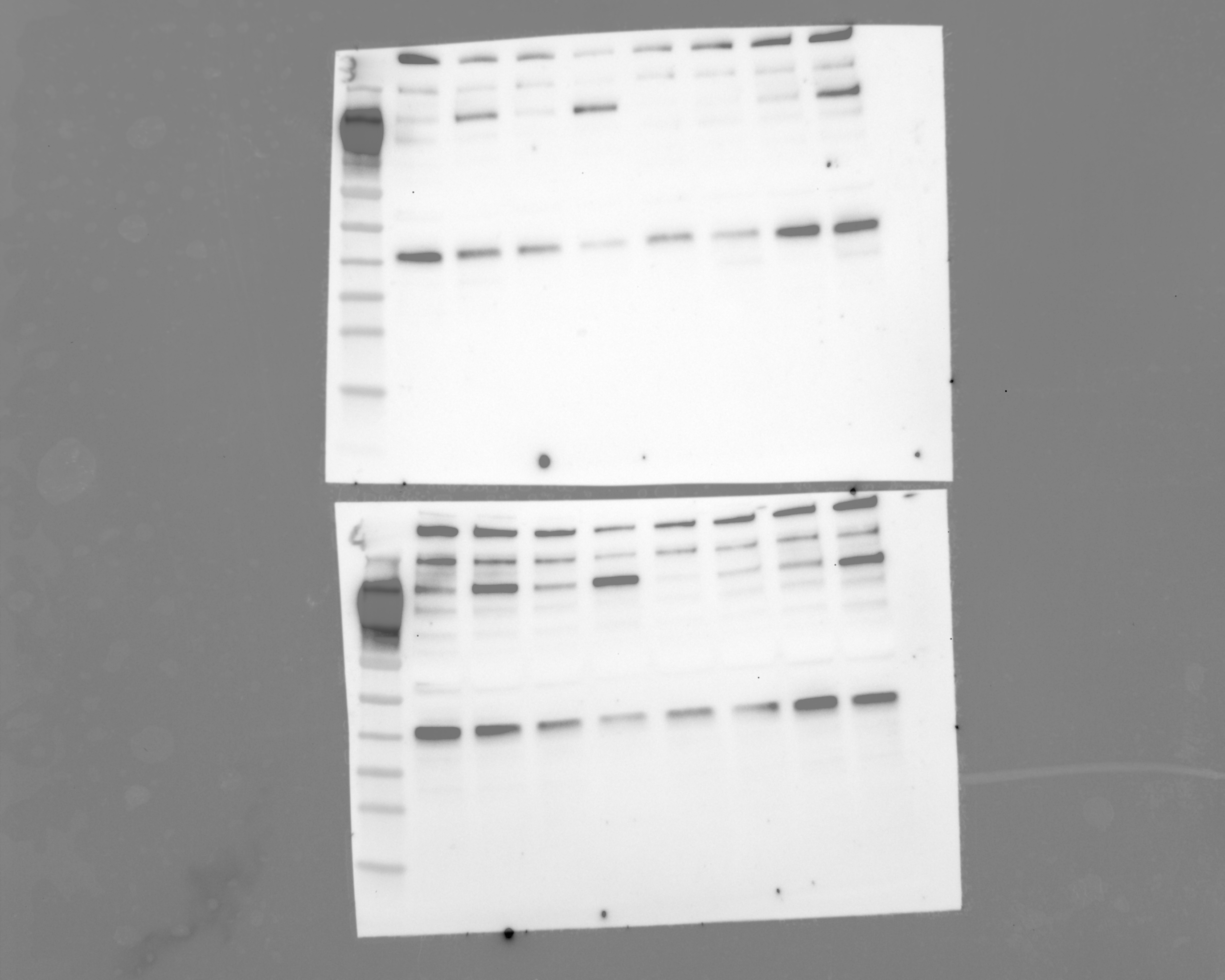

Supplement: Supplementary file 7 — Appendix and EV Figures Source Data [file 44319_2024_95_MOESM7_ESM.zip › Figure_EV4_SD/EV4A source data/EV4A individual files/ire1-mark.tif]

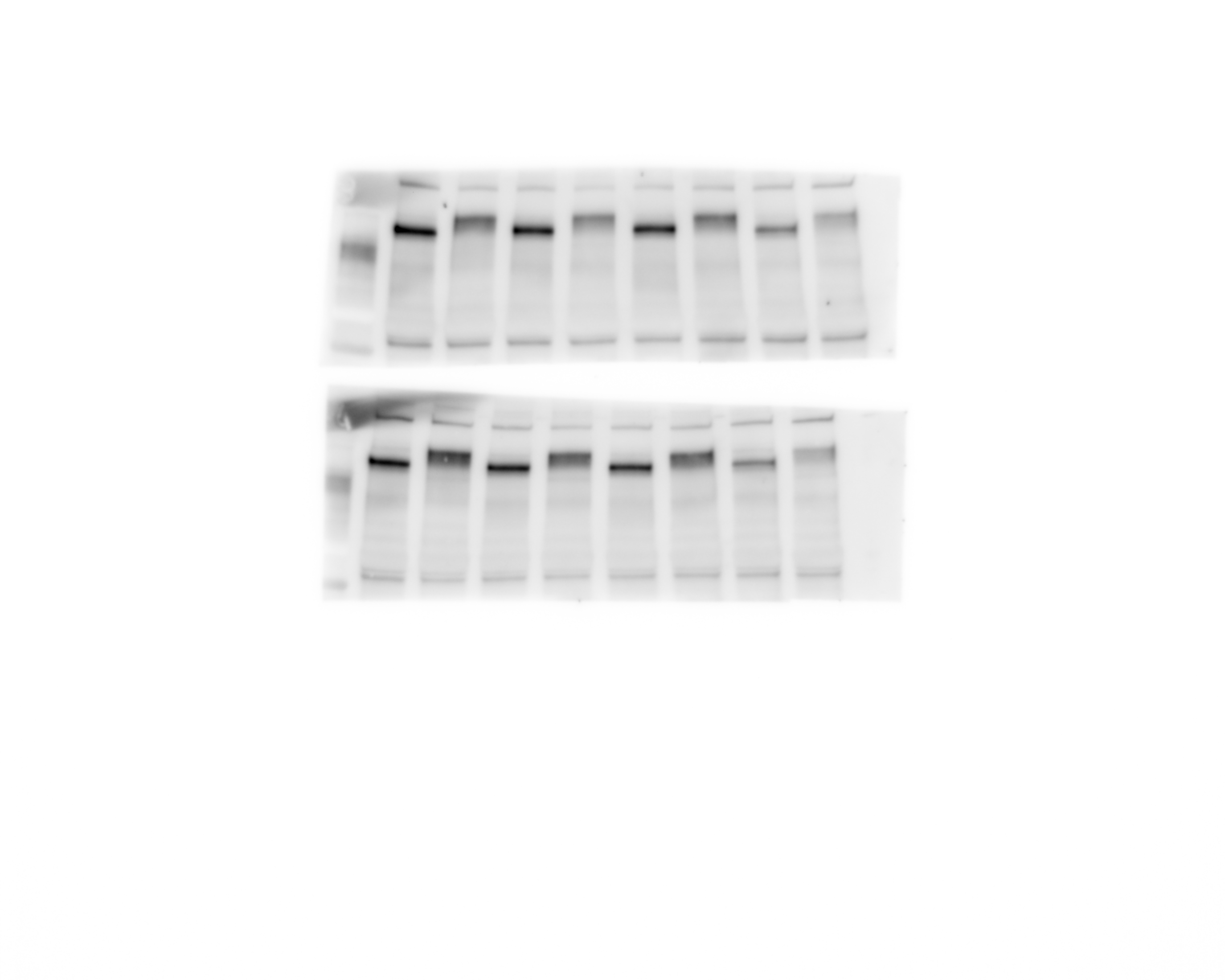

Supplement: Supplementary file 7 — Appendix and EV Figures Source Data [file 44319_2024_95_MOESM7_ESM.zip › Figure_EV4_SD/EV4A source data/EV4A individual files/perk-1.tif]

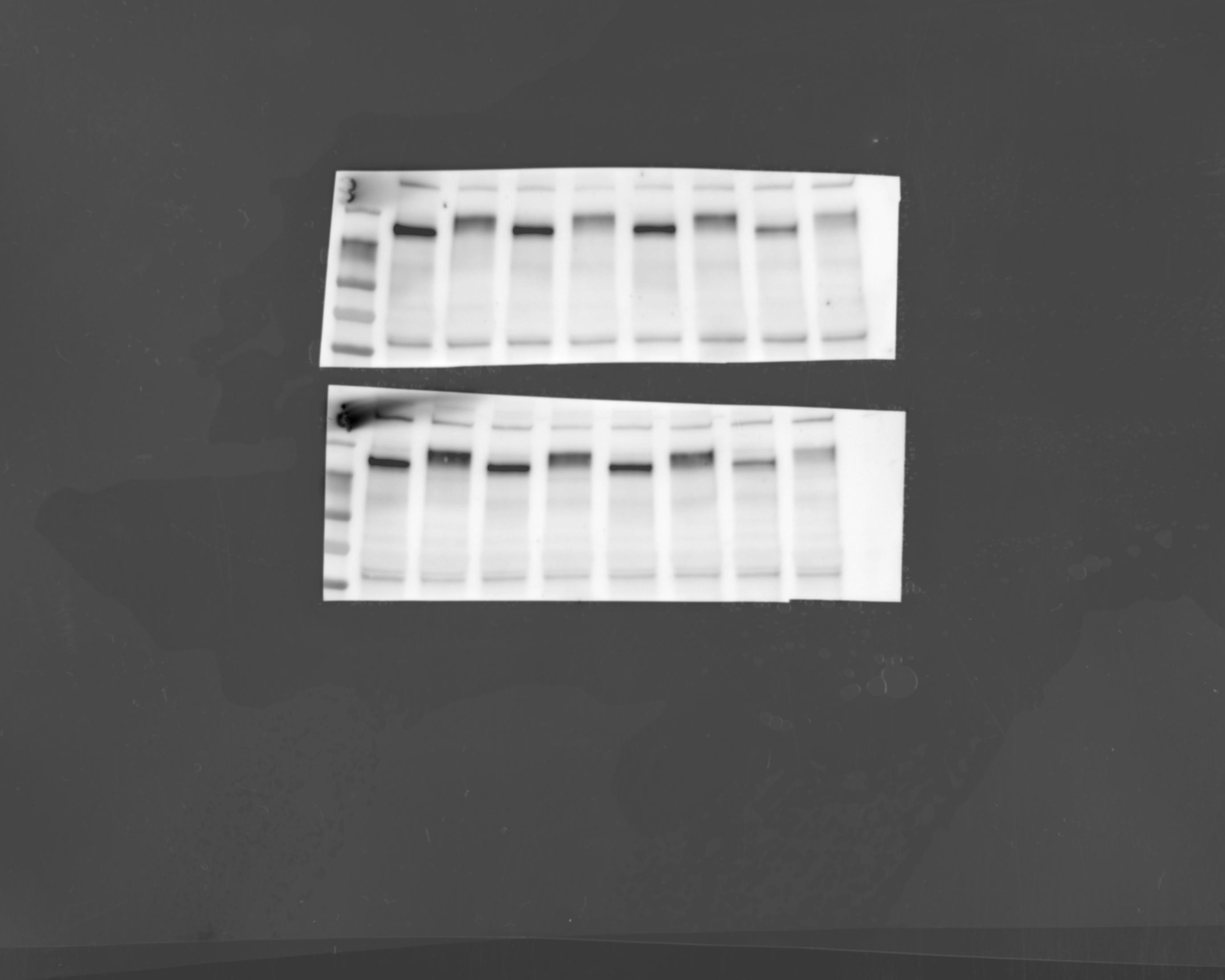

Supplement: Supplementary file 7 — Appendix and EV Figures Source Data [file 44319_2024_95_MOESM7_ESM.zip › Figure_EV4_SD/EV4A source data/EV4A individual files/perk-mark.tif]

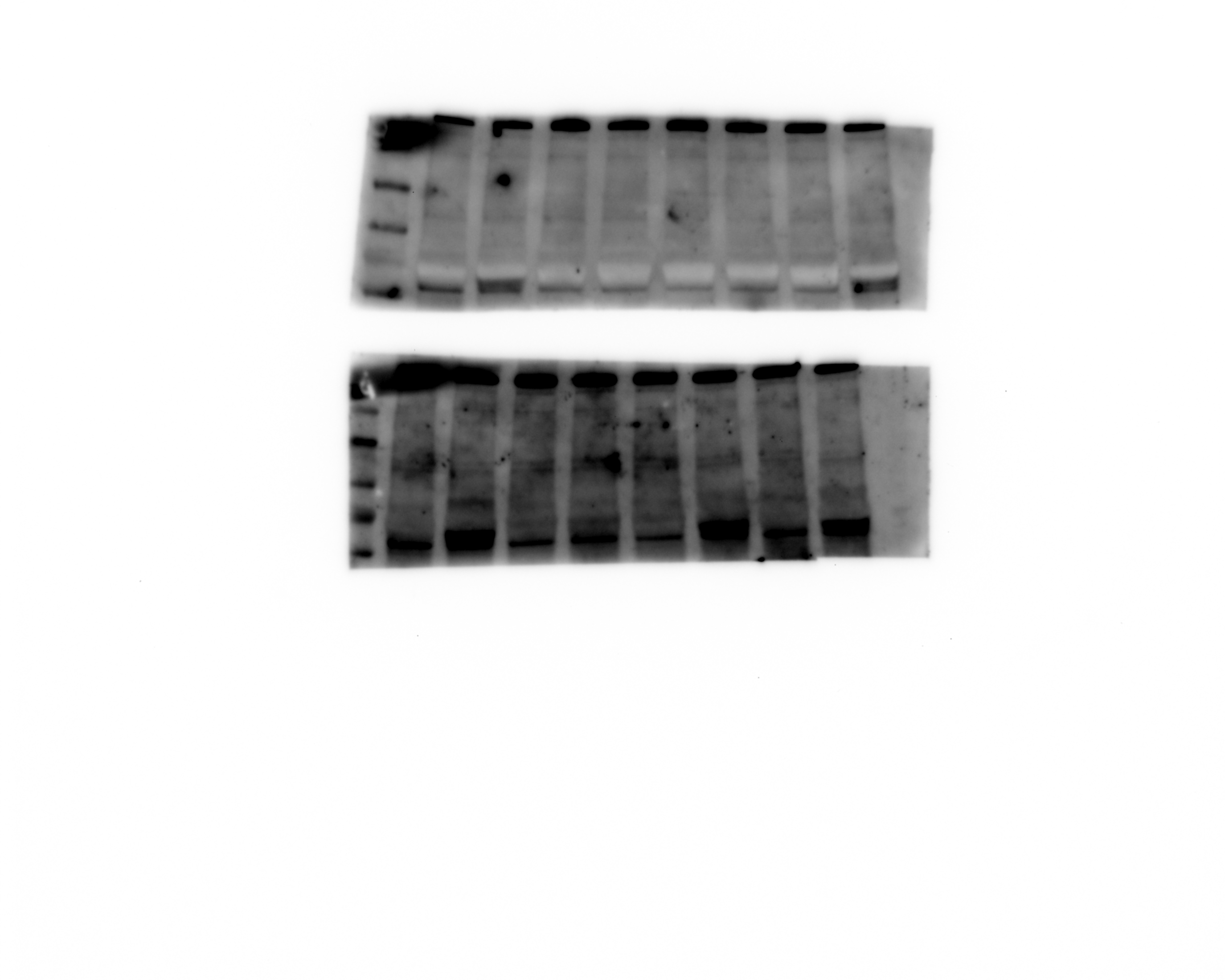

Supplement: Supplementary file 7 — Appendix and EV Figures Source Data [file 44319_2024_95_MOESM7_ESM.zip › Figure_EV4_SD/EV4A source data/EV4A individual files/xbp1s-3.tif]

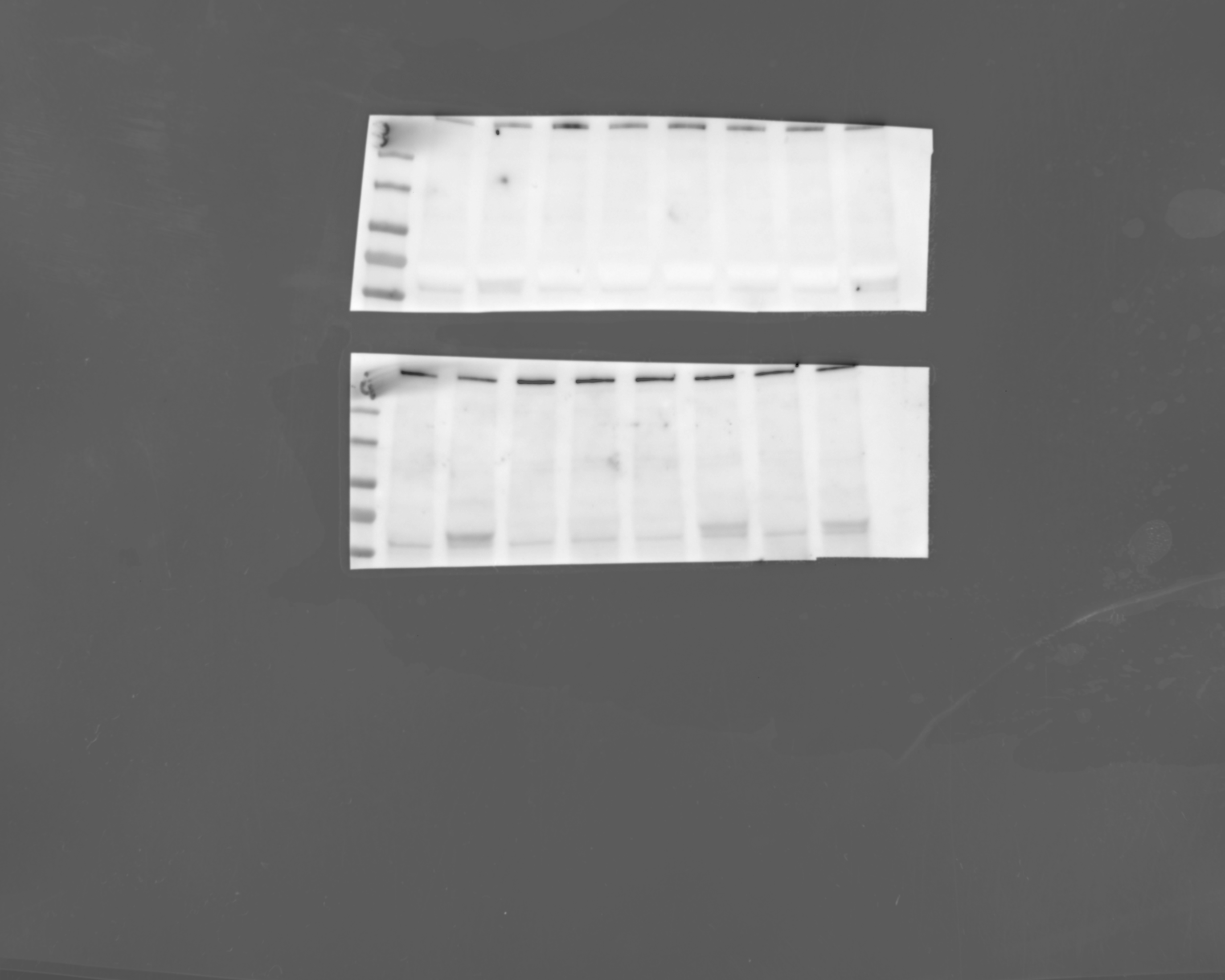

Supplement: Supplementary file 7 — Appendix and EV Figures Source Data [file 44319_2024_95_MOESM7_ESM.zip › Figure_EV4_SD/EV4A source data/EV4A individual files/xbp1s-mark.tif]

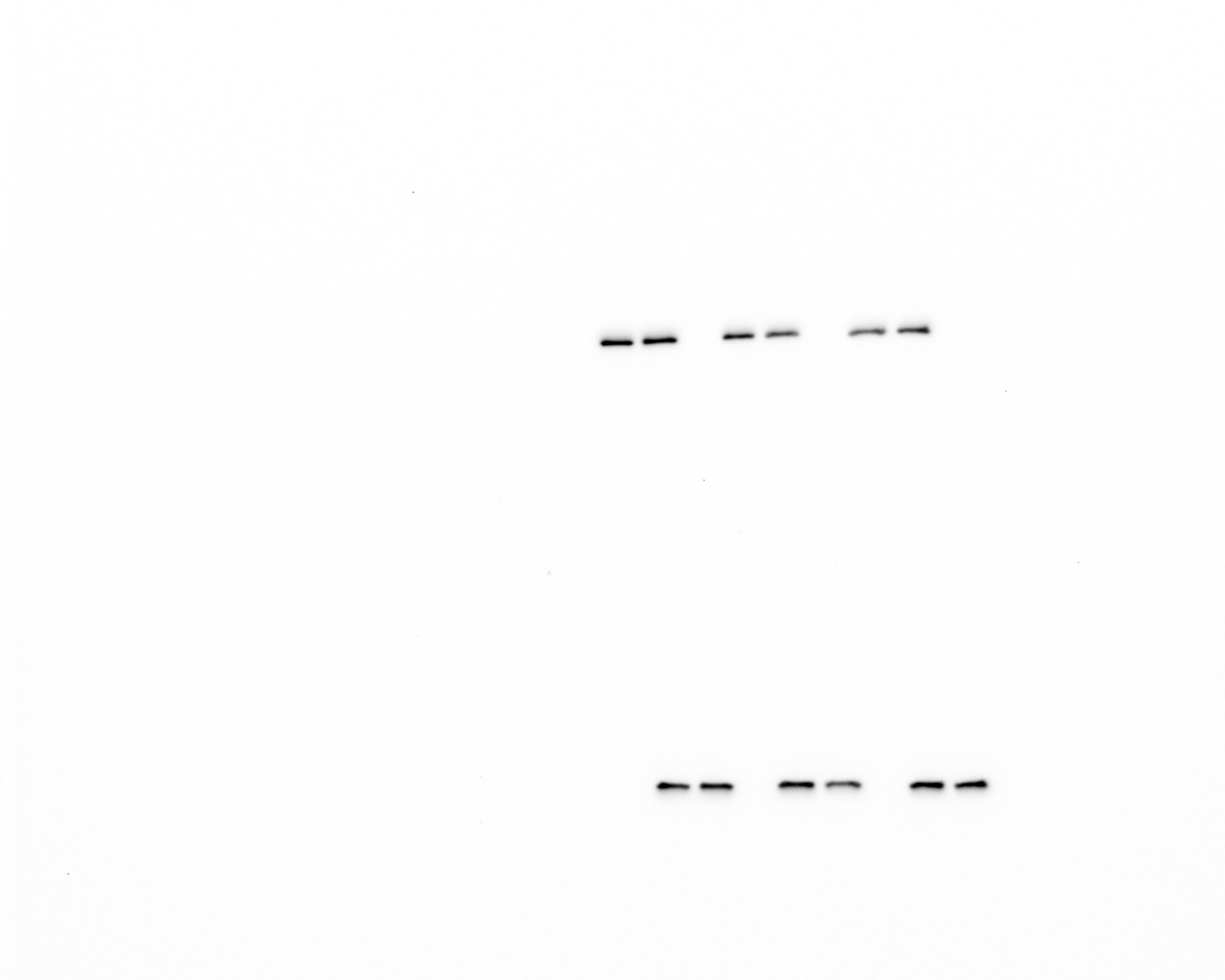

Supplement: Supplementary file 7 — Appendix and EV Figures Source Data [file 44319_2024_95_MOESM7_ESM.zip › Figure_EV4_SD/EV4B source data/EV4B individual files/actin-1.tif]

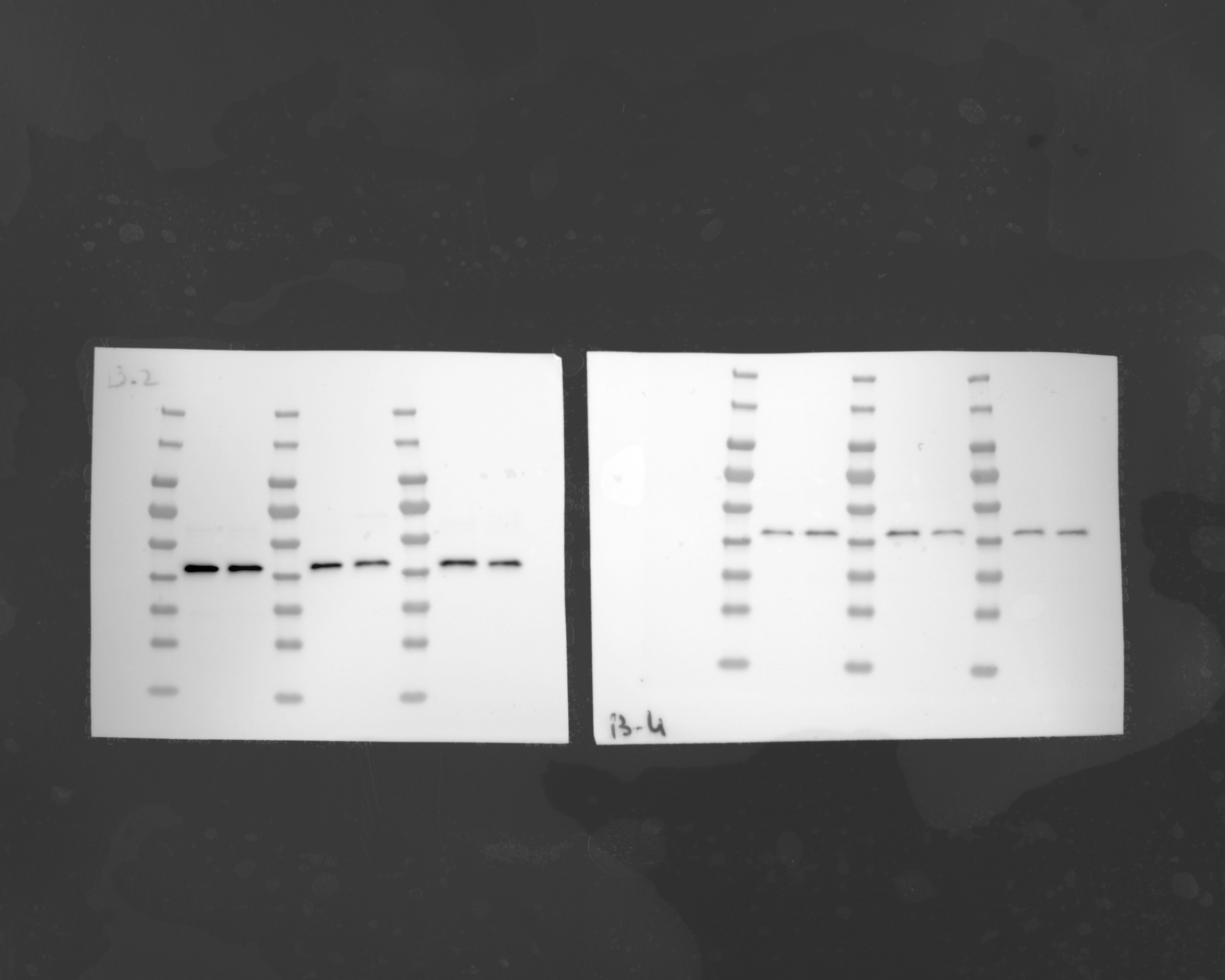

Supplement: Supplementary file 7 — Appendix and EV Figures Source Data [file 44319_2024_95_MOESM7_ESM.zip › Figure_EV4_SD/EV4B source data/EV4B individual files/actin-forXBP1s (not shown).tif]

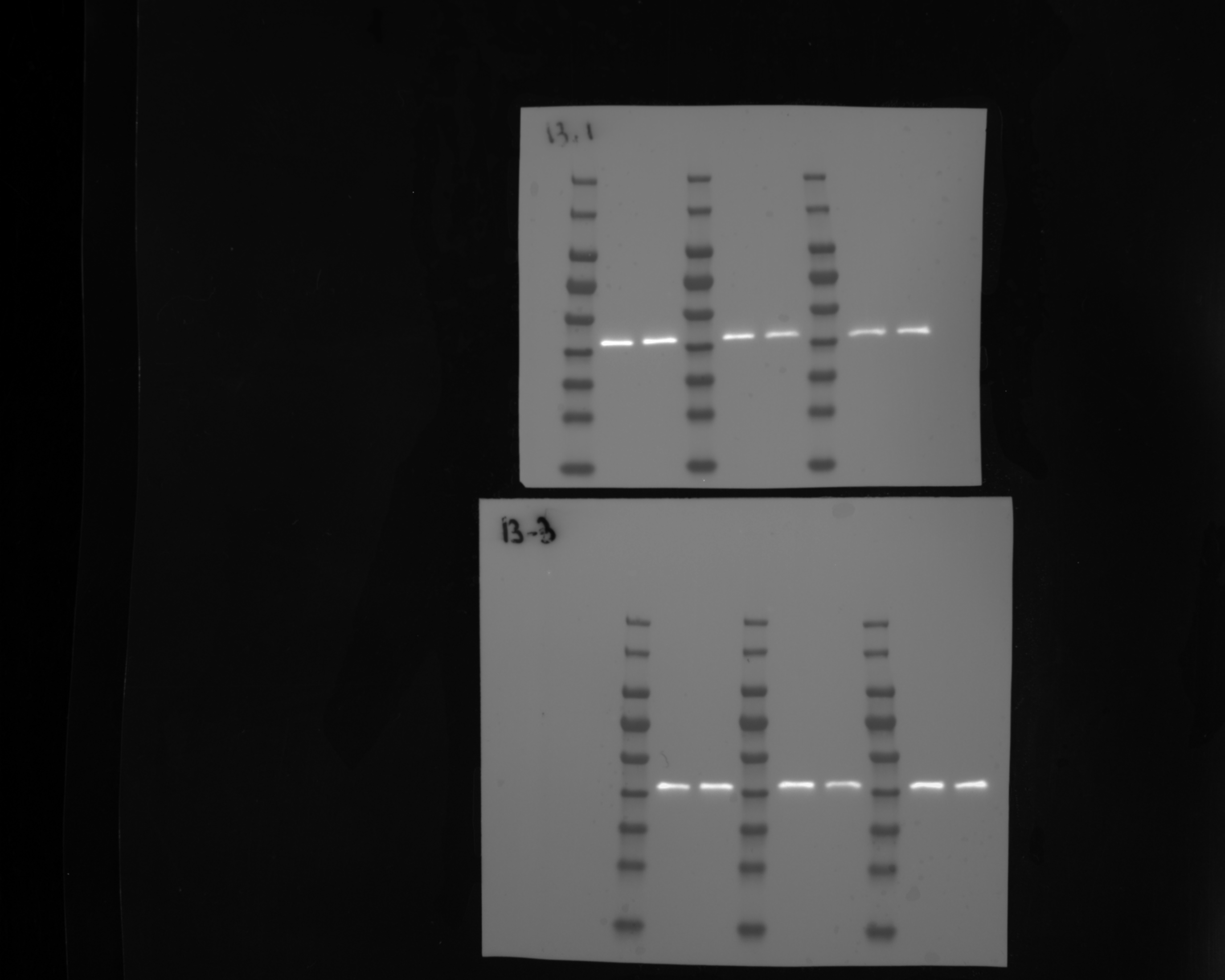

Supplement: Supplementary file 7 — Appendix and EV Figures Source Data [file 44319_2024_95_MOESM7_ESM.zip › Figure_EV4_SD/EV4B source data/EV4B individual files/actin-mark.tif]

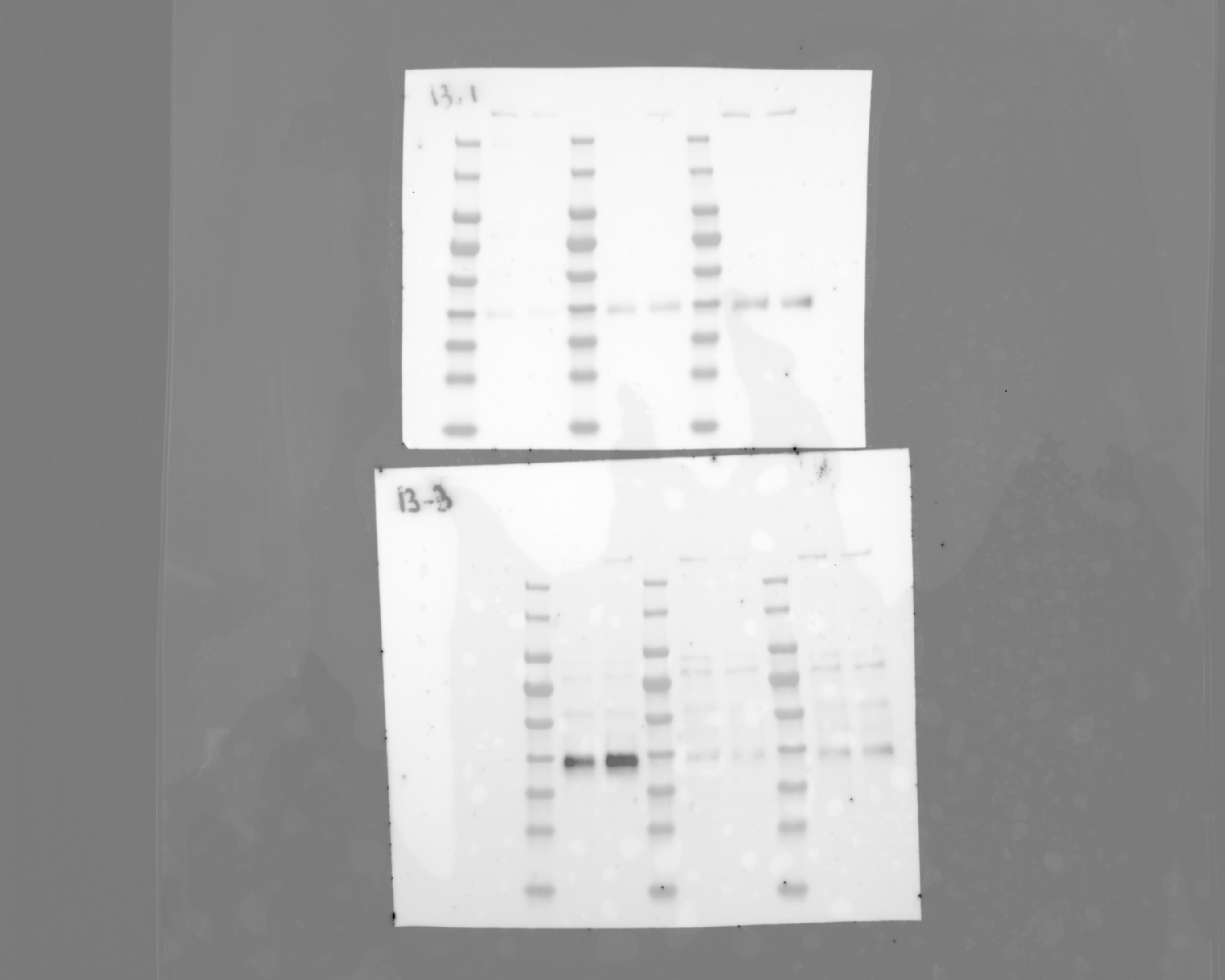

Supplement: Supplementary file 7 — Appendix and EV Figures Source Data [file 44319_2024_95_MOESM7_ESM.zip › Figure_EV4_SD/EV4B source data/EV4B individual files/cd95 mark.tif]

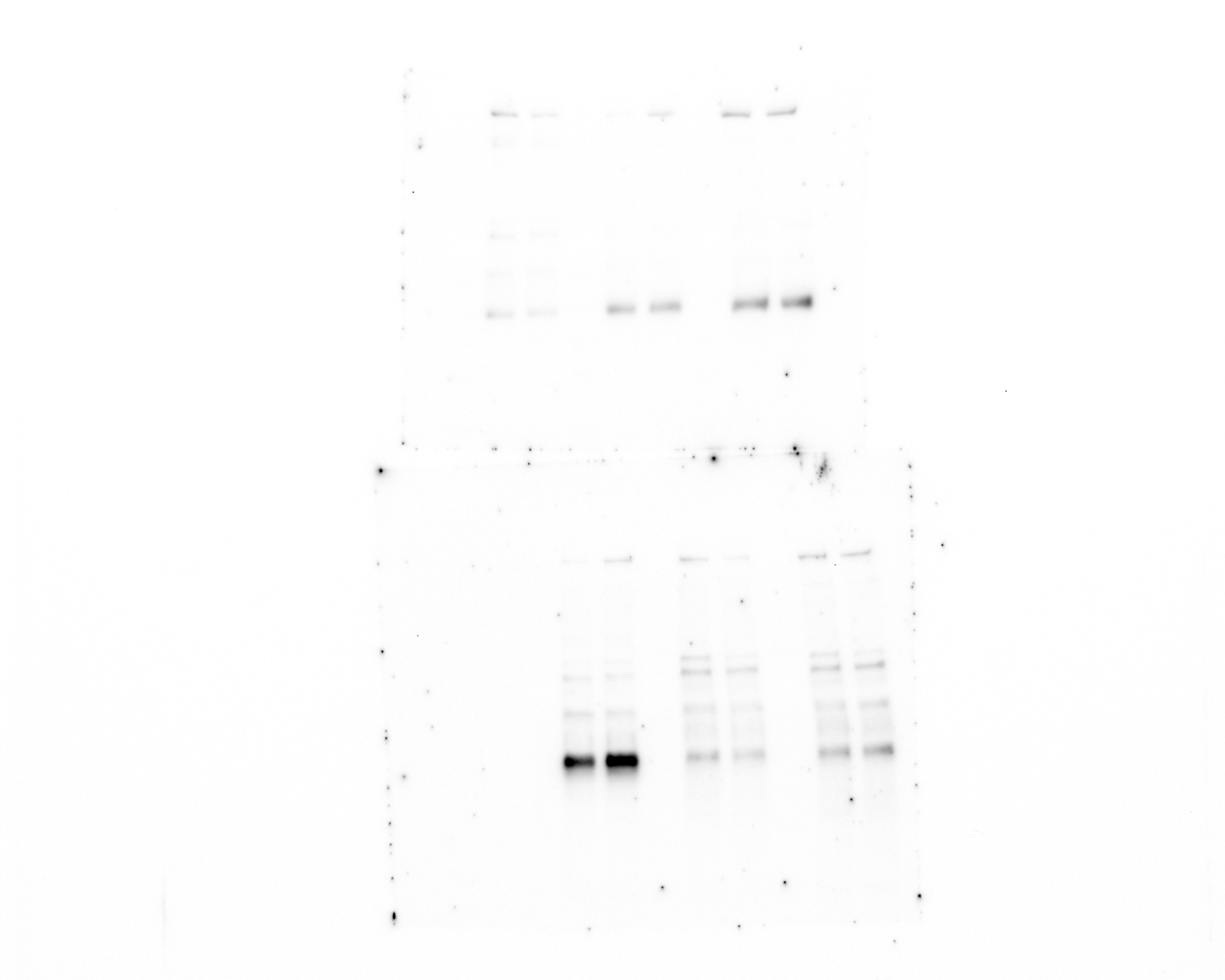

Supplement: Supplementary file 7 — Appendix and EV Figures Source Data [file 44319_2024_95_MOESM7_ESM.zip › Figure_EV4_SD/EV4B source data/EV4B individual files/cd95-1.tif]

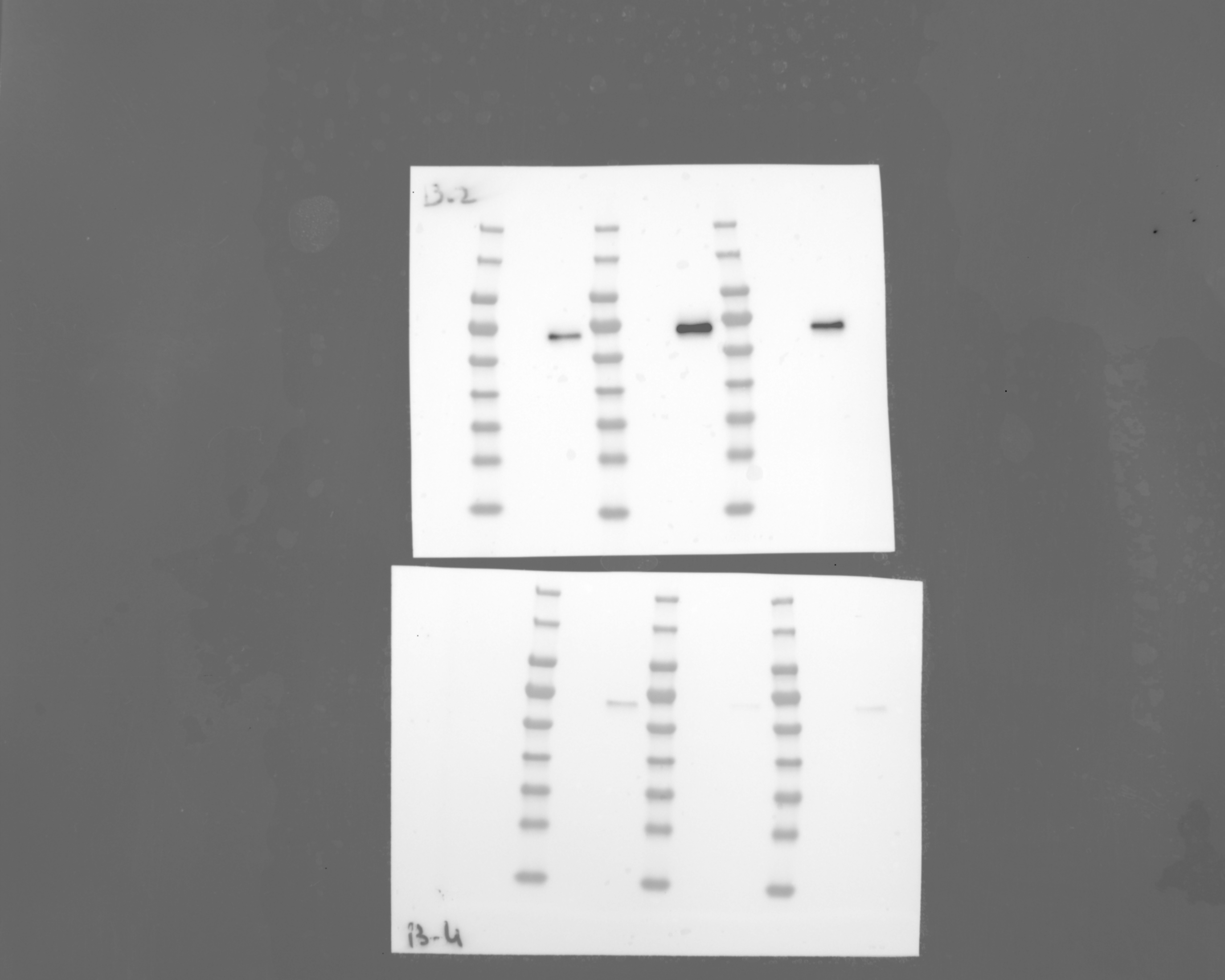

Supplement: Supplementary file 7 — Appendix and EV Figures Source Data [file 44319_2024_95_MOESM7_ESM.zip › Figure_EV4_SD/EV4B source data/EV4B individual files/xbp1s mark.tif]

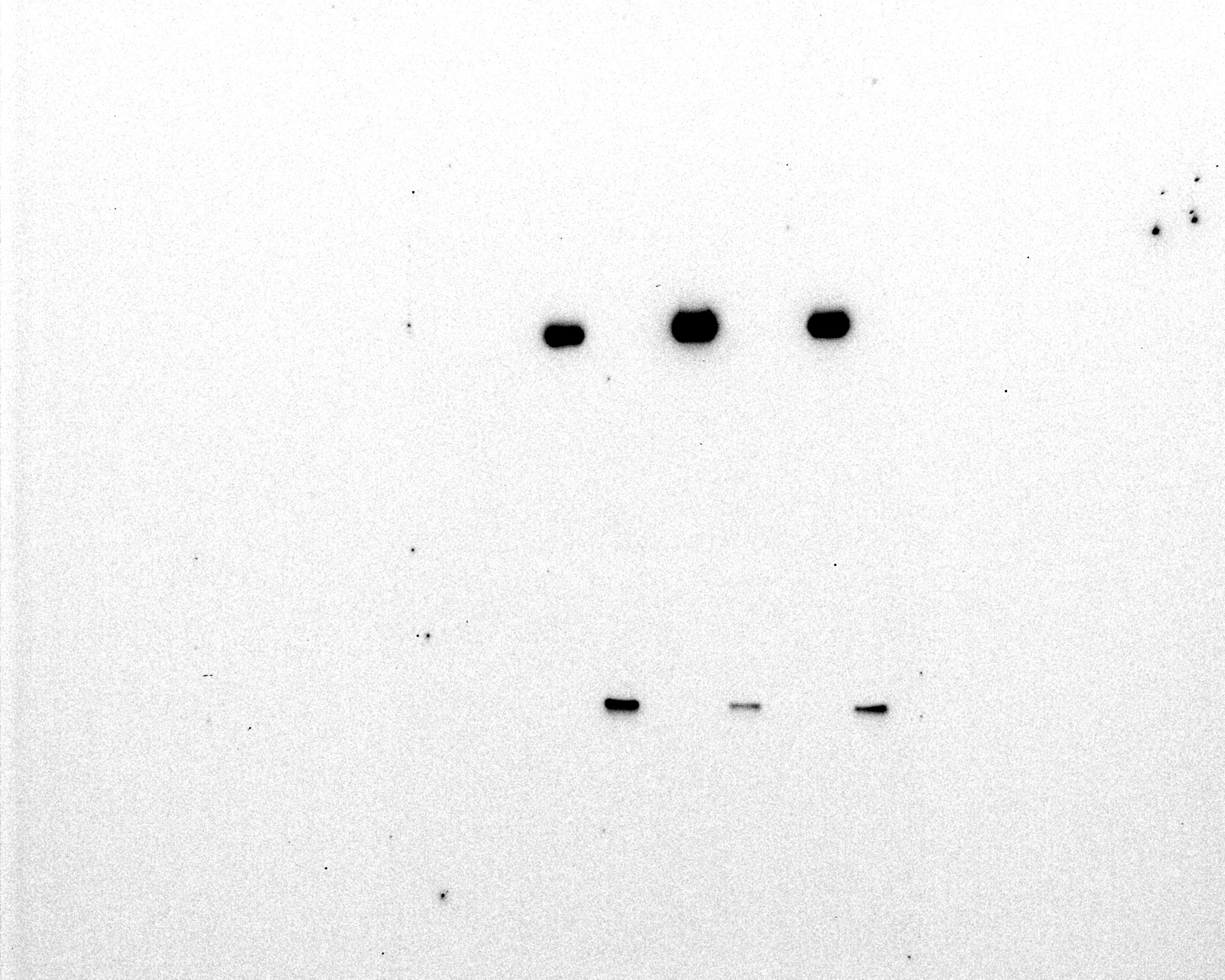

Supplement: Supplementary file 7 — Appendix and EV Figures Source Data [file 44319_2024_95_MOESM7_ESM.zip › Figure_EV4_SD/EV4B source data/EV4B individual files/xbp1s-2.tif]

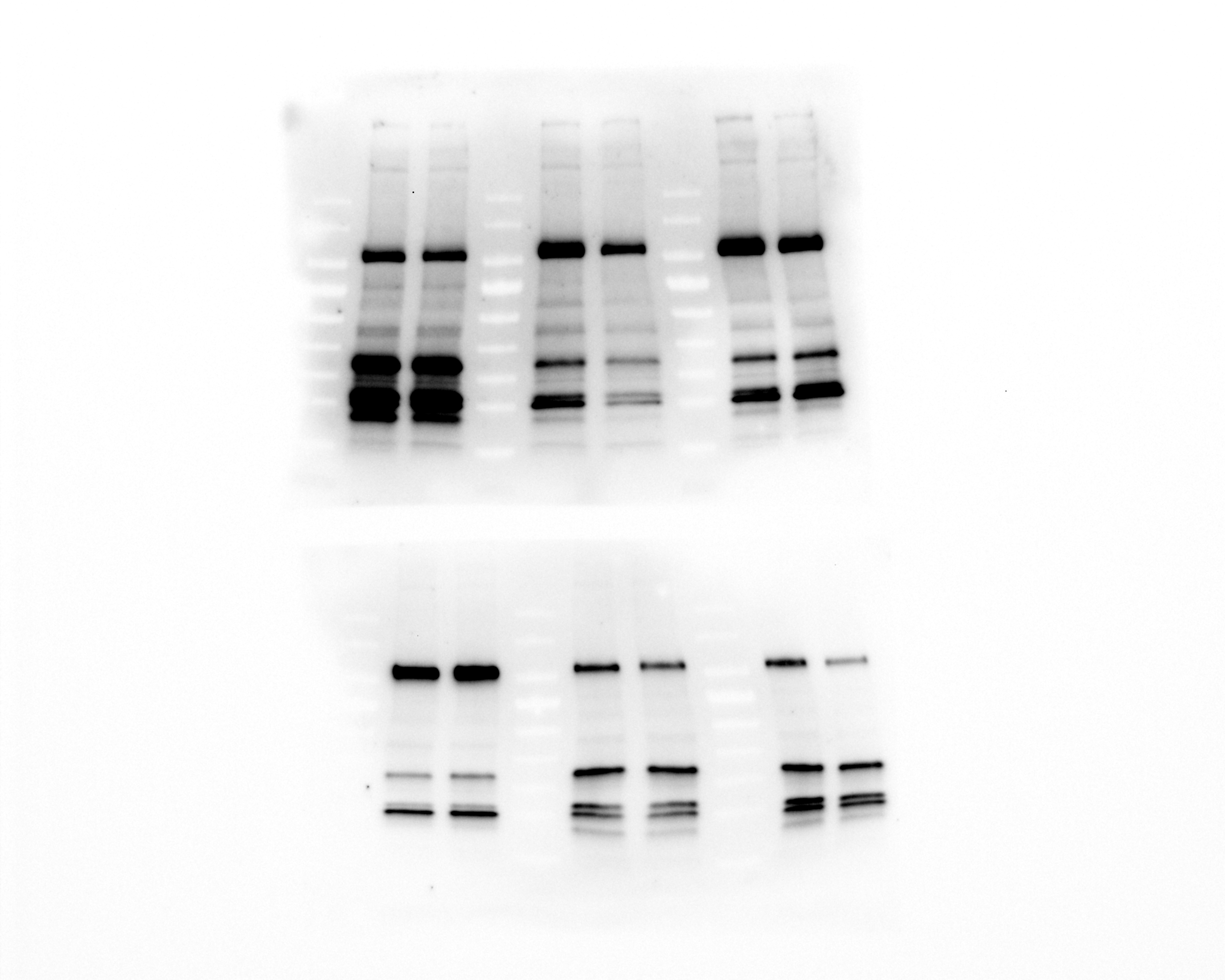

Supplement: Supplementary file 7 — Appendix and EV Figures Source Data [file 44319_2024_95_MOESM7_ESM.zip › Appendix_S4_SD/S4A source data SUM159 panel/Individual files S4A/ caspase7-bottom.tif]

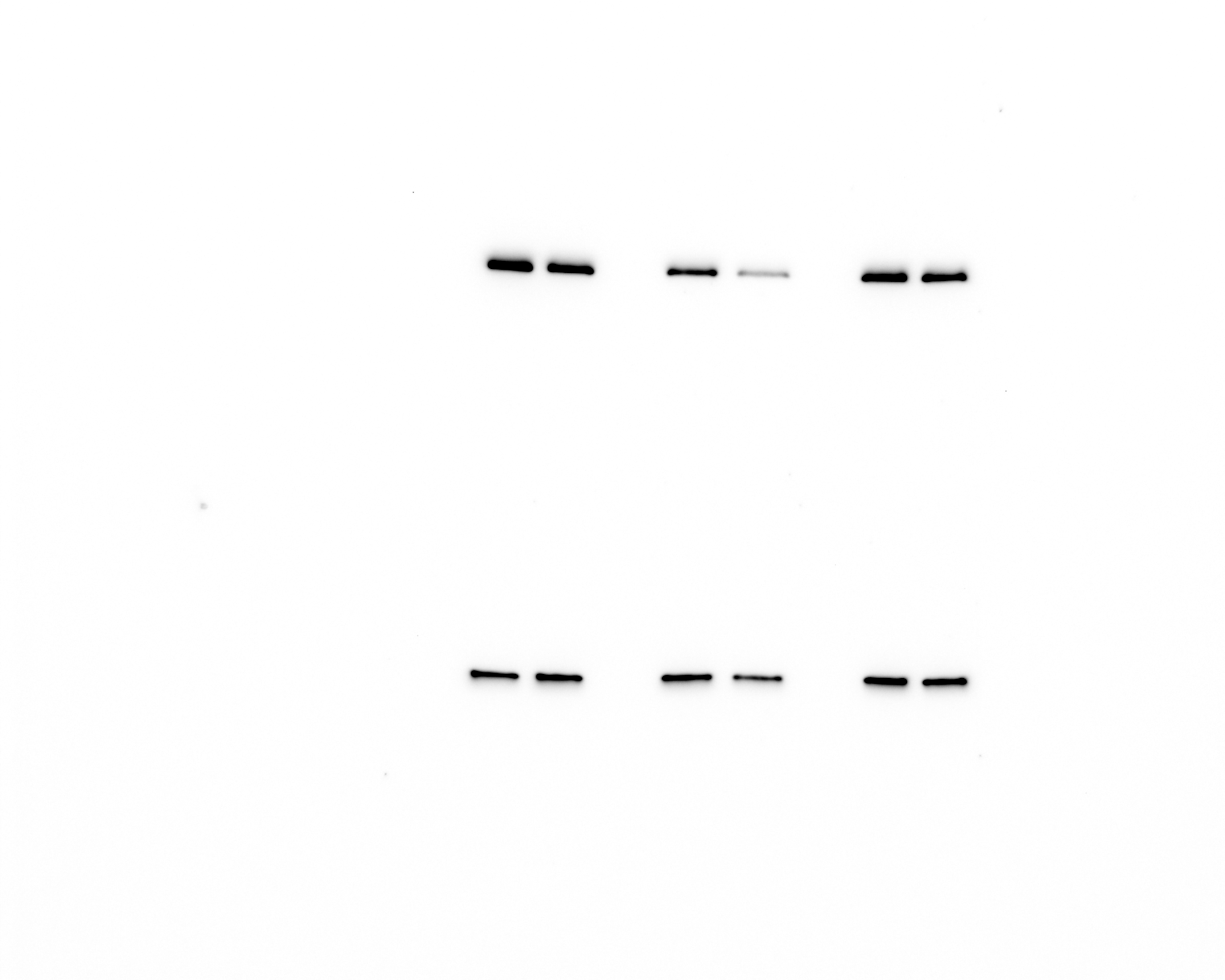

Supplement: Supplementary file 7 — Appendix and EV Figures Source Data [file 44319_2024_95_MOESM7_ESM.zip › Appendix_S4_SD/S4A source data SUM159 panel/Individual files S4A/actin3 (not shown) for bid bak casp9 casp7 .tif]

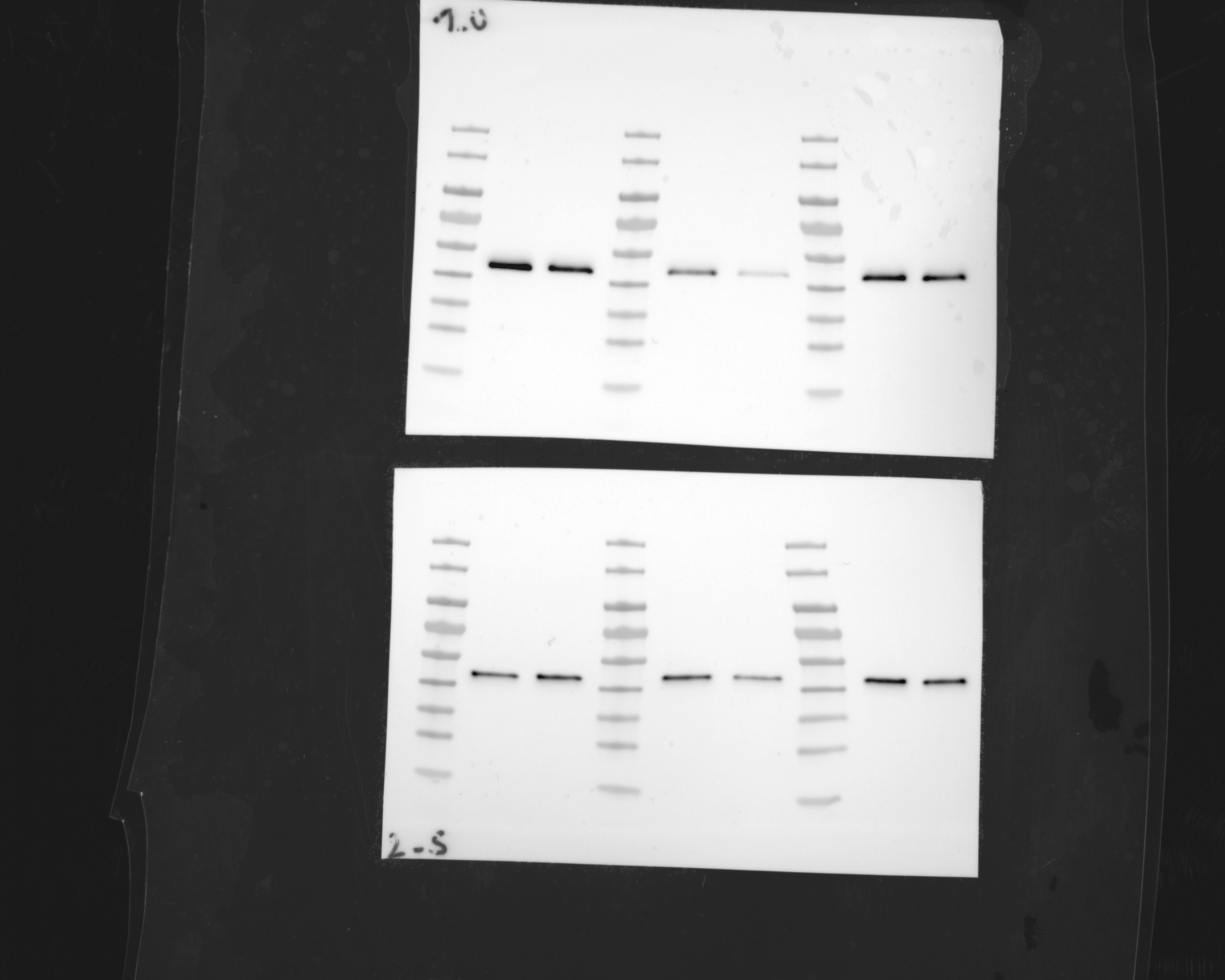

Supplement: Supplementary file 7 — Appendix and EV Figures Source Data [file 44319_2024_95_MOESM7_ESM.zip › Appendix_S4_SD/S4A source data SUM159 panel/Individual files S4A/actin3-mark.tif]

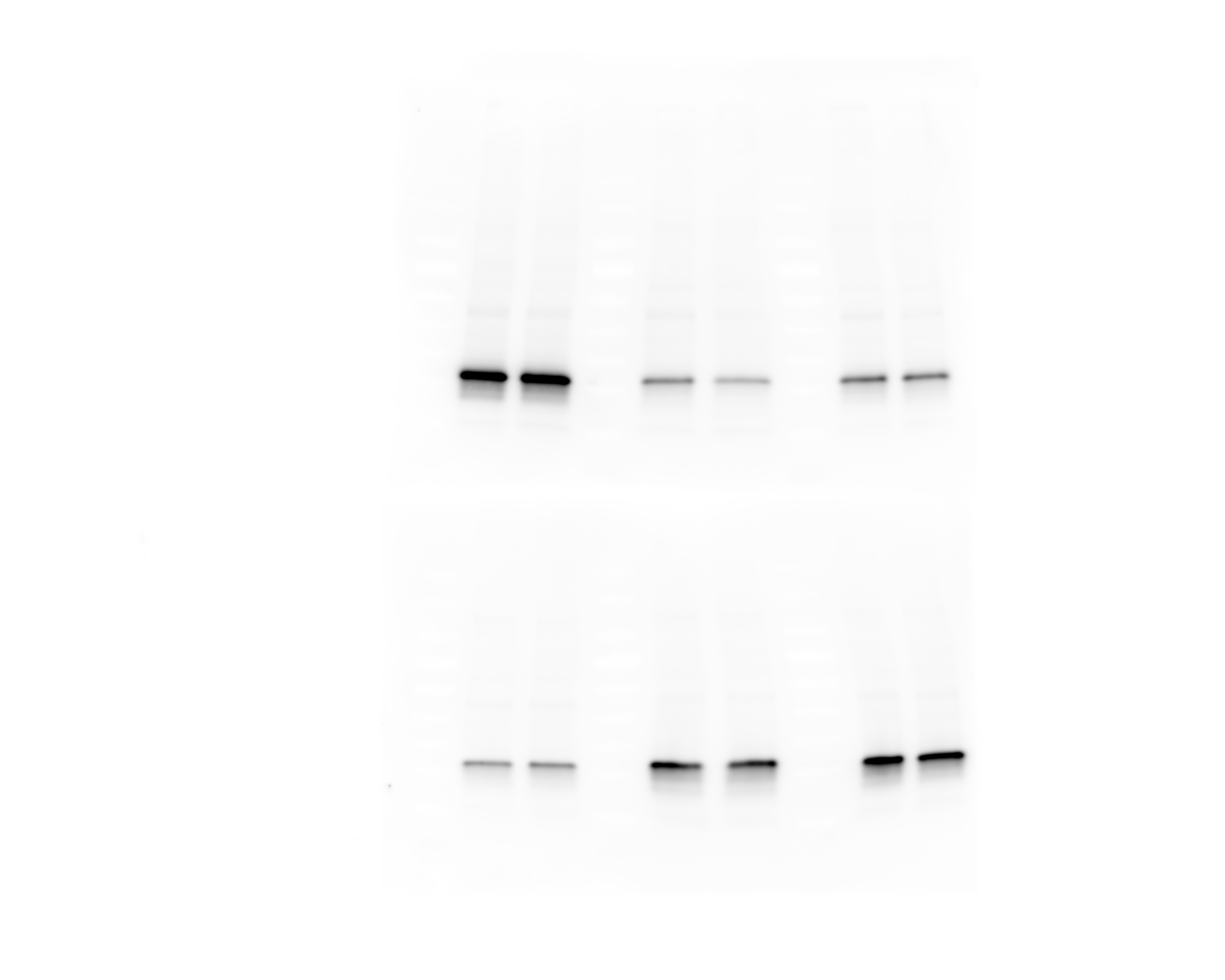

Supplement: Supplementary file 7 — Appendix and EV Figures Source Data [file 44319_2024_95_MOESM7_ESM.zip › Appendix_S4_SD/S4A source data SUM159 panel/Individual files S4A/bak-bottom.tif]

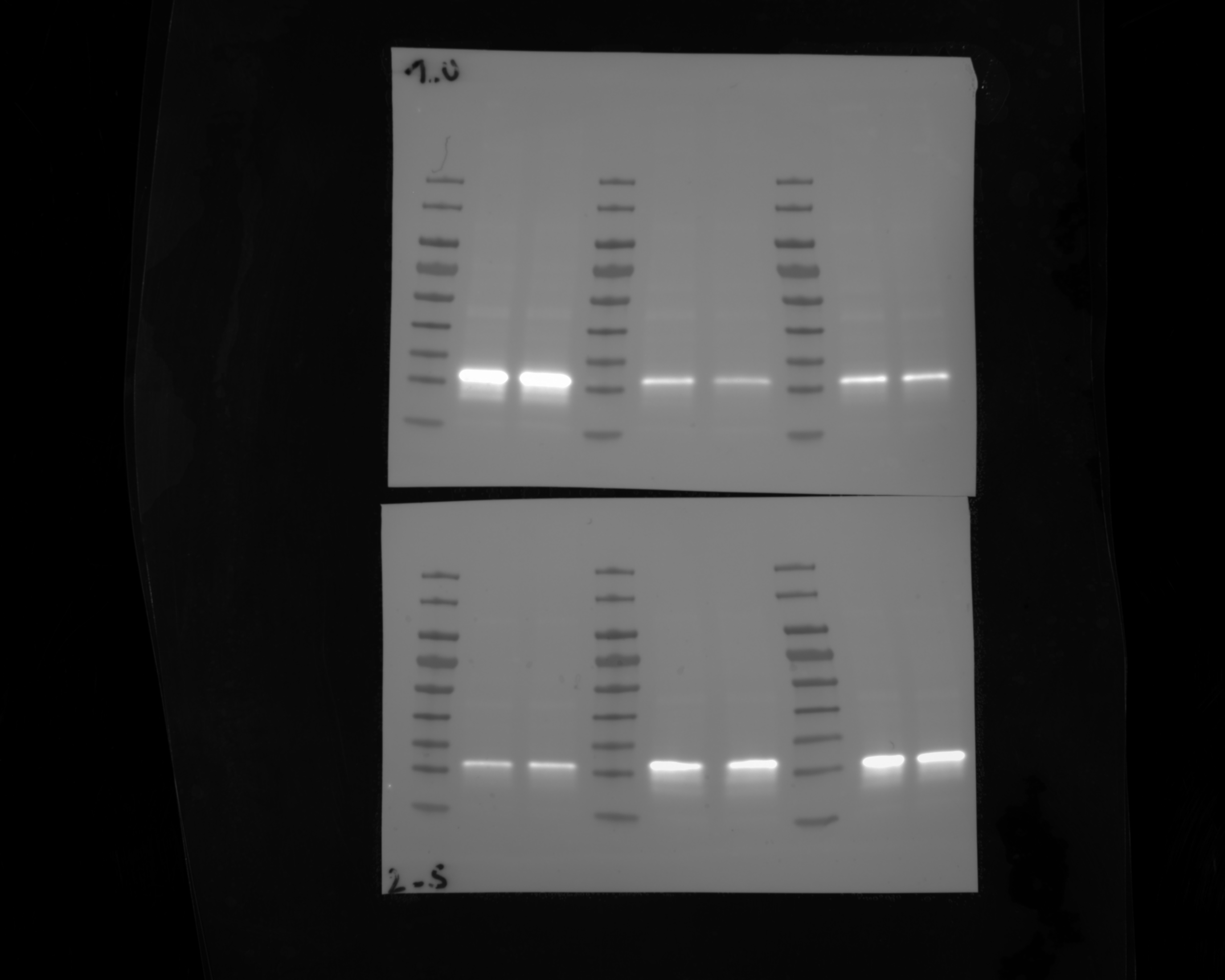

Supplement: Supplementary file 7 — Appendix and EV Figures Source Data [file 44319_2024_95_MOESM7_ESM.zip › Appendix_S4_SD/S4A source data SUM159 panel/Individual files S4A/bak-mark.tif]

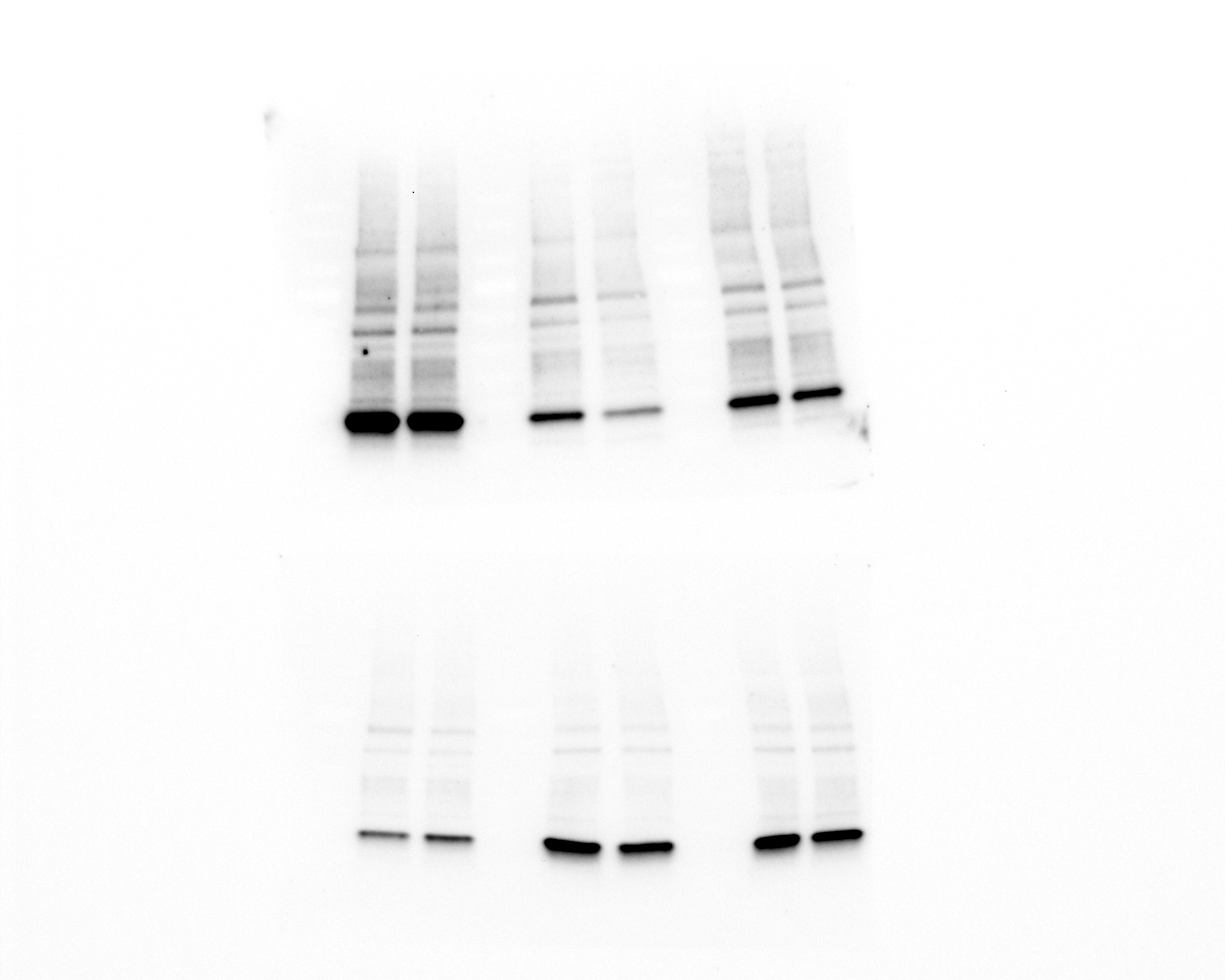

Supplement: Supplementary file 7 — Appendix and EV Figures Source Data [file 44319_2024_95_MOESM7_ESM.zip › Appendix_S4_SD/S4A source data SUM159 panel/Individual files S4A/bid-bottom.tif]

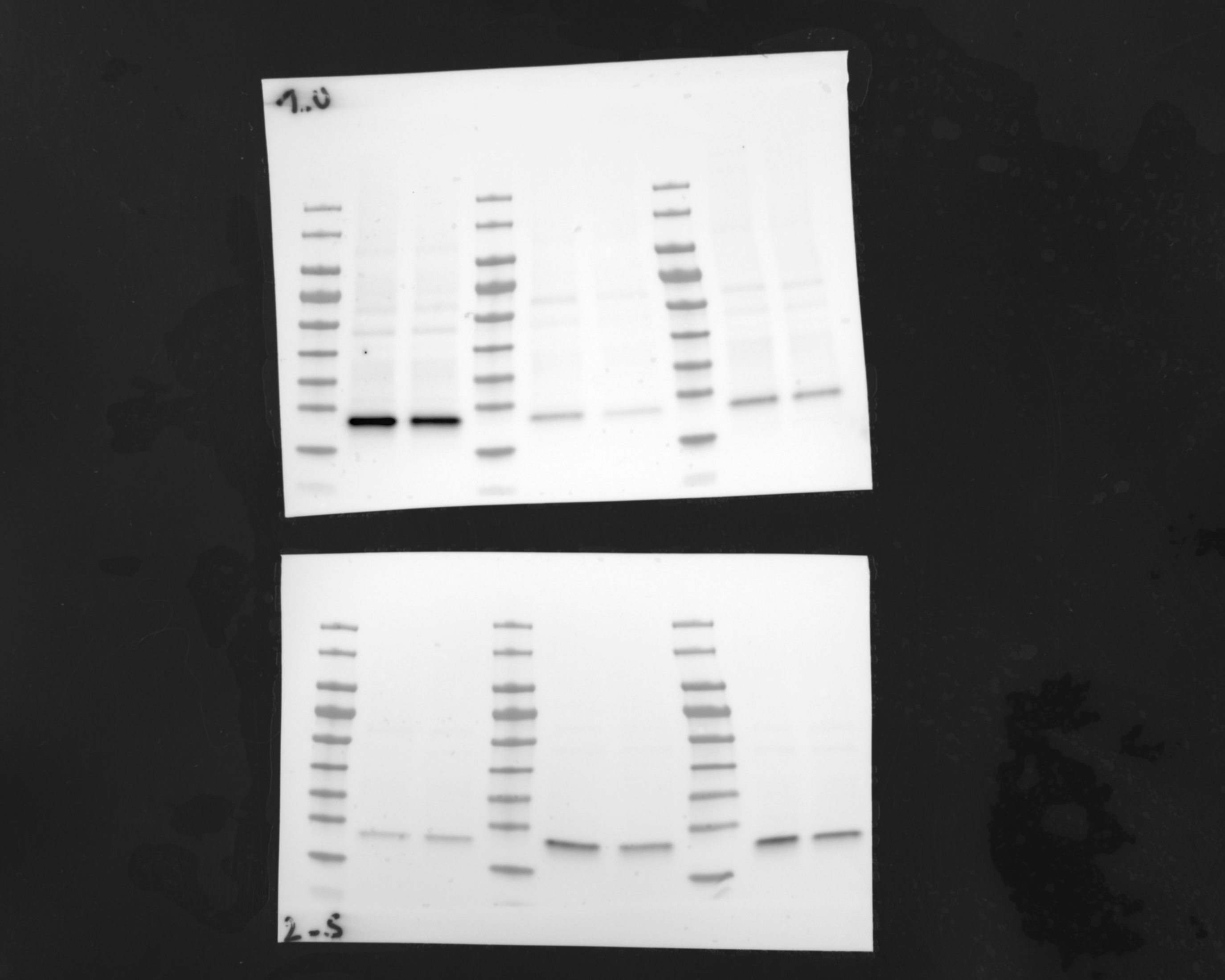

Supplement: Supplementary file 7 — Appendix and EV Figures Source Data [file 44319_2024_95_MOESM7_ESM.zip › Appendix_S4_SD/S4A source data SUM159 panel/Individual files S4A/bid-mark.tif]

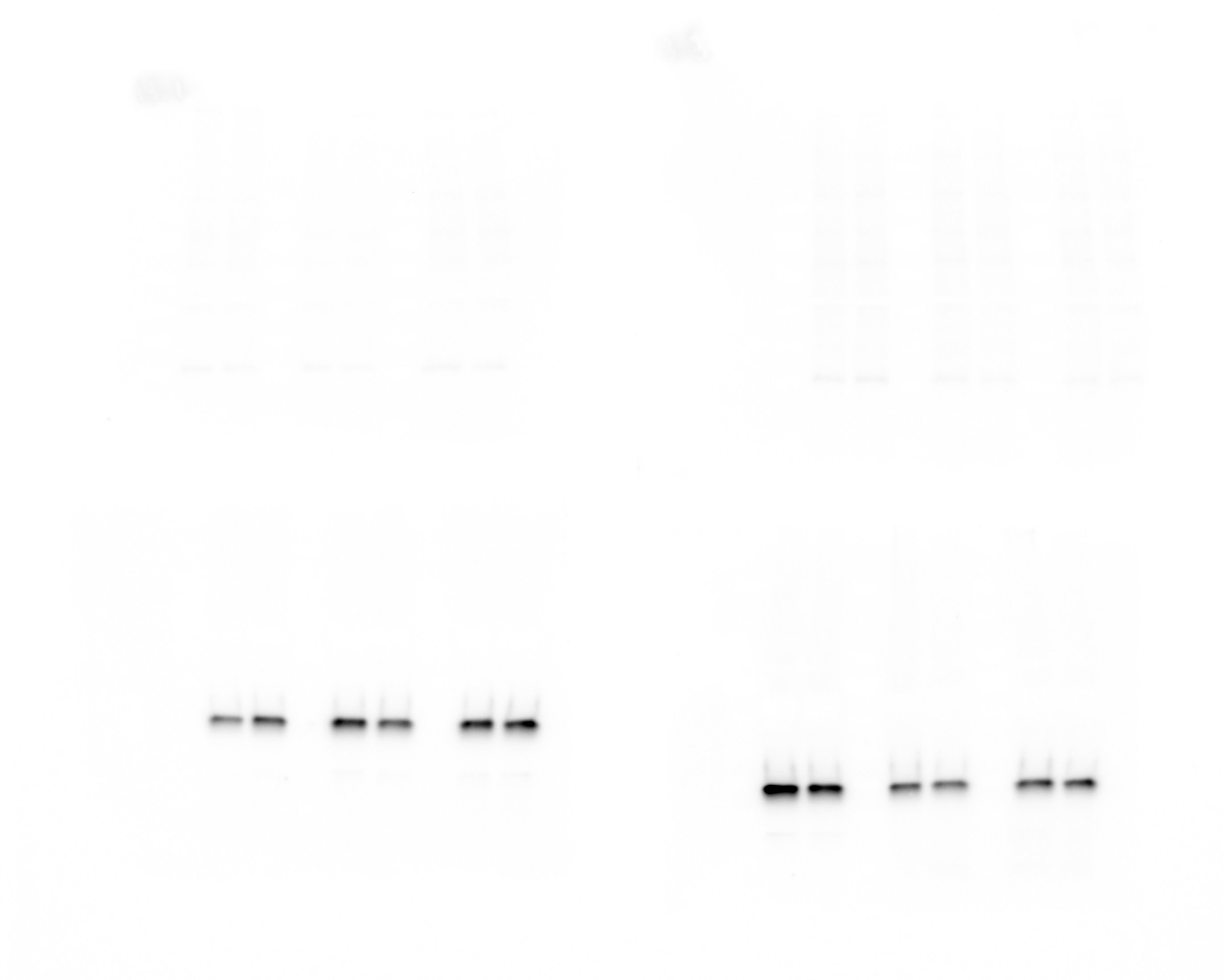

Supplement: Supplementary file 7 — Appendix and EV Figures Source Data [file 44319_2024_95_MOESM7_ESM.zip › Appendix_S4_SD/S4A source data SUM159 panel/Individual files S4A/caspase3_lower_left.tif]

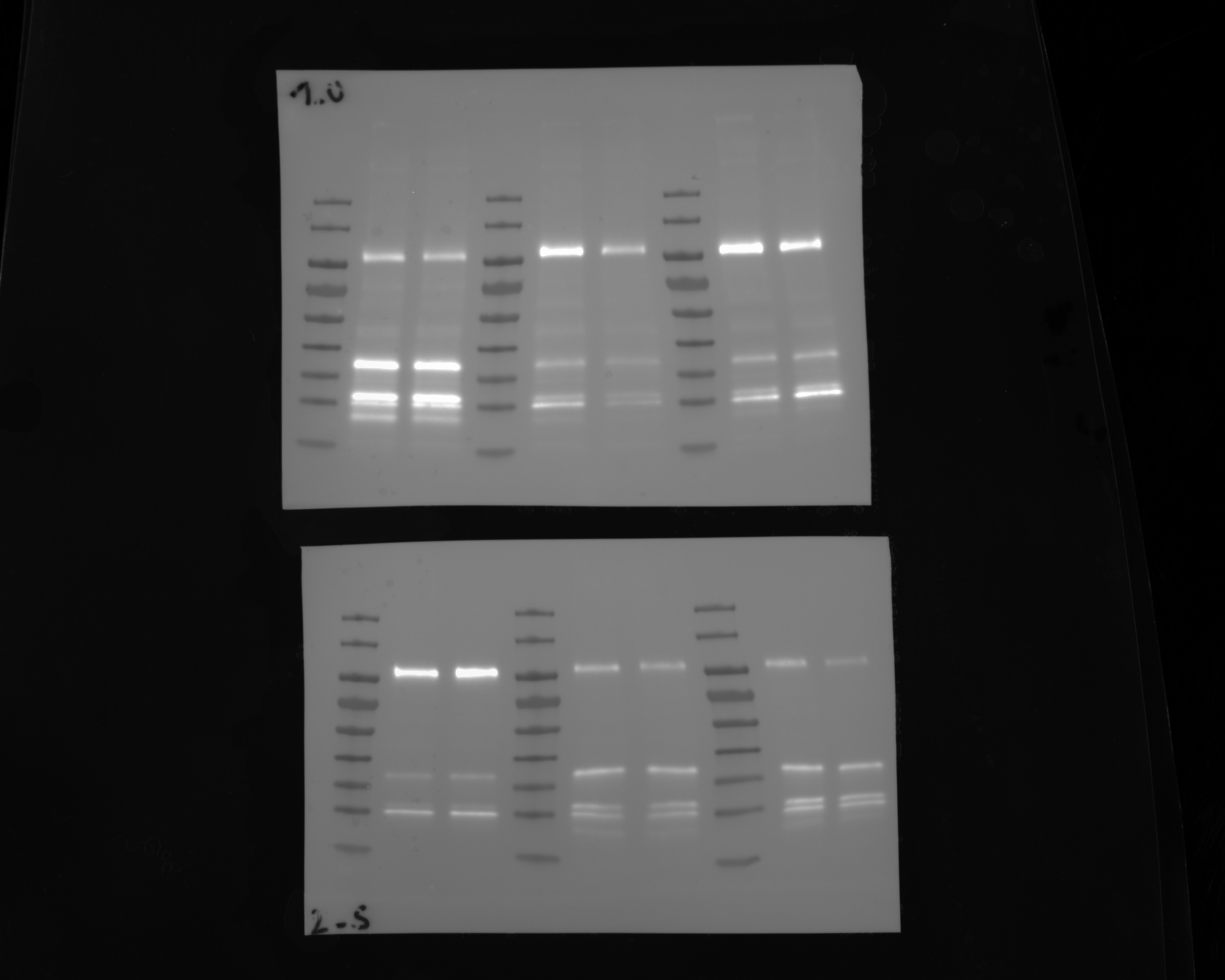

Supplement: Supplementary file 7 — Appendix and EV Figures Source Data [file 44319_2024_95_MOESM7_ESM.zip › Appendix_S4_SD/S4A source data SUM159 panel/Individual files S4A/caspase7-mark.tif]

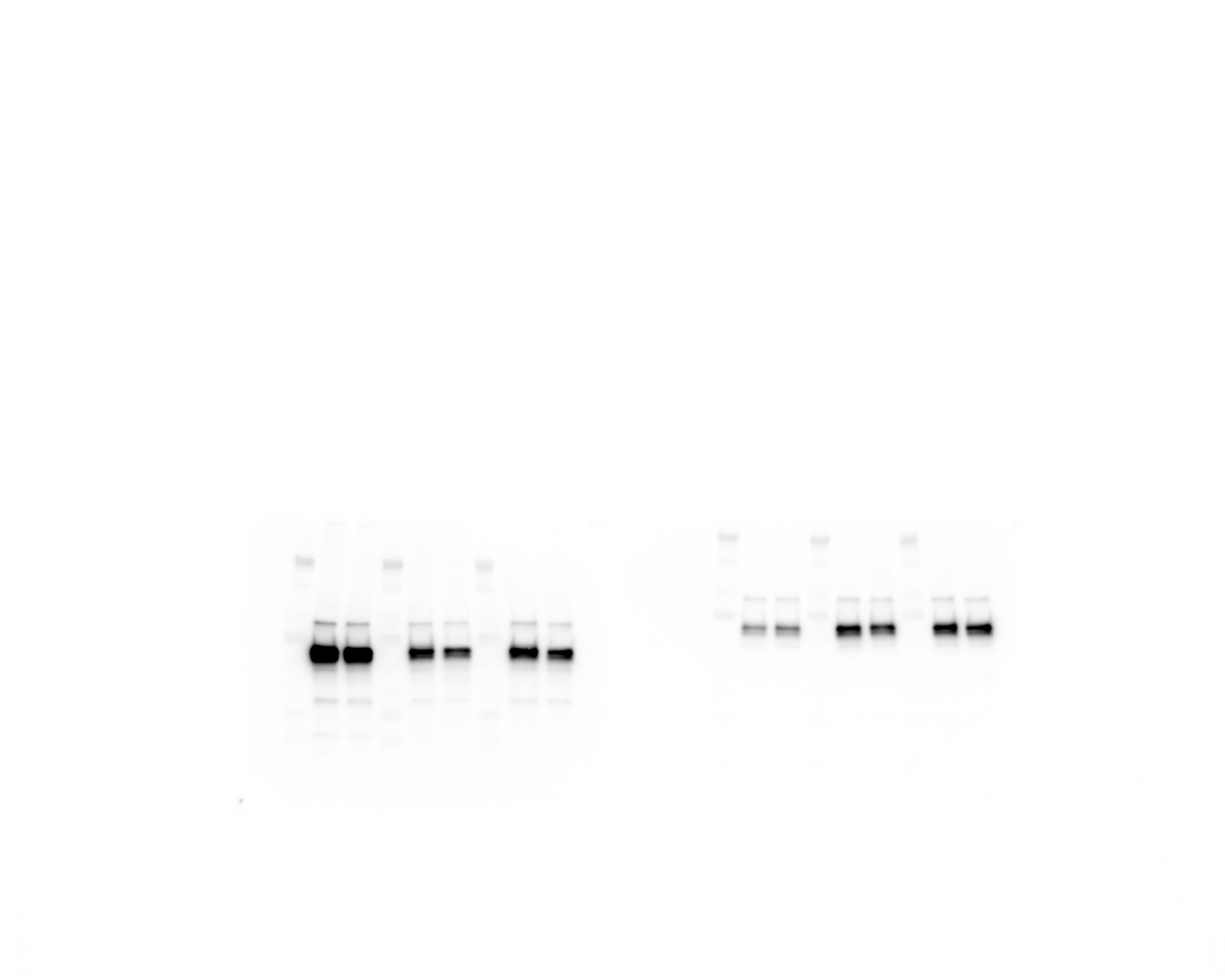

Supplement: Supplementary file 7 — Appendix and EV Figures Source Data [file 44319_2024_95_MOESM7_ESM.zip › Appendix_S4_SD/S4A source data SUM159 panel/Individual files S4A/caspase8_lower_right.tif]

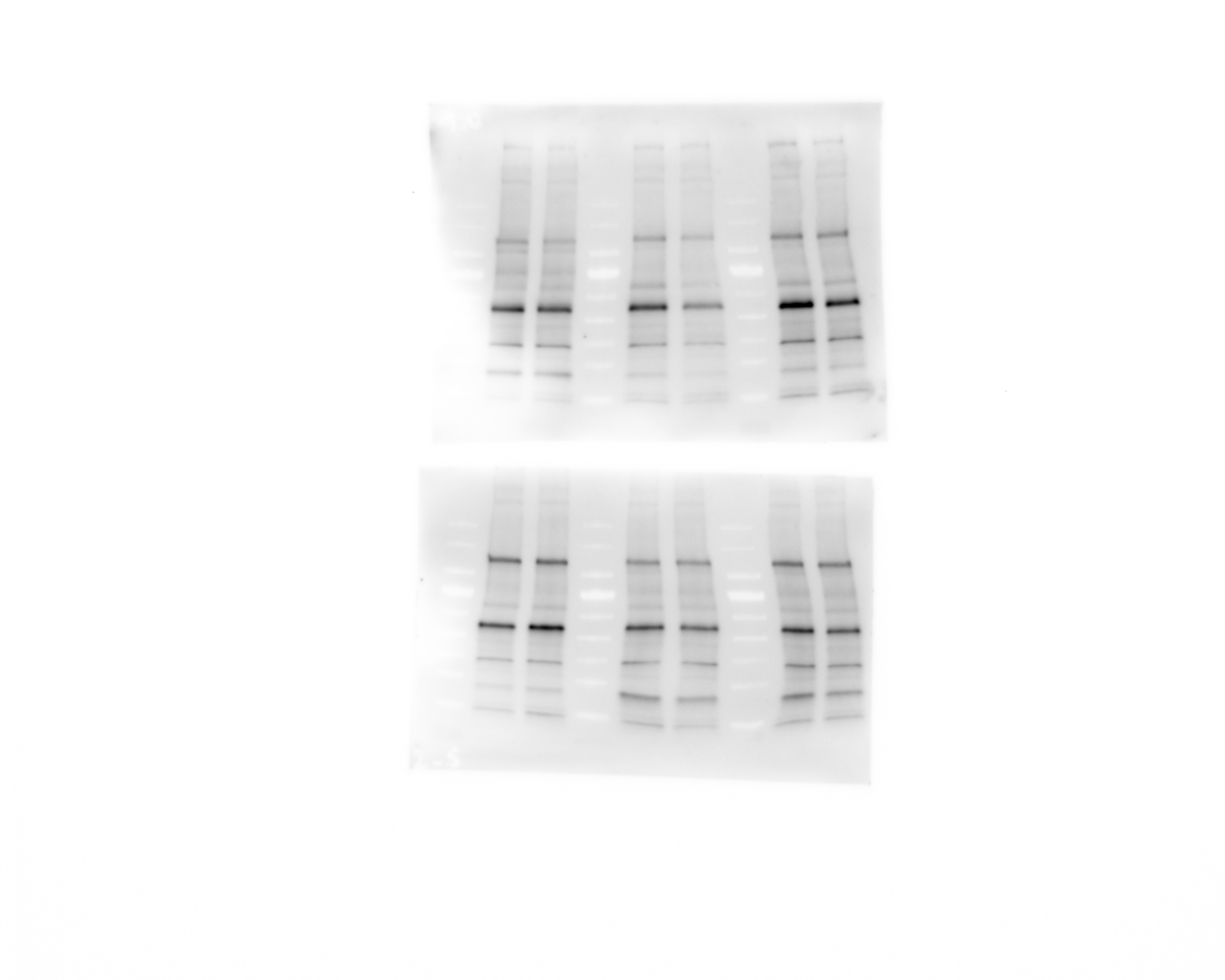

Supplement: Supplementary file 7 — Appendix and EV Figures Source Data [file 44319_2024_95_MOESM7_ESM.zip › Appendix_S4_SD/S4A source data SUM159 panel/Individual files S4A/caspase9-bottom.tif]

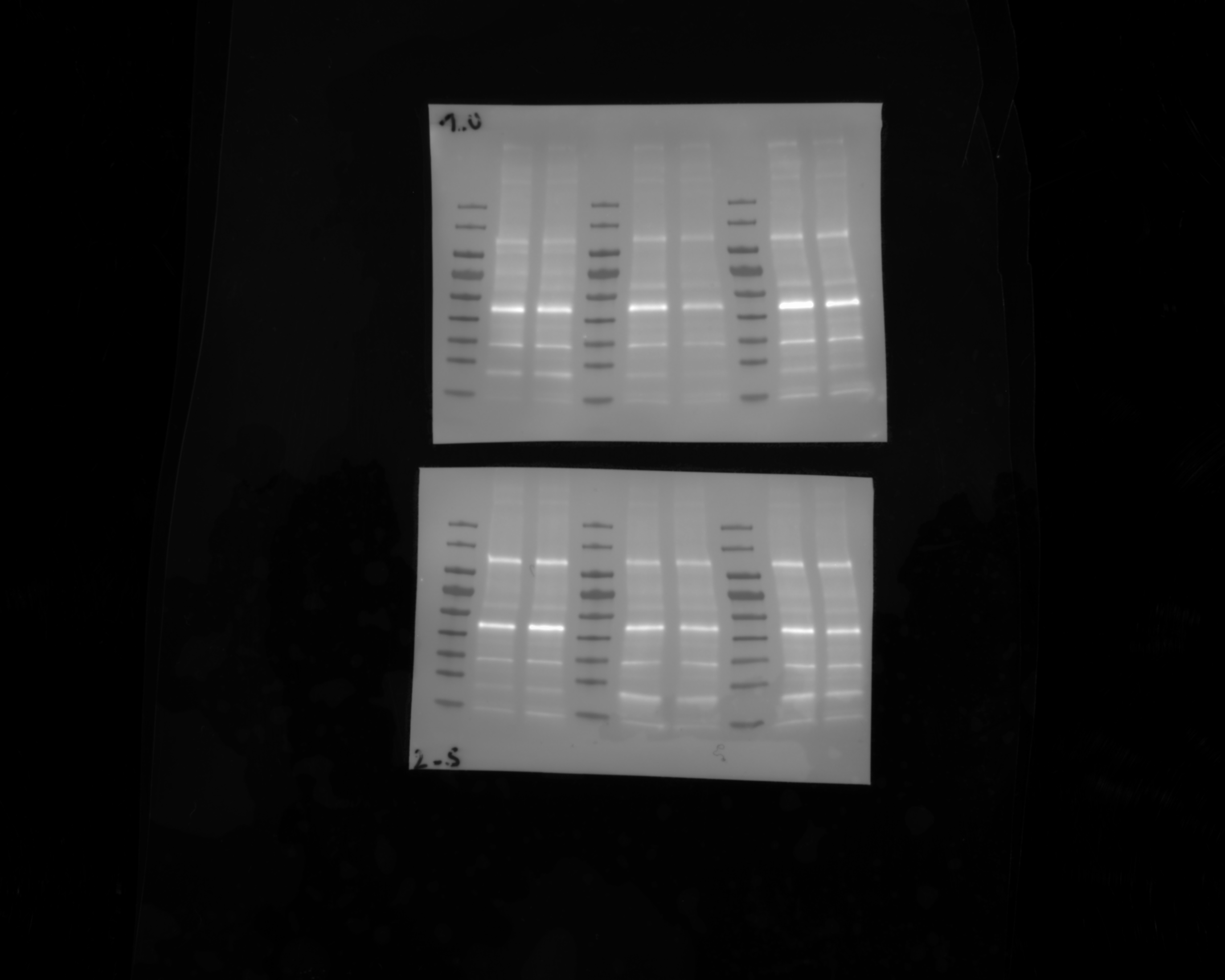

Supplement: Supplementary file 7 — Appendix and EV Figures Source Data [file 44319_2024_95_MOESM7_ESM.zip › Appendix_S4_SD/S4A source data SUM159 panel/Individual files S4A/caspase9mark.tif]

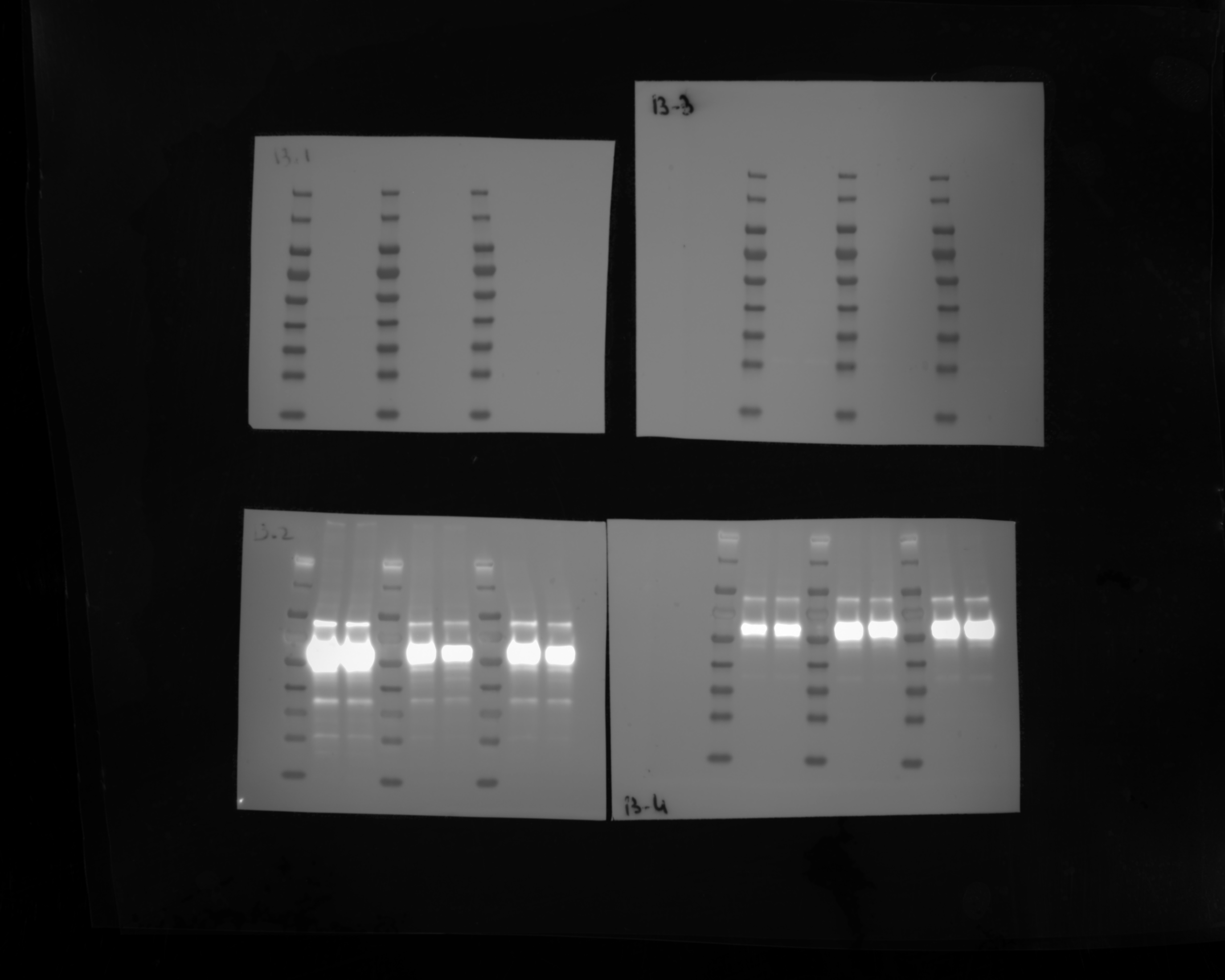

Supplement: Supplementary file 7 — Appendix and EV Figures Source Data [file 44319_2024_95_MOESM7_ESM.zip › Appendix_S4_SD/S4A source data SUM159 panel/Individual files S4A/fadd c8 -mark.tif]

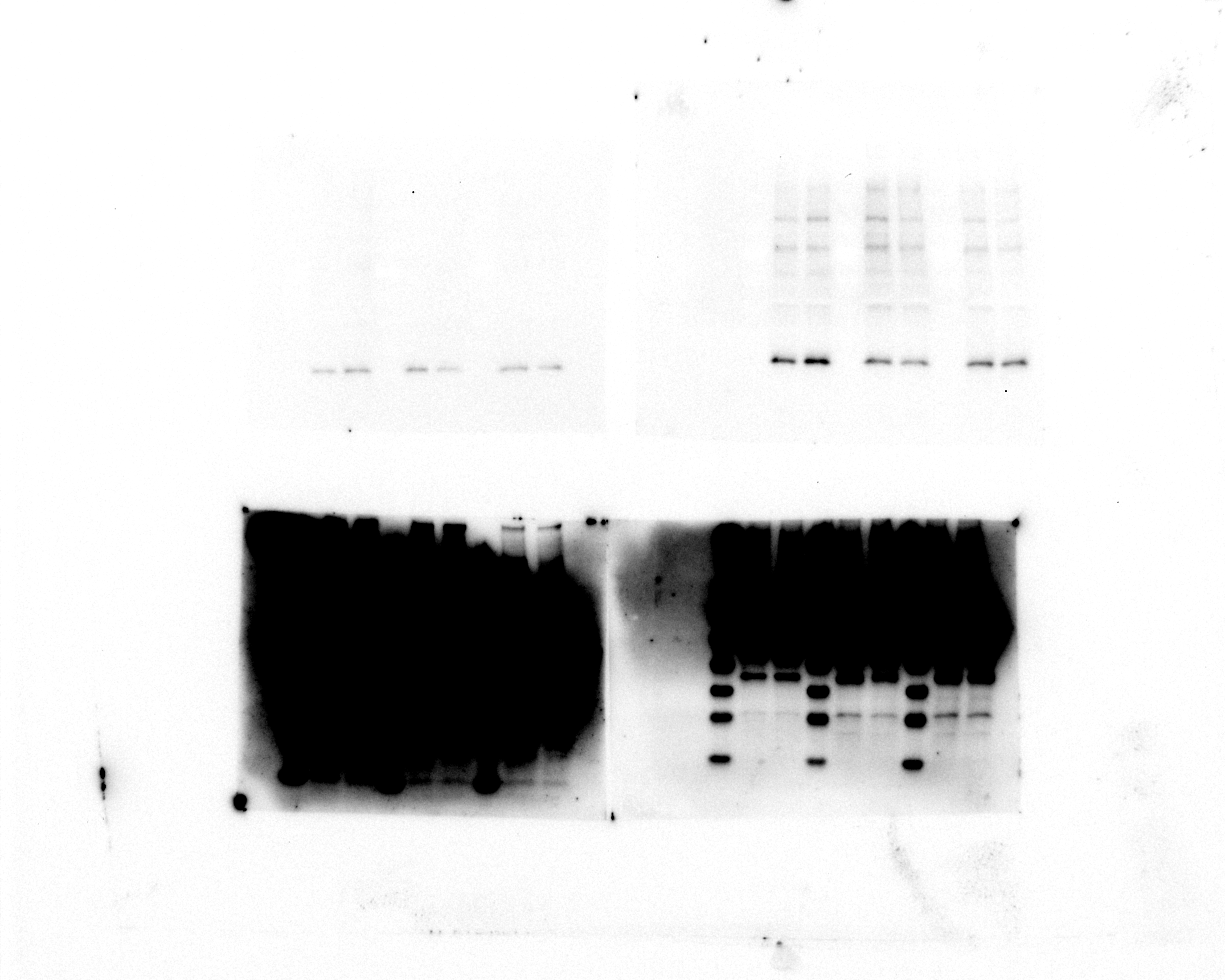

Supplement: Supplementary file 7 — Appendix and EV Figures Source Data [file 44319_2024_95_MOESM7_ESM.zip › Appendix_S4_SD/S4A source data SUM159 panel/Individual files S4A/fadd_top_right.tif]

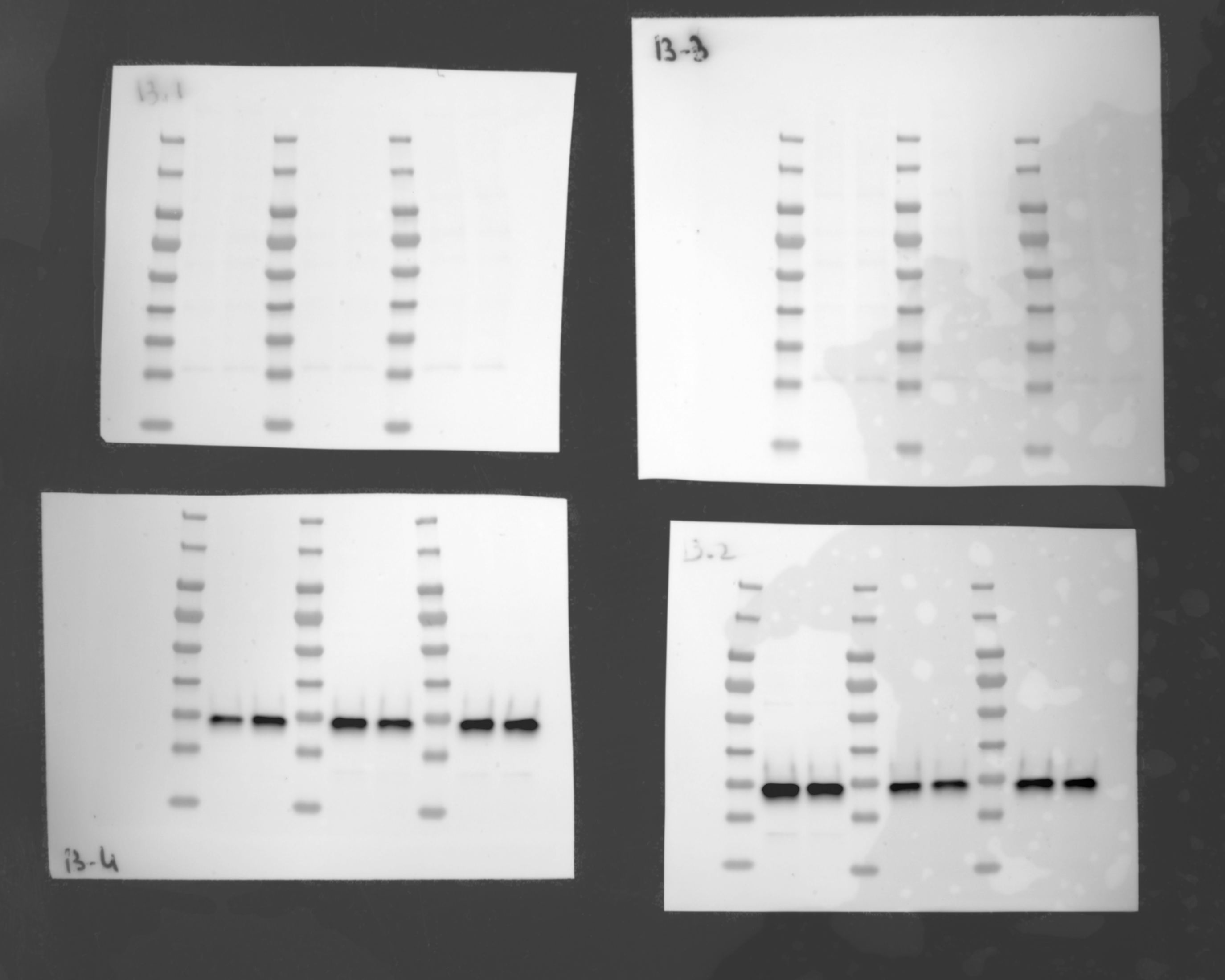

Supplement: Supplementary file 7 — Appendix and EV Figures Source Data [file 44319_2024_95_MOESM7_ESM.zip › Appendix_S4_SD/S4A source data SUM159 panel/Individual files S4A/puma caspase3-mark.tif]

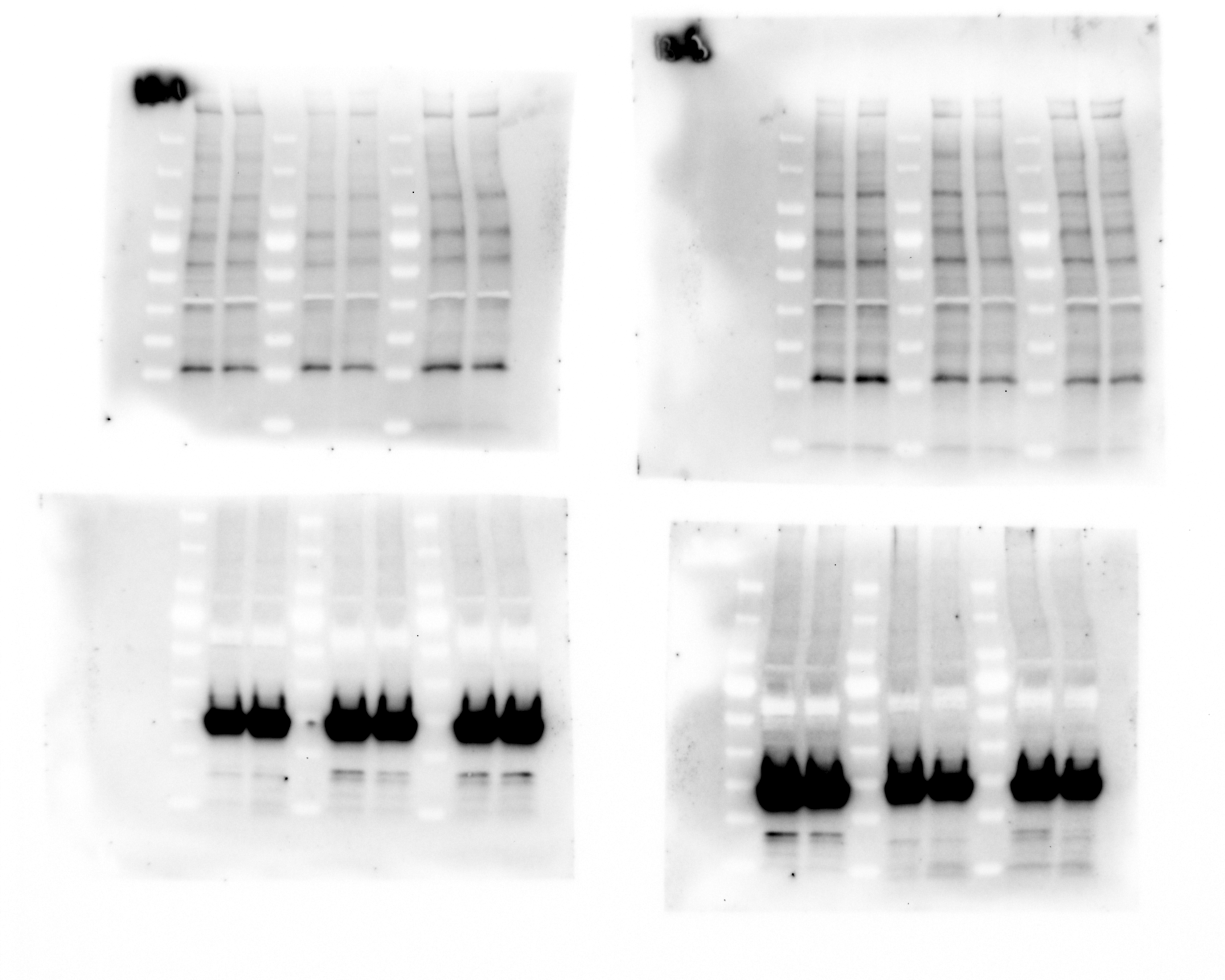

Supplement: Supplementary file 7 — Appendix and EV Figures Source Data [file 44319_2024_95_MOESM7_ESM.zip › Appendix_S4_SD/S4A source data SUM159 panel/Individual files S4A/puma top left.tif]

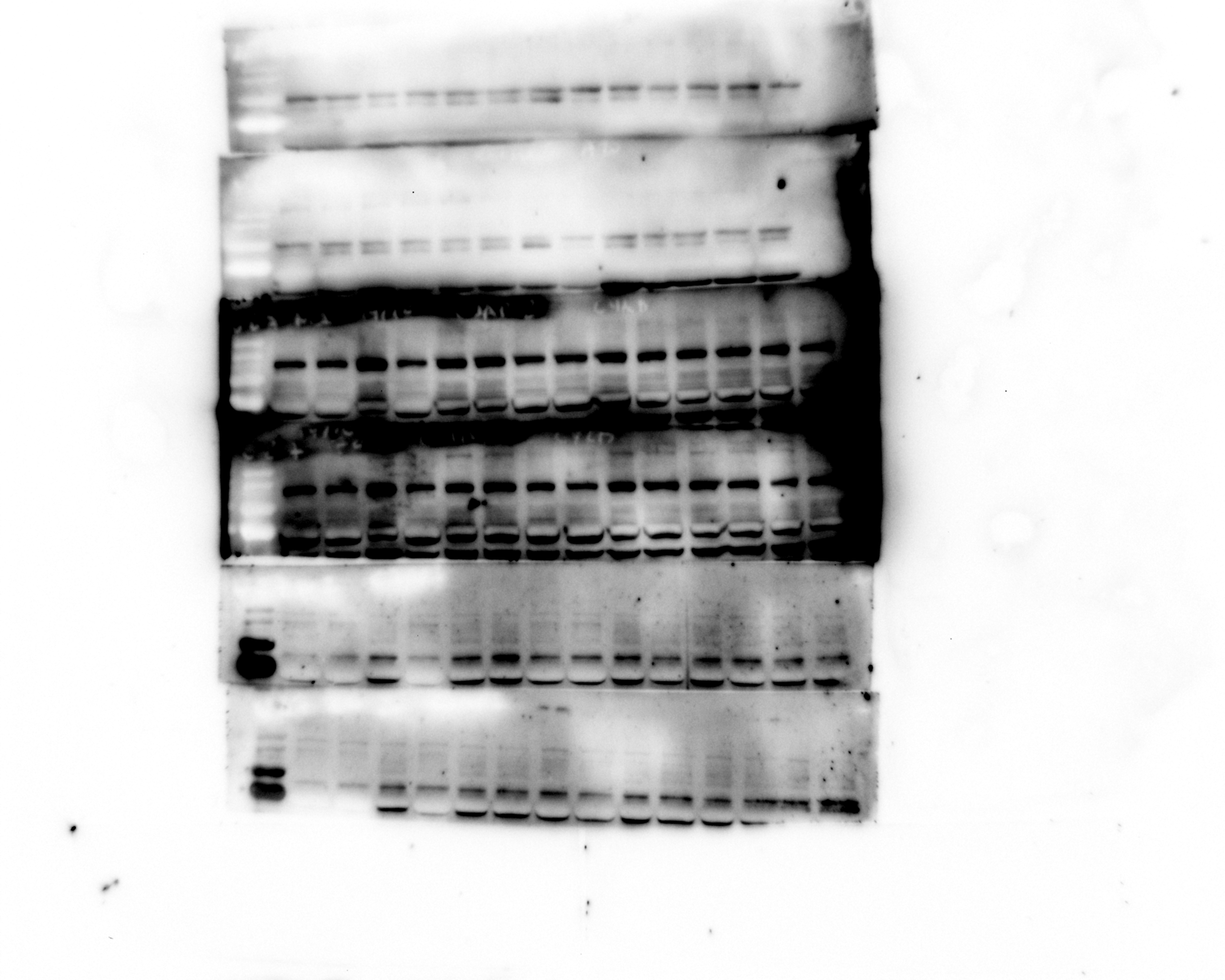

Supplement: Supplementary file 7 — Appendix and EV Figures Source Data [file 44319_2024_95_MOESM7_ESM.zip › Appendix_S4_SD/S4B source data/S4B individual files/A20_top.tif]

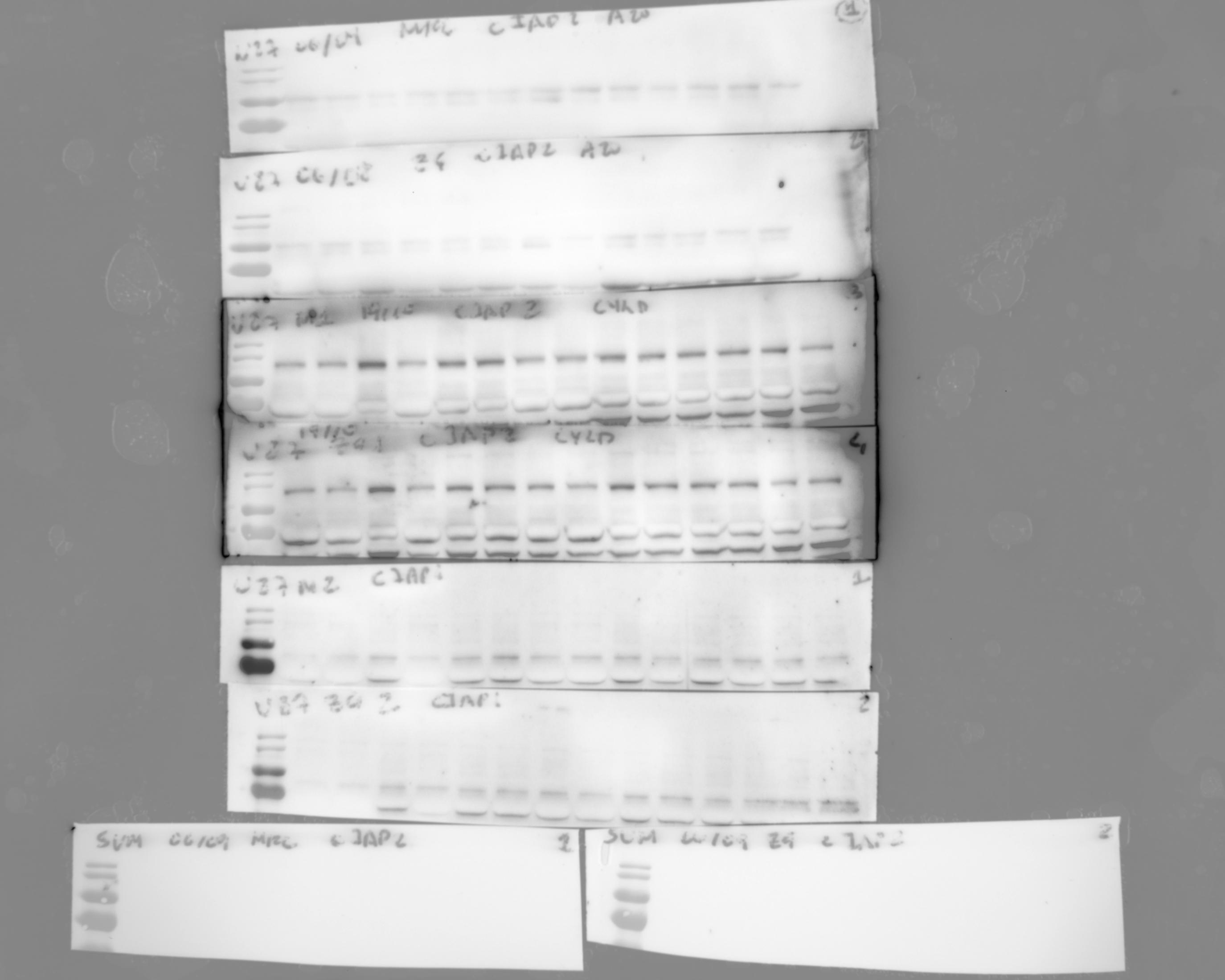

Supplement: Supplementary file 7 — Appendix and EV Figures Source Data [file 44319_2024_95_MOESM7_ESM.zip › Appendix_S4_SD/S4B source data/S4B individual files/A20top-mark.tif]

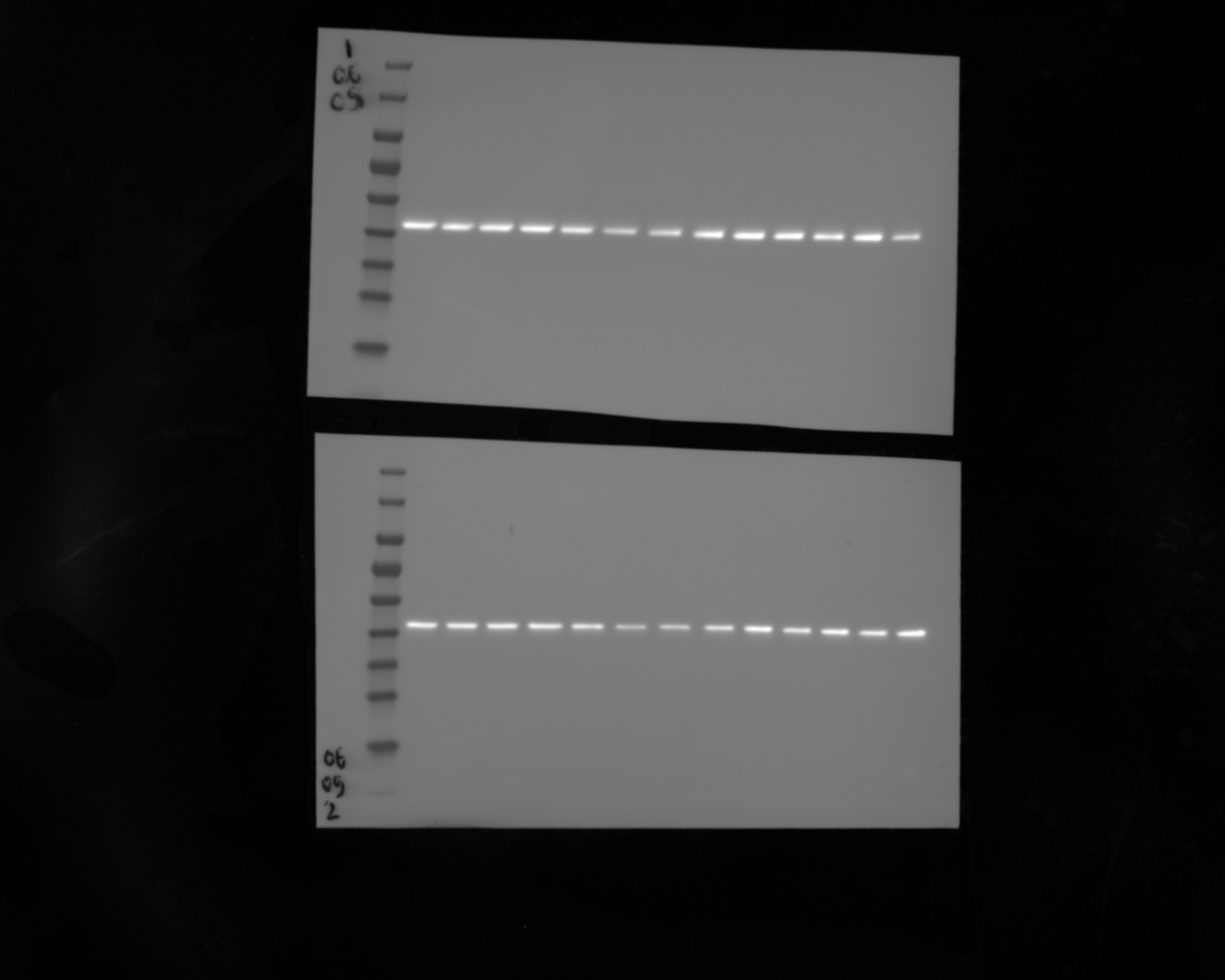

Supplement: Supplementary file 7 — Appendix and EV Figures Source Data [file 44319_2024_95_MOESM7_ESM.zip › Appendix_S4_SD/S4B source data/S4B individual files/actin-1-mark.tif]

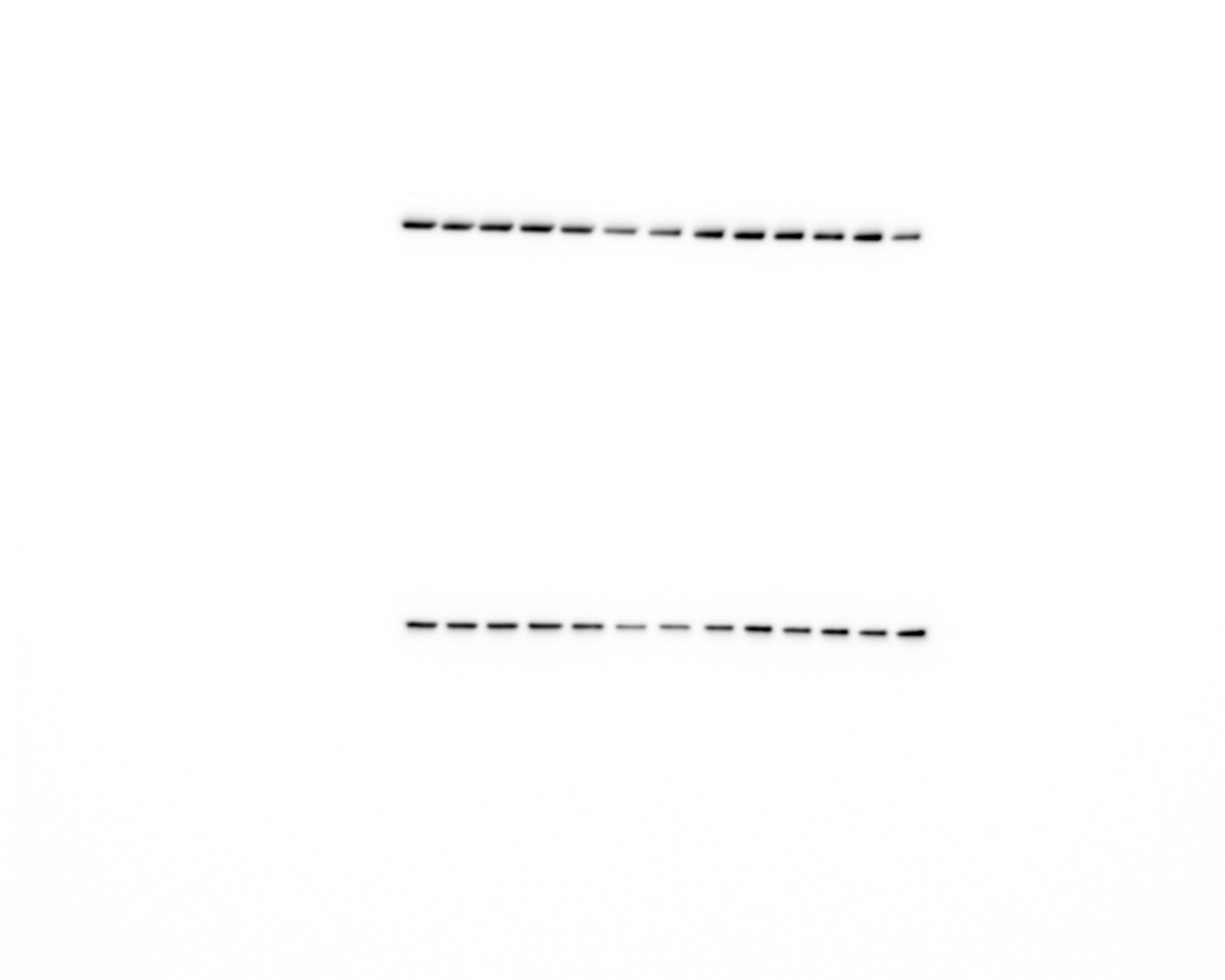

Supplement: Supplementary file 7 — Appendix and EV Figures Source Data [file 44319_2024_95_MOESM7_ESM.zip › Appendix_S4_SD/S4B source data/S4B individual files/actin-1.tif]

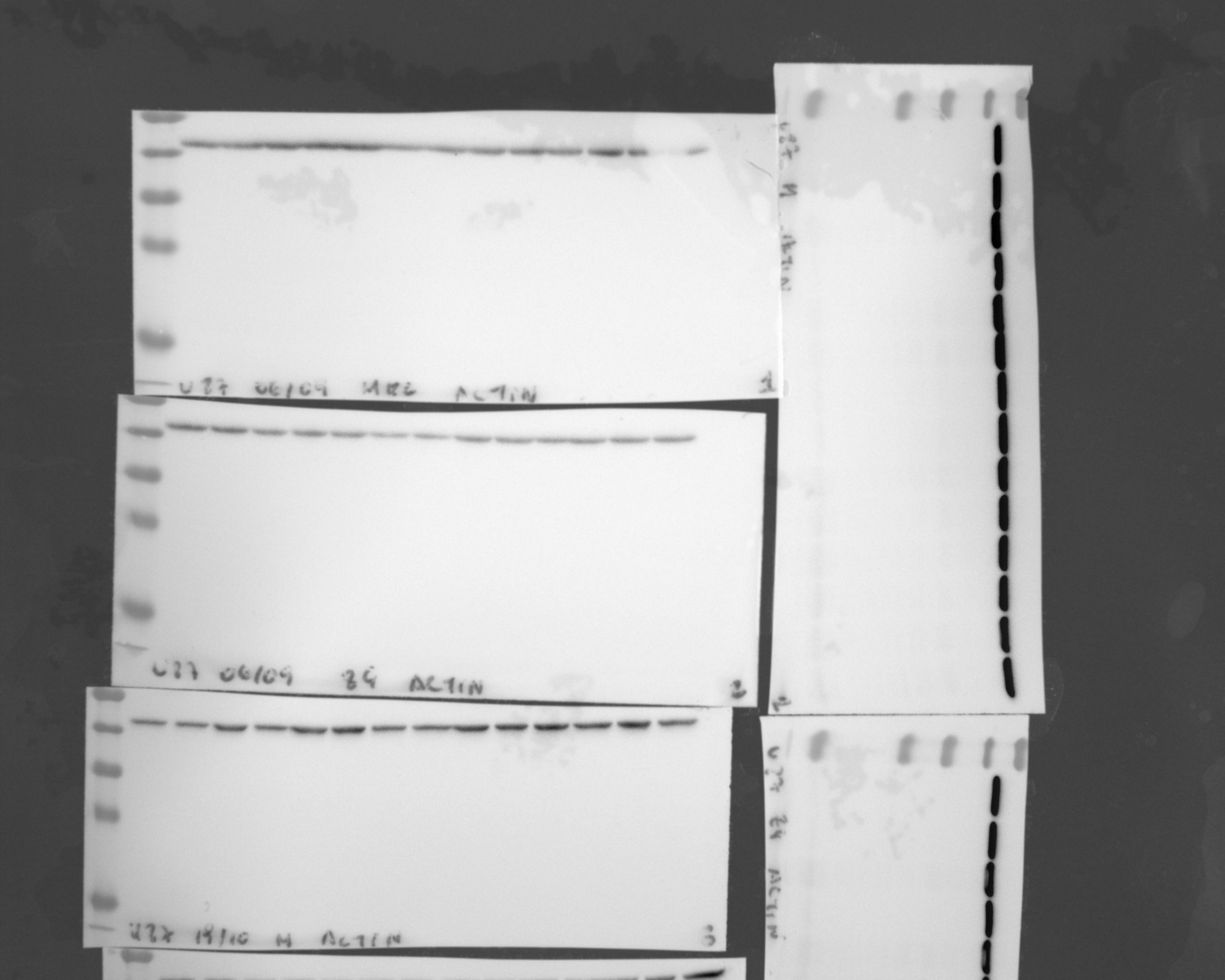

Supplement: Supplementary file 7 — Appendix and EV Figures Source Data [file 44319_2024_95_MOESM7_ESM.zip › Appendix_S4_SD/S4B source data/S4B individual files/ACTIN_top-mark.tif]

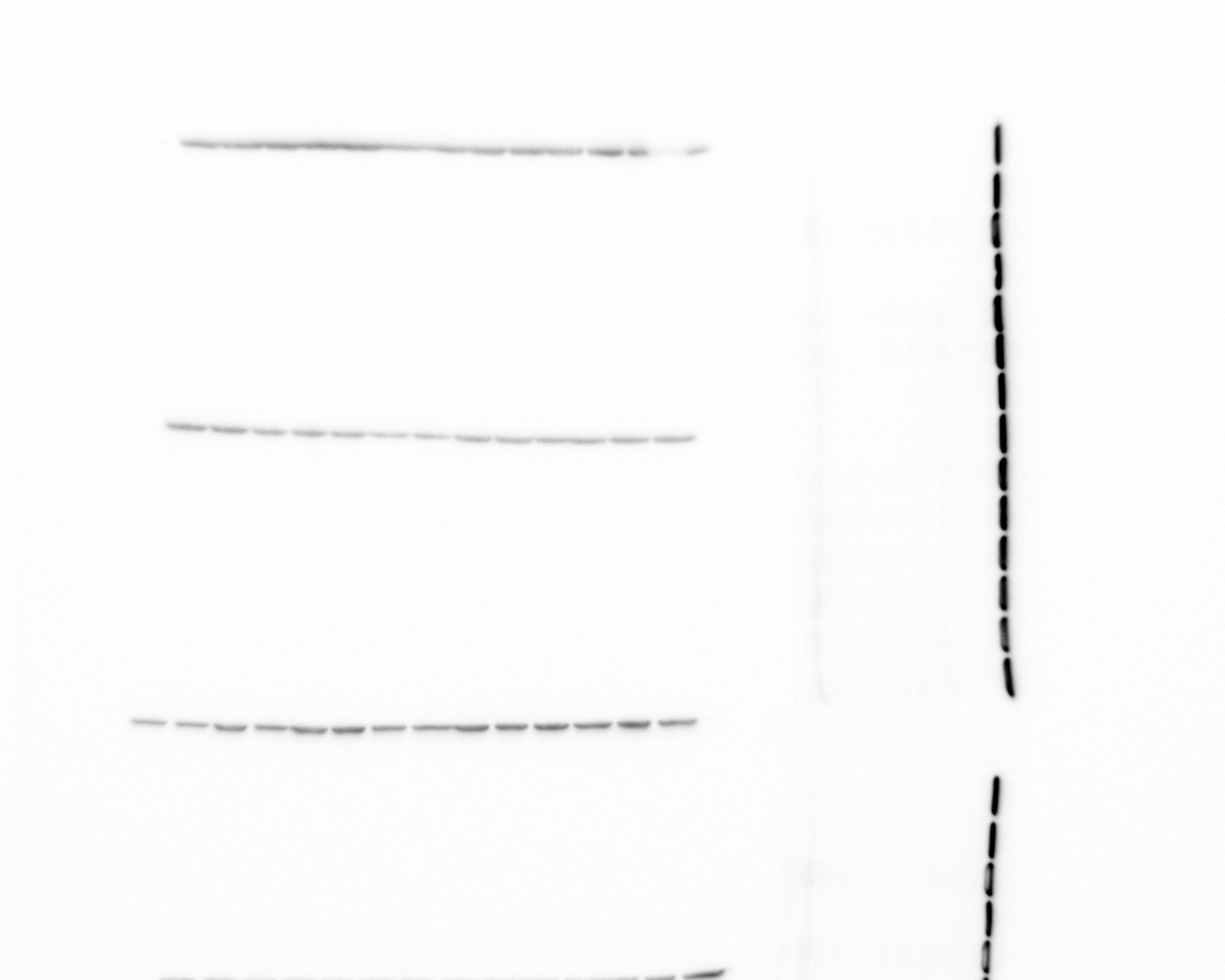

Supplement: Supplementary file 7 — Appendix and EV Figures Source Data [file 44319_2024_95_MOESM7_ESM.zip › Appendix_S4_SD/S4B source data/S4B individual files/ACTIN_top.tif]

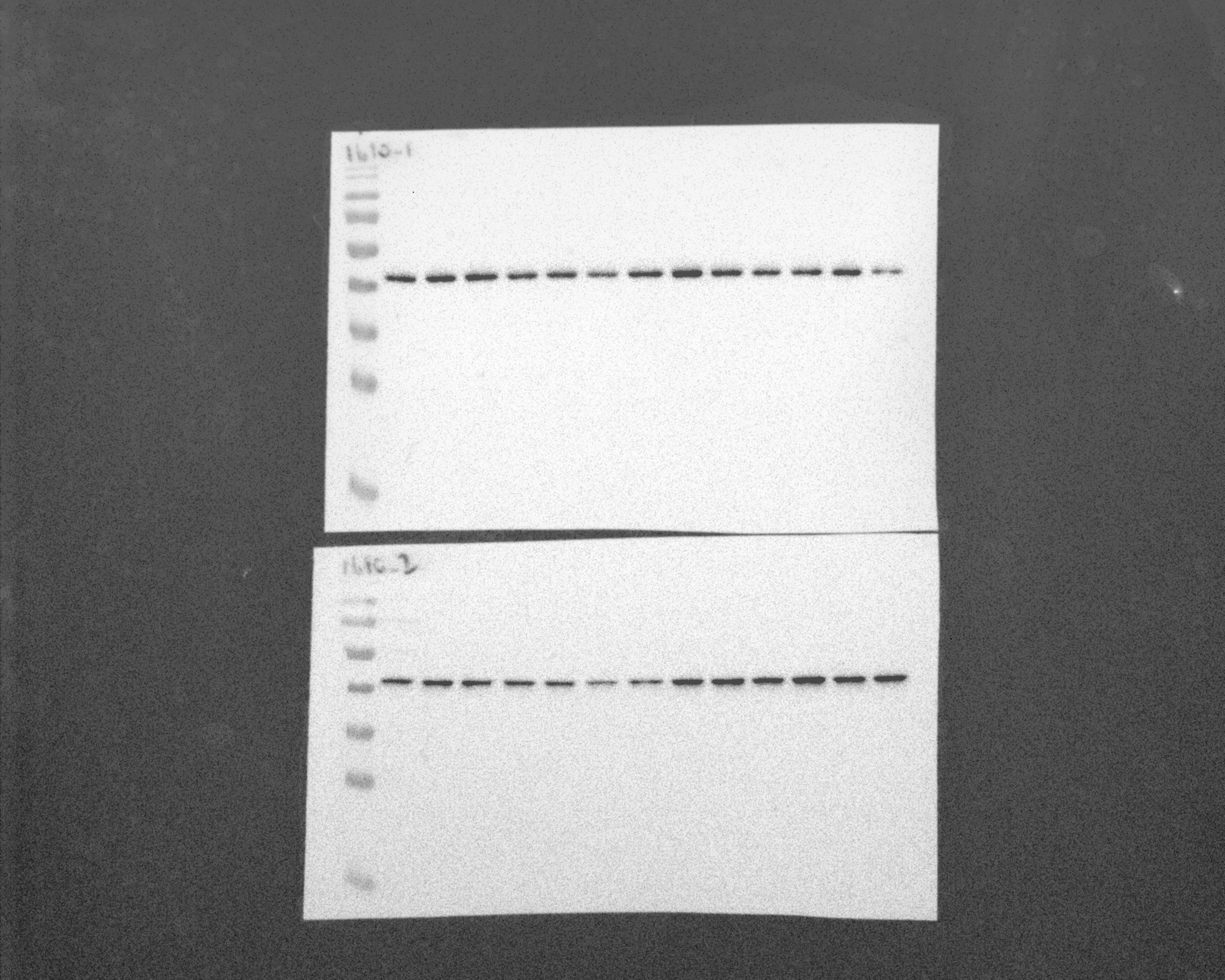

Supplement: Supplementary file 7 — Appendix and EV Figures Source Data [file 44319_2024_95_MOESM7_ESM.zip › Appendix_S4_SD/S4B source data/S4B individual files/actinreload-mark.tif]

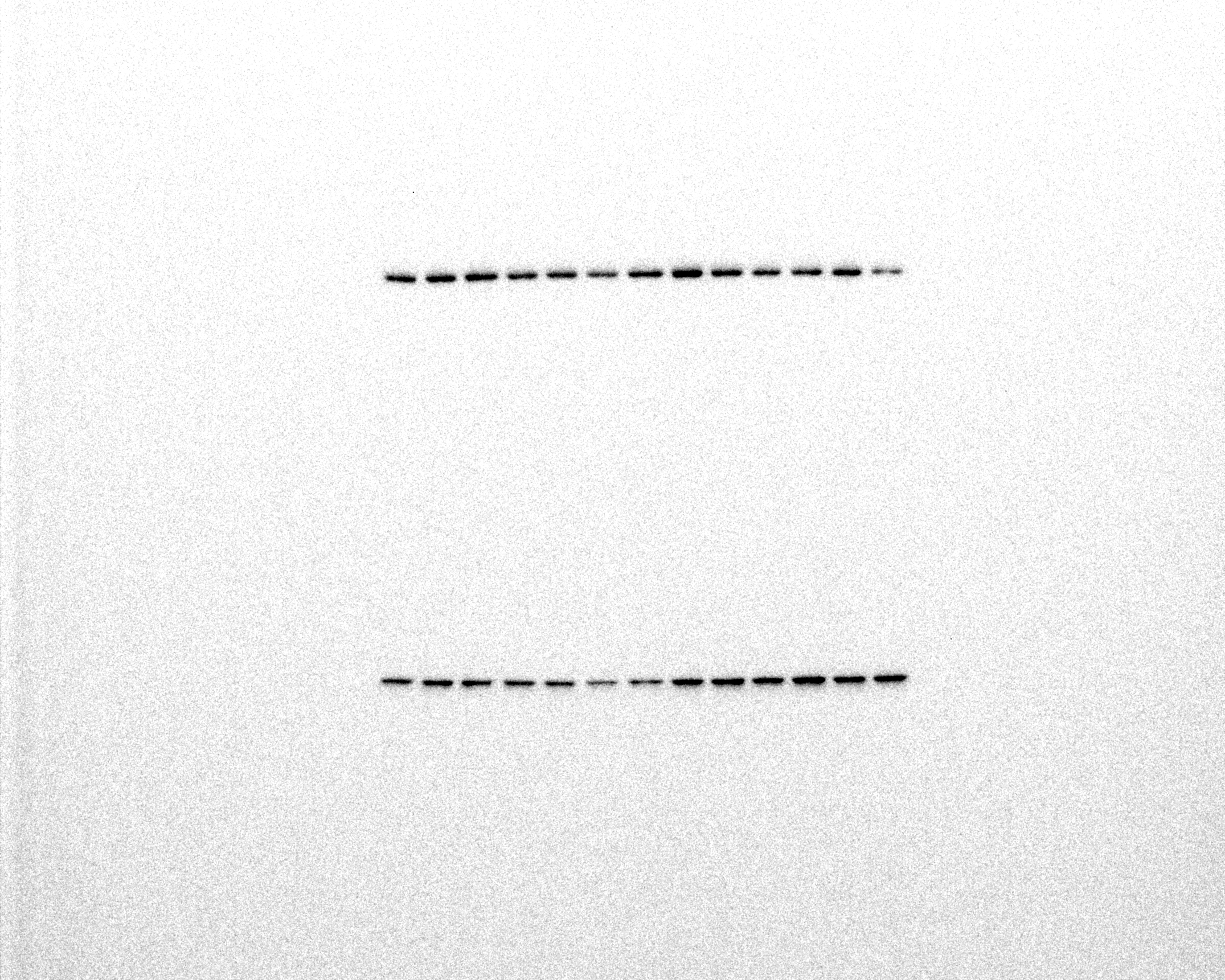

Supplement: Supplementary file 7 — Appendix and EV Figures Source Data [file 44319_2024_95_MOESM7_ESM.zip › Appendix_S4_SD/S4B source data/S4B individual files/actinreload.tif]

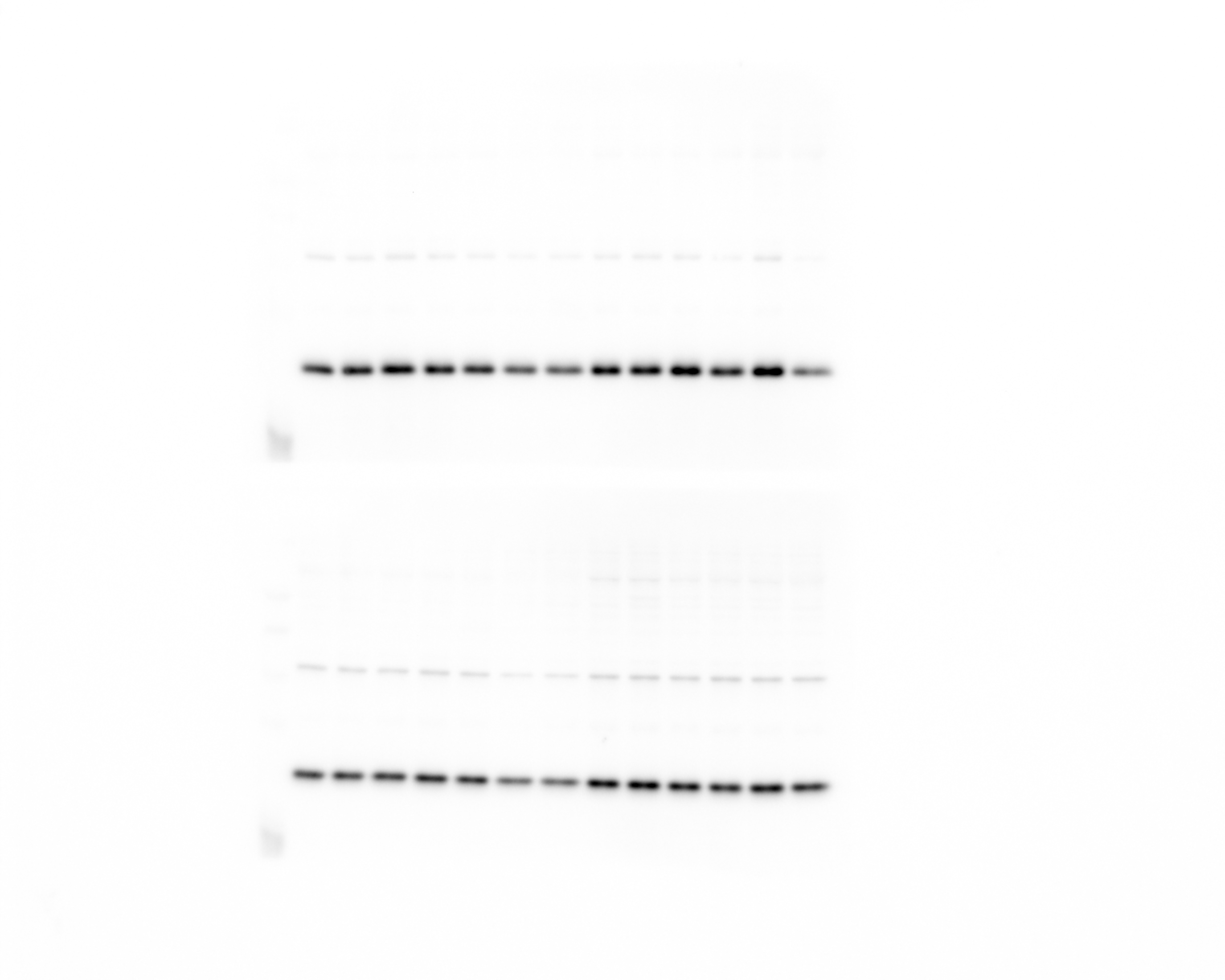

Supplement: Supplementary file 7 — Appendix and EV Figures Source Data [file 44319_2024_95_MOESM7_ESM.zip › Appendix_S4_SD/S4B source data/S4B individual files/bid-1.tif]

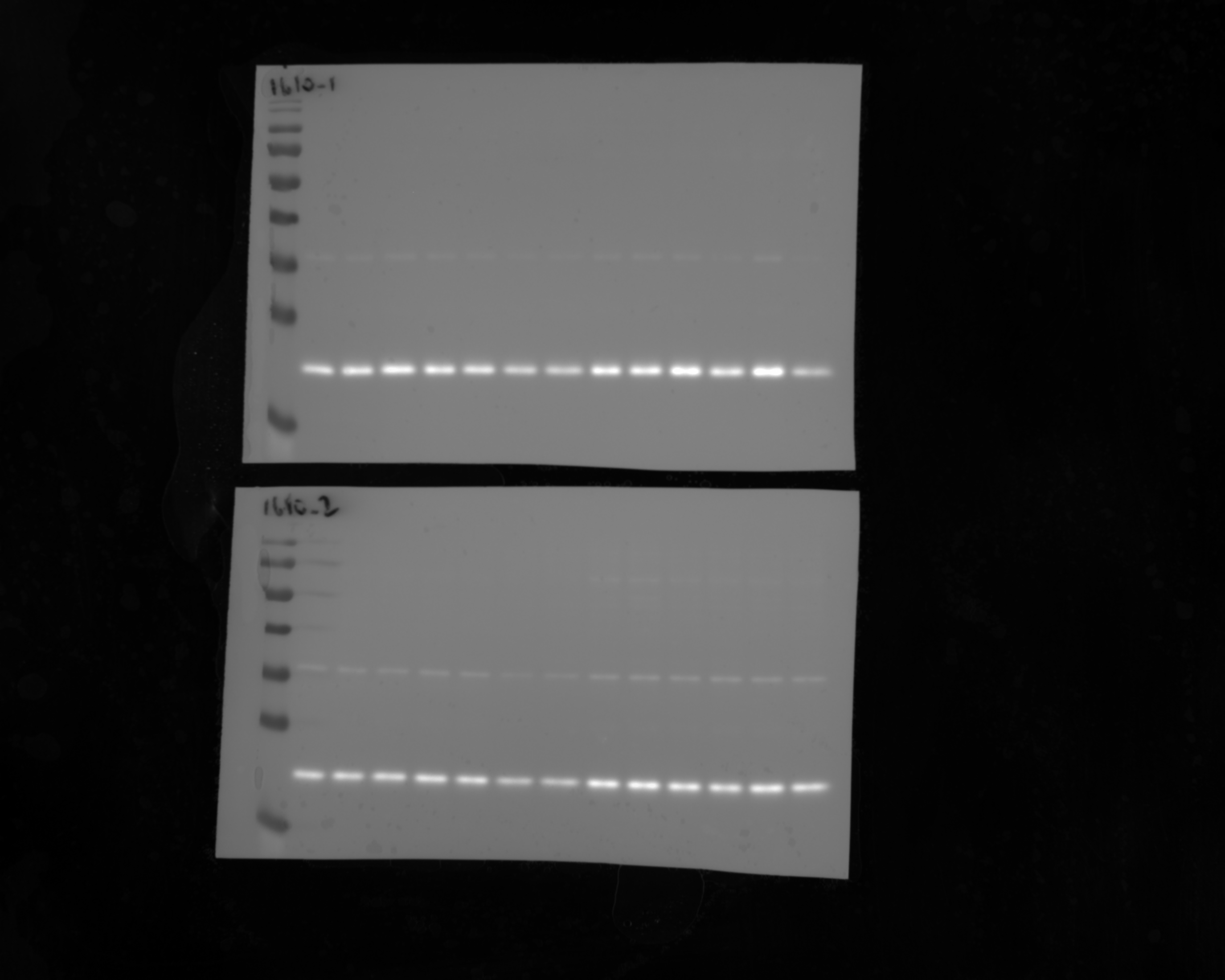

Supplement: Supplementary file 7 — Appendix and EV Figures Source Data [file 44319_2024_95_MOESM7_ESM.zip › Appendix_S4_SD/S4B source data/S4B individual files/bid-mark.tif]

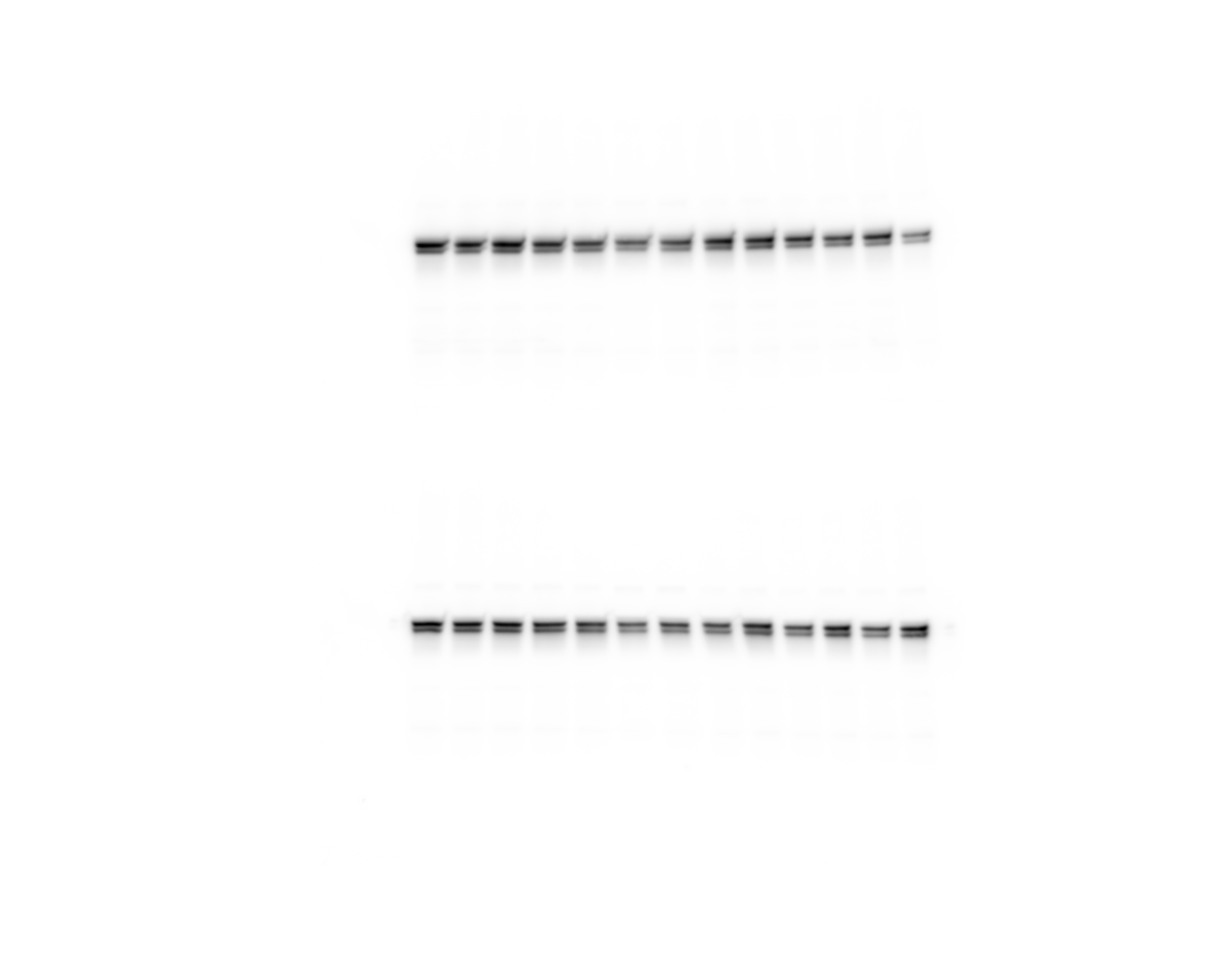

Supplement: Supplementary file 7 — Appendix and EV Figures Source Data [file 44319_2024_95_MOESM7_ESM.zip › Appendix_S4_SD/S4B source data/S4B individual files/caspase8-1.tif]

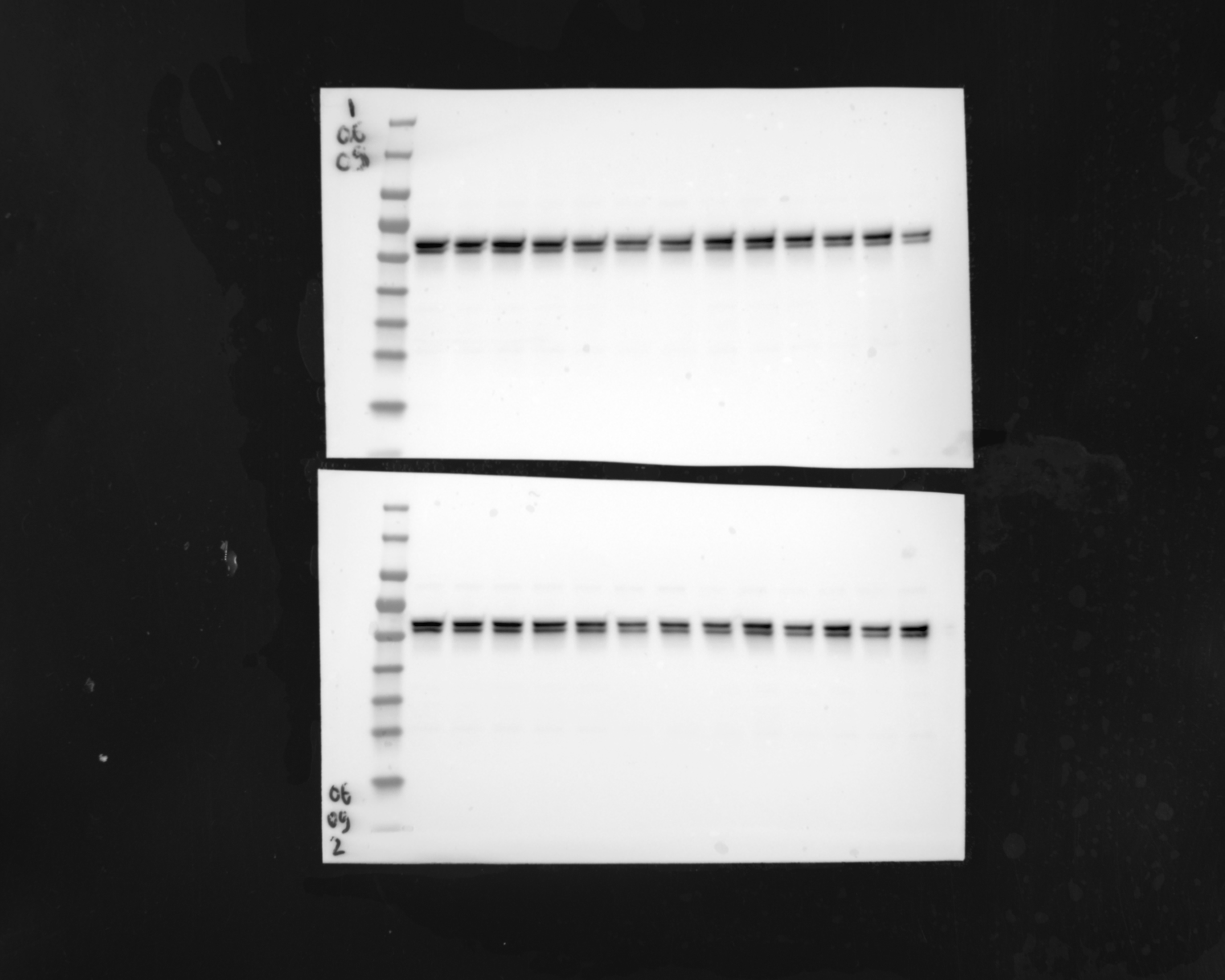

Supplement: Supplementary file 7 — Appendix and EV Figures Source Data [file 44319_2024_95_MOESM7_ESM.zip › Appendix_S4_SD/S4B source data/S4B individual files/caspase8-mark.tif]

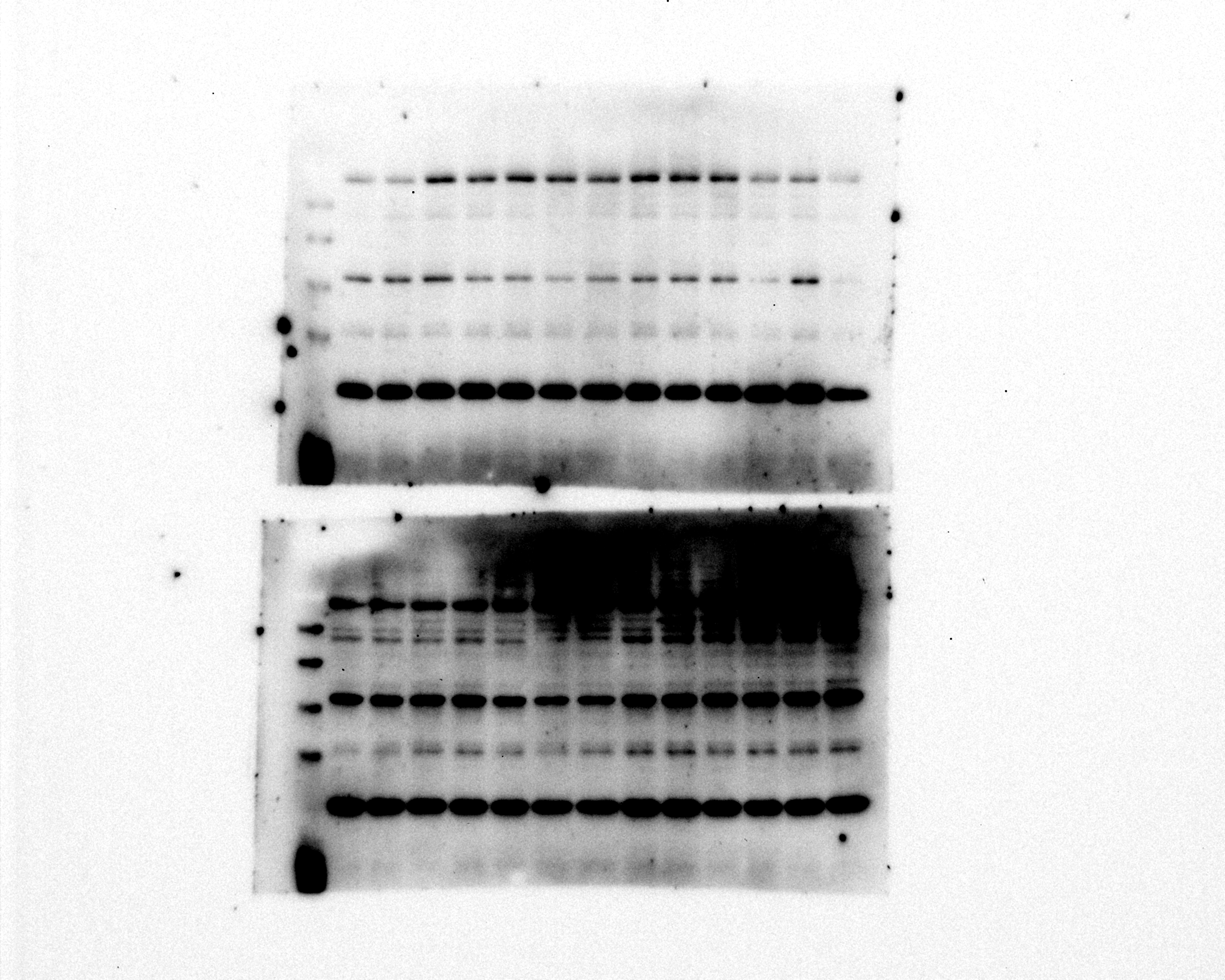

Supplement: Supplementary file 7 — Appendix and EV Figures Source Data [file 44319_2024_95_MOESM7_ESM.zip › Appendix_S4_SD/S4B source data/S4B individual files/ciap1-3.tif]

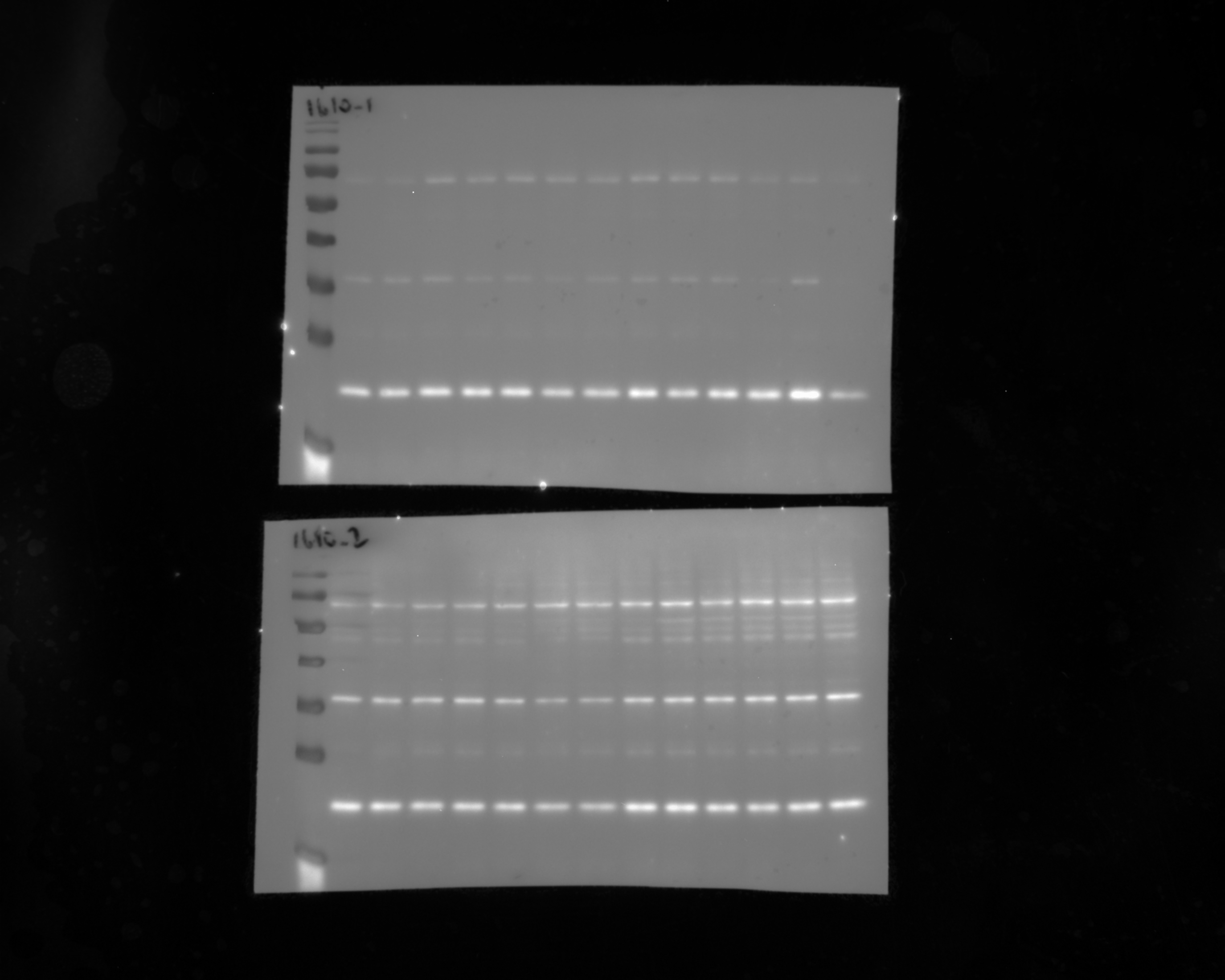

Supplement: Supplementary file 7 — Appendix and EV Figures Source Data [file 44319_2024_95_MOESM7_ESM.zip › Appendix_S4_SD/S4B source data/S4B individual files/ciap1-mark.tif]

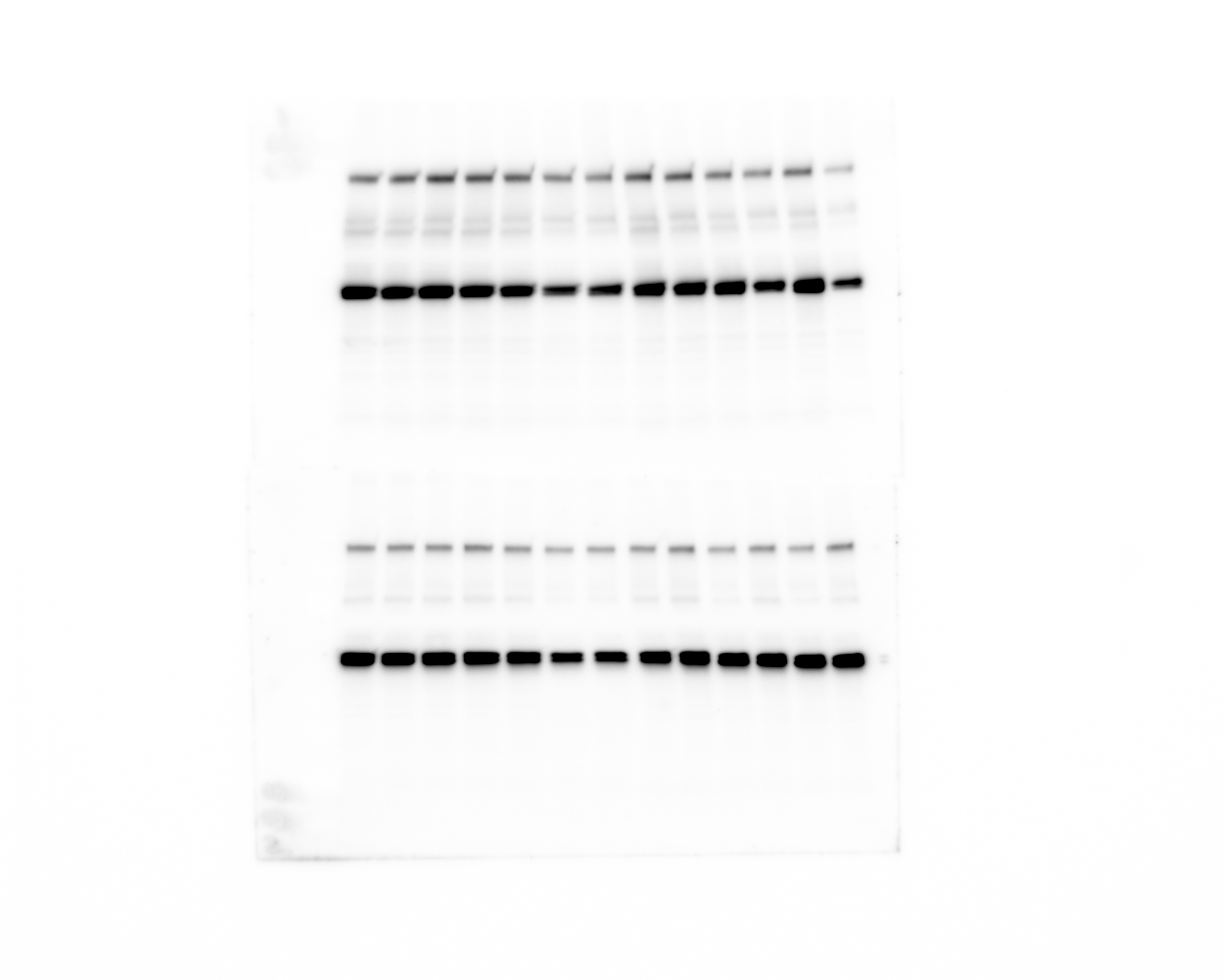

Supplement: Supplementary file 7 — Appendix and EV Figures Source Data [file 44319_2024_95_MOESM7_ESM.zip › Appendix_S4_SD/S4B source data/S4B individual files/CYLD-1.tif]

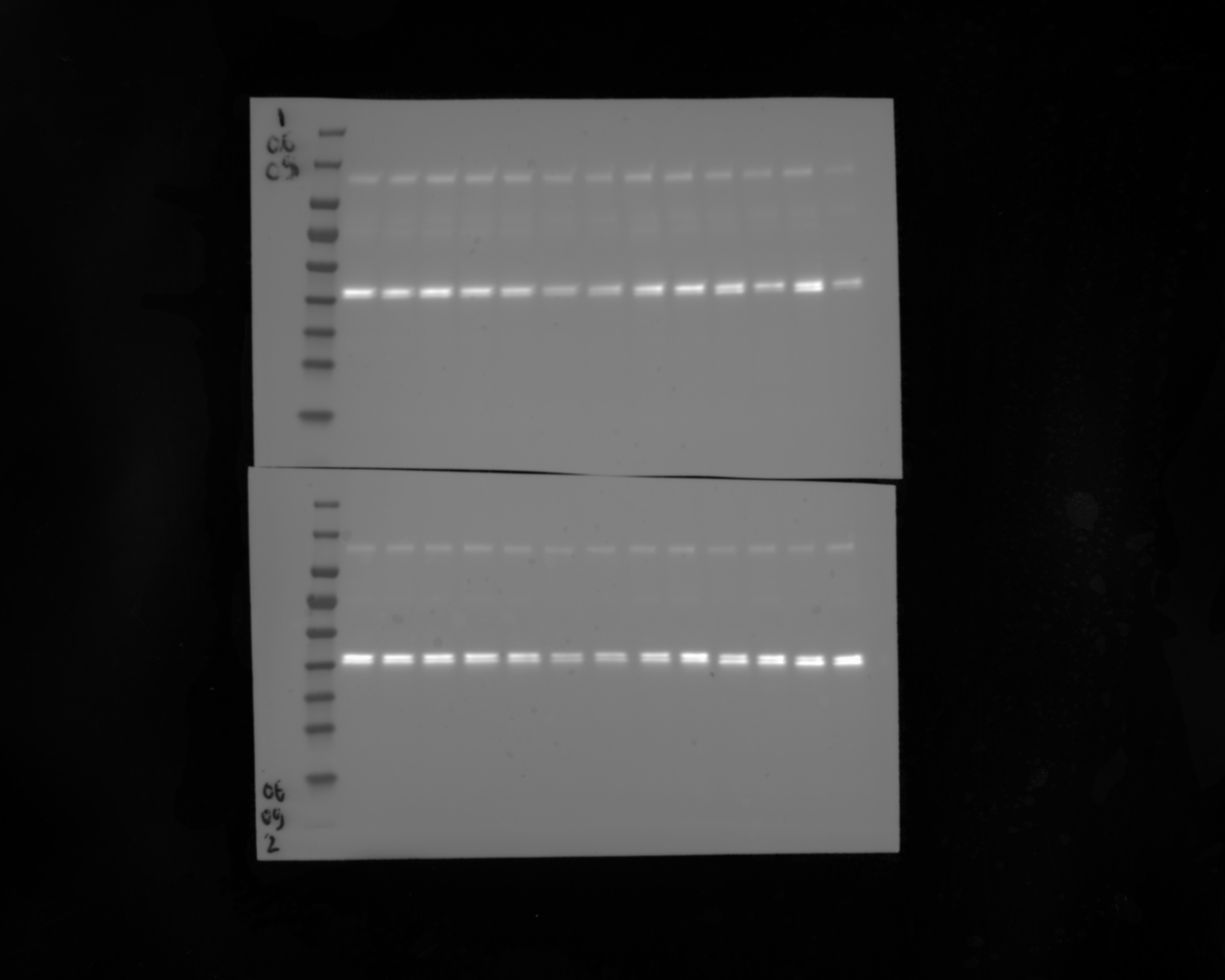

Supplement: Supplementary file 7 — Appendix and EV Figures Source Data [file 44319_2024_95_MOESM7_ESM.zip › Appendix_S4_SD/S4B source data/S4B individual files/cyld-mark.tif]

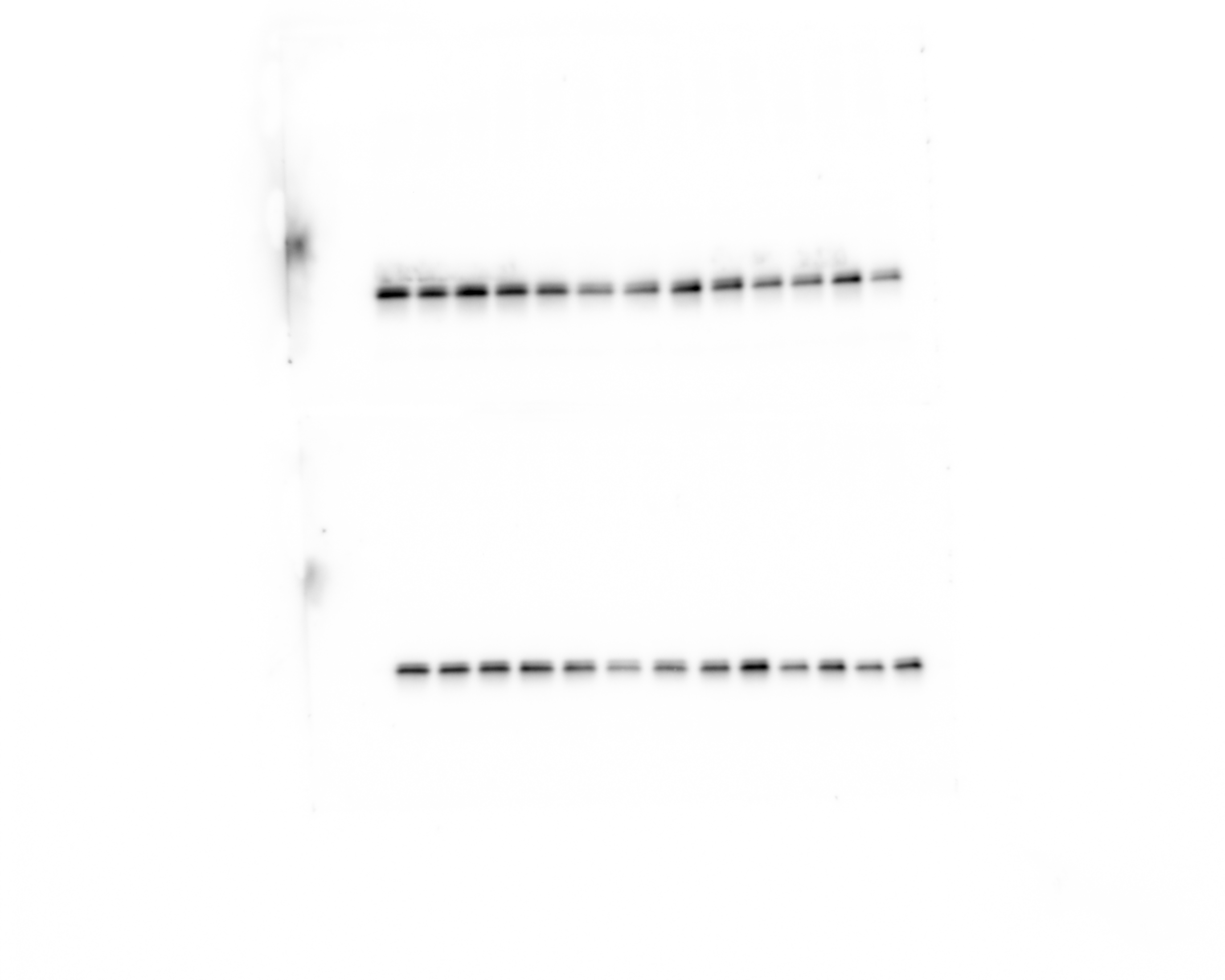

Supplement: Supplementary file 7 — Appendix and EV Figures Source Data [file 44319_2024_95_MOESM7_ESM.zip › Appendix_S4_SD/S4B source data/S4B individual files/FADD-1.tif]

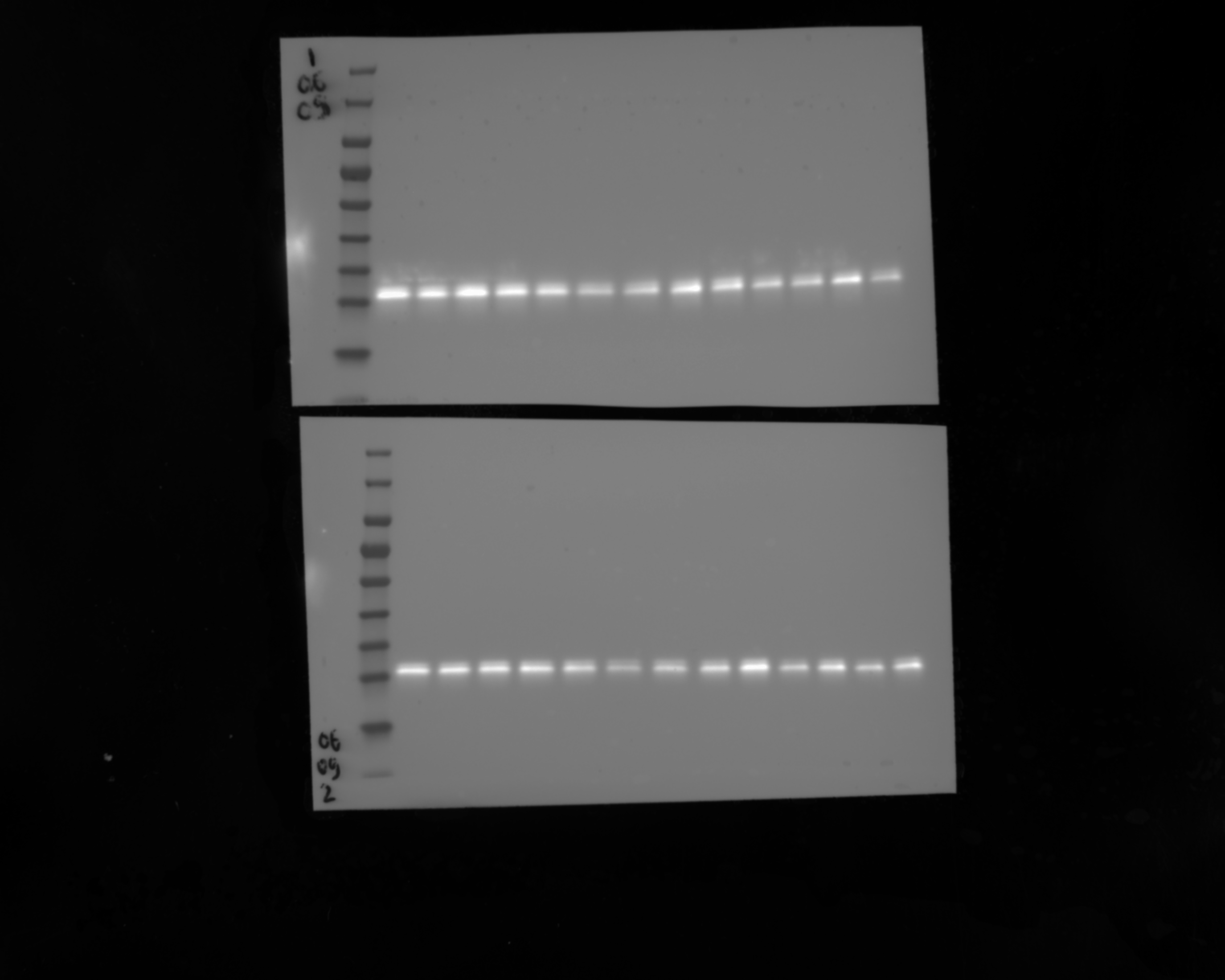

Supplement: Supplementary file 7 — Appendix and EV Figures Source Data [file 44319_2024_95_MOESM7_ESM.zip › Appendix_S4_SD/S4B source data/S4B individual files/FADD-mark.tif]

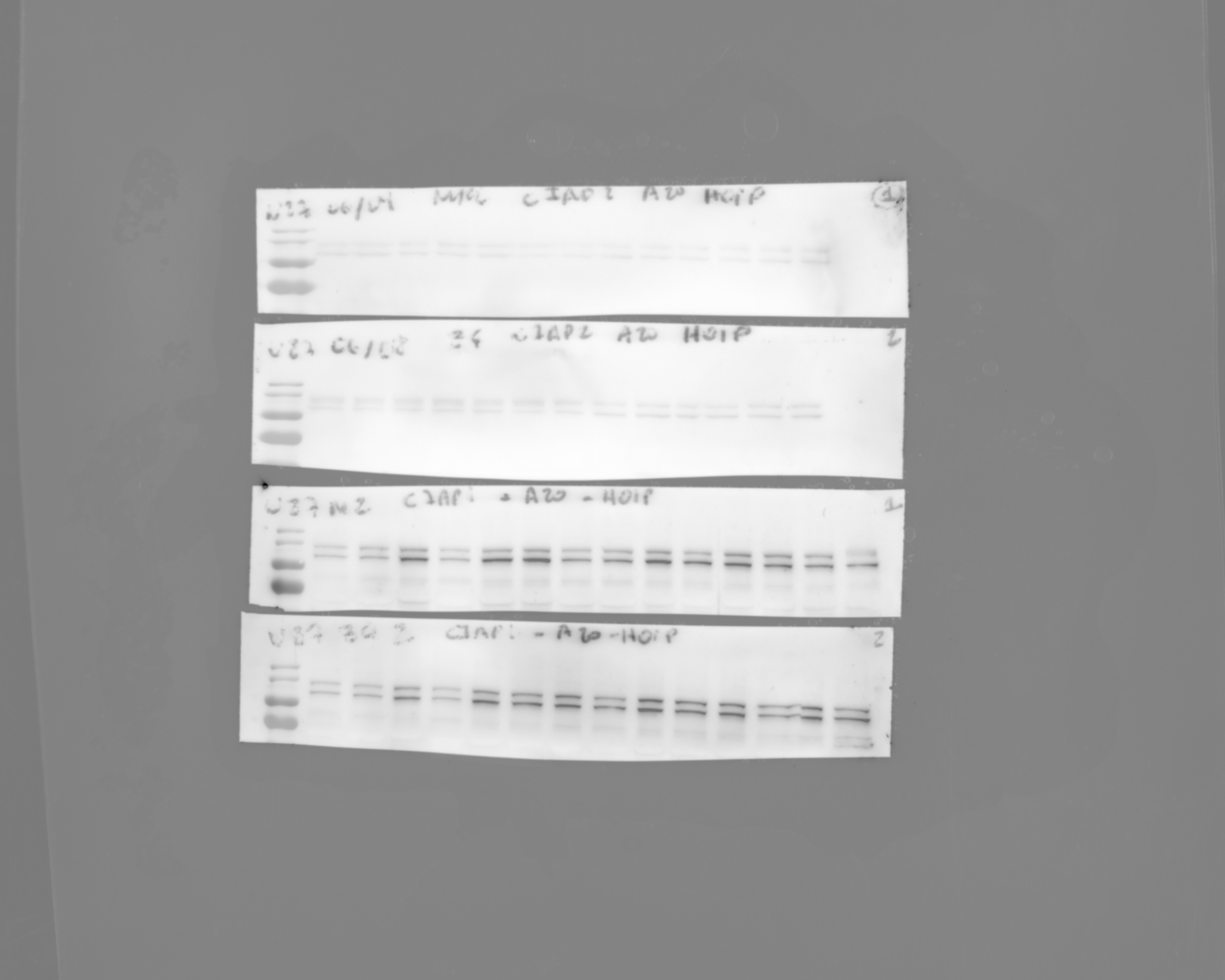

Supplement: Supplementary file 7 — Appendix and EV Figures Source Data [file 44319_2024_95_MOESM7_ESM.zip › Appendix_S4_SD/S4B source data/S4B individual files/HOIPtop-mark.tif]

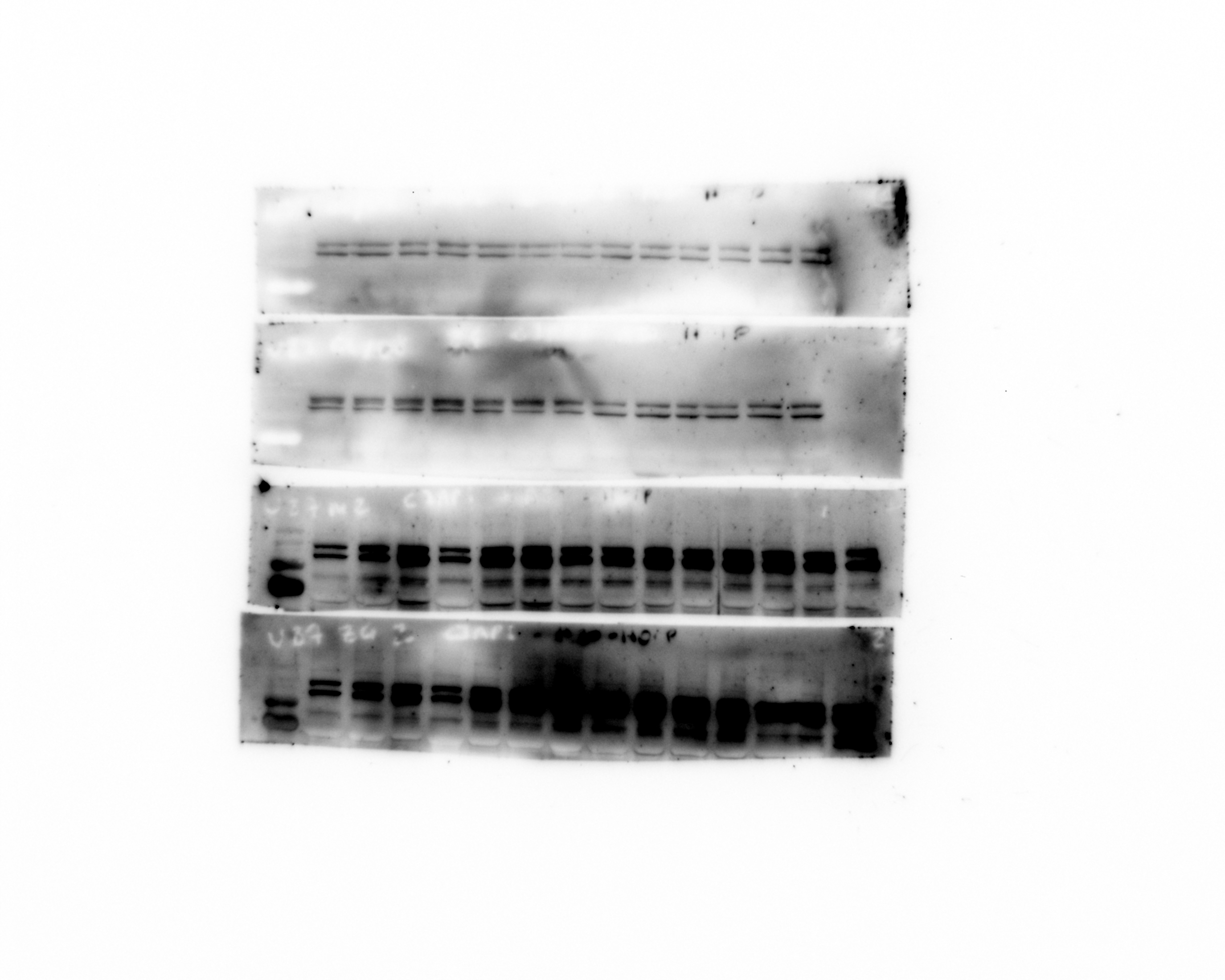

Supplement: Supplementary file 7 — Appendix and EV Figures Source Data [file 44319_2024_95_MOESM7_ESM.zip › Appendix_S4_SD/S4B source data/S4B individual files/HOIPtop.tif]

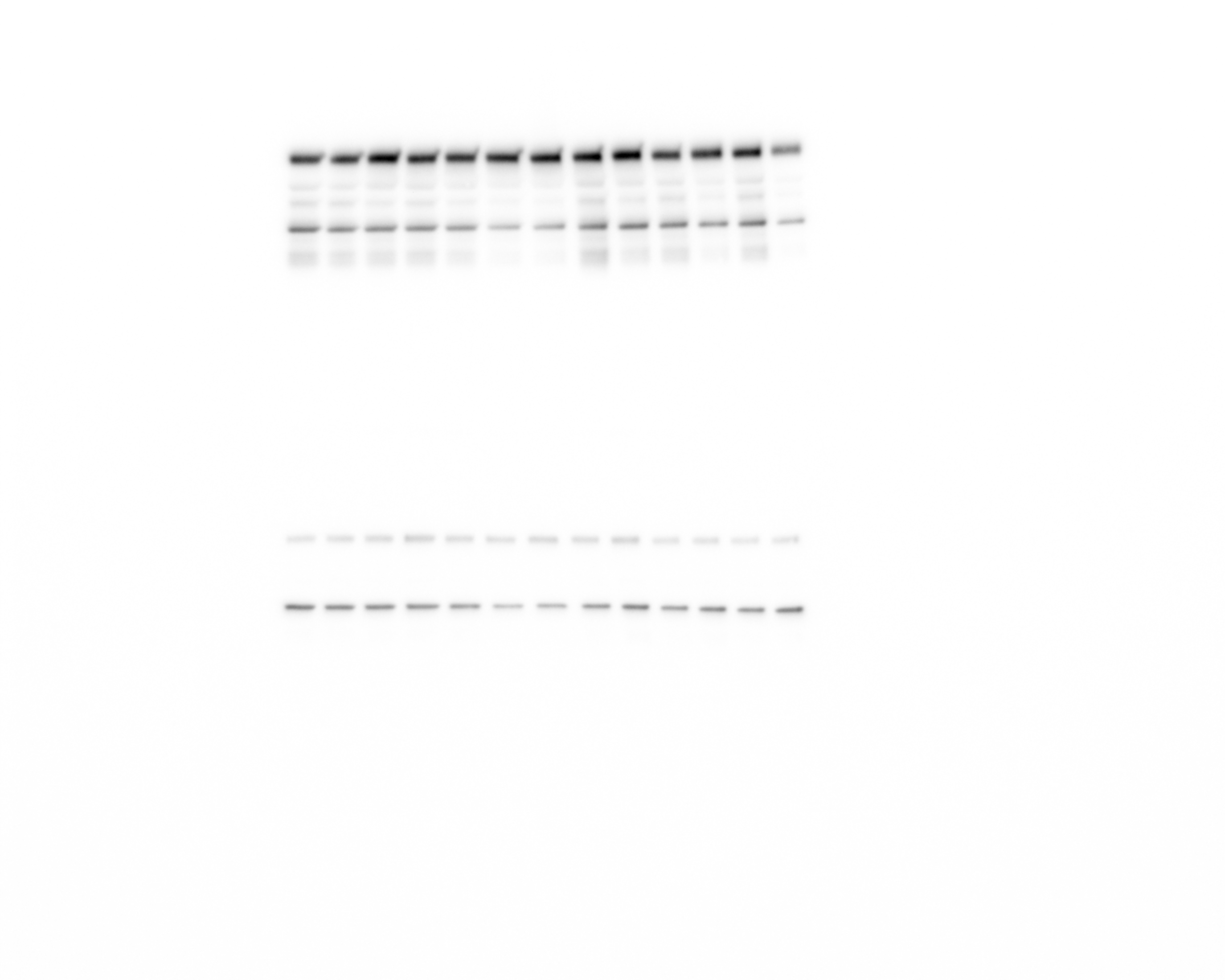

Supplement: Supplementary file 7 — Appendix and EV Figures Source Data [file 44319_2024_95_MOESM7_ESM.zip › Appendix_S4_SD/S4B source data/S4B individual files/RIPK1-1.tif]

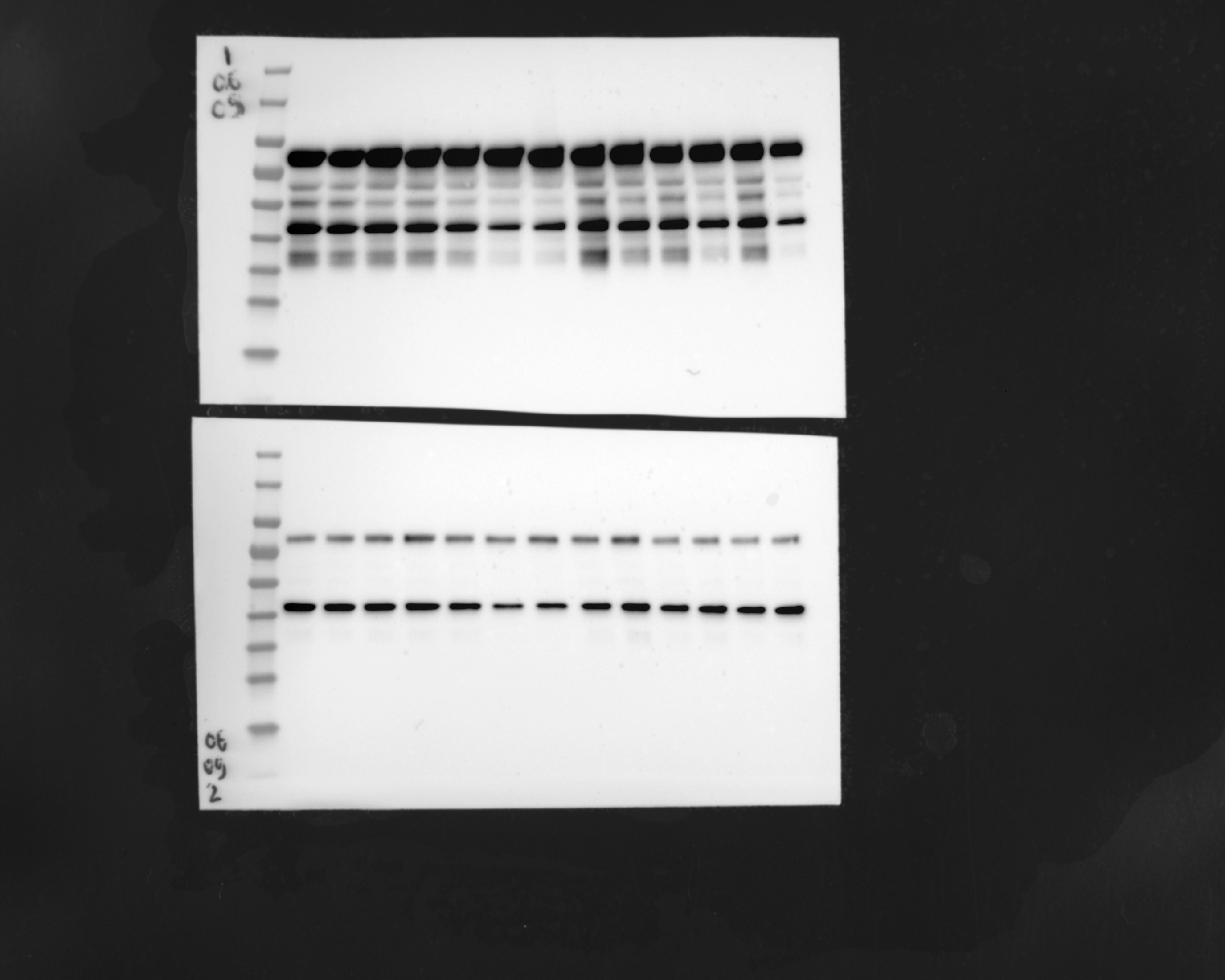

Supplement: Supplementary file 7 — Appendix and EV Figures Source Data [file 44319_2024_95_MOESM7_ESM.zip › Appendix_S4_SD/S4B source data/S4B individual files/RIPK1-mark.tif]
