# Supplementary material for: IRE1 RNase controls CD95-mediated cell death
Source: EMBO Rep. 2024 Feb 21;25(4):13. doi: 10.1038/s44319-024-00095-9 (PMC11014915; doi:10.1038/s44319-024-00095-9)
Supplement: Supplementary file 9 — Expanded View Figures [file 44319_2024_95_MOESM9_ESM.pdf]

Expanded View Figures

Figure EV1. IRE1 represses CD95 expression in GB cells upon ER stress.

(**A**) CD95 protein level was evaluated using western blot on lysates from the indicated cells. One representative experiment out of three independent experiments is presented. (**B**) U87 or SUM159 cells were pre-incubated for 1 h with 1 µg/mL of actinomycin D and further treated with 10 µM MKC-8866 for 1 h followed by 2 h treatment with 10 µM MG-132 as indicated. CD95 mRNA expression level, normalized to GAPDH, was expressed as fold of value obtained for control (actinomycin-only treated samples). Mean ± SEM, $n = 3$–4. Unpaired t-test (for comparing MG-132 and MG-132 + MKC-treated group), ****$p = 0.0003$. (**C**,**D**) U87 or SUM159 cells were pre-incubated for 1 h with 1 µg/mL of actinomycin D and further treated with 10 µM MKC-8866 or 10 µM Z4 for 1 h followed by 2 h treatment with 1µg/mL tunicamycin as indicated. CD95 mRNA expression level, normalized to GAPDH, was expressed as fold of value obtained for control (actinomycin-only treated samples). Mean ± SEM, $n = 3$–4. Unpaired t-test (for comparing TM and TM + MKC groups for (**C**) or TM and TM + Z4 groups for (**D**)), (**C**) *$p = 0.0461$ for U87, *$p = 0.0437$ for SUM159, (**D**) *$p = 0.0241$ for U87, (ns, $p = 0.0538$ for SUM159).

© The Author(s)

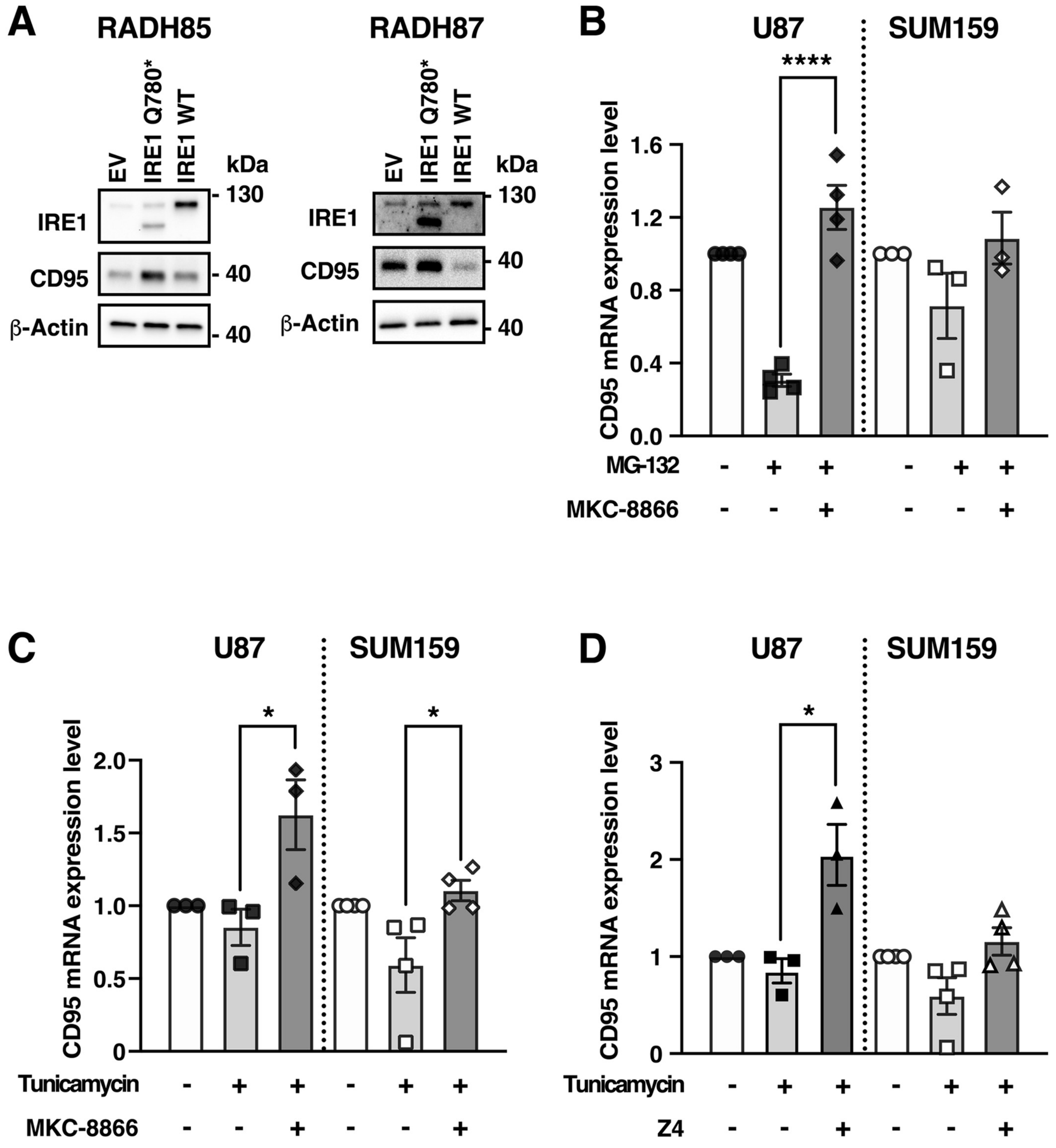
A
RADH85
EV
IRE1 Q780*
IRE1 WT
kDa
IRE1
- 130
CD95
- 40
β-Actin
- 40
RADH87
EV
IRE1 Q780*
IRE1 WT
kDa
IRE1
130
CD95
40
β-Actin
40
B
U87
SUM159
****
CD95 mRNA expression level
1.6
1.2
0.8
0.4
0.0
MG-132 - + + - + +
MKC-8866 - - + - - +
C
U87
SUM159
*
*
CD95 mRNA expression level
2.0
1.5
1.0
0.5
0
Tunicamycin - + + - + +
MKC-8866 - - + - - +
D
U87
SUM159
*
CD95 mRNA expression level
3
2
1
0
Tunicamycin - + + - + +
Z4 - - + - - +

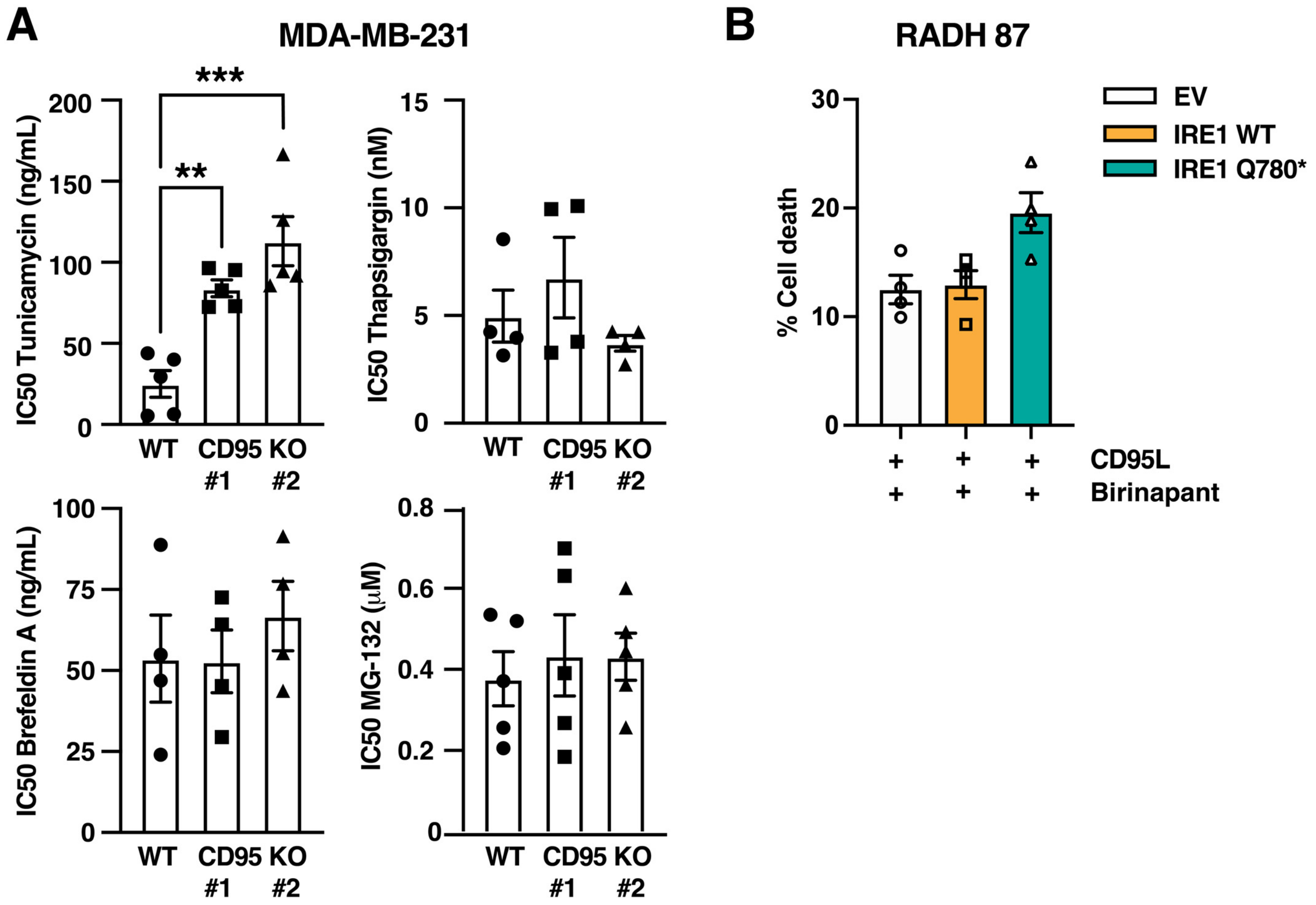

Figure EV2. CD95 is not a universal determinant of ER-stress induced cell death whilst IRE1 RNase activity limits CD95L-induced cell death.

(**A**) MDA-MB-231 WT or CD95 KO clones were treated for 48 h with the indicated ER stress inducers. Viability was determined using an MTT assay and relative IC50 calculated for each independent experiment (see also Appendix Fig. 2B). ***p* = 0.044, ****p* = 0.0002, one-way ANOVA with Tukey multiple comparison correction.
(**B**) RADH87 control (EV), stably expressing IRE1Q780* or IRE1WT were pre-treated with 200 nM (2X) of Birinapant for 1 h prior to addition of 1 μg/mL CD95L for 24 h. % of cell death was defined as the % of Cytotox red-positive cells as detected by the Incucyte. Mean ± SEM of three independent experiments.

 © The Author(s)

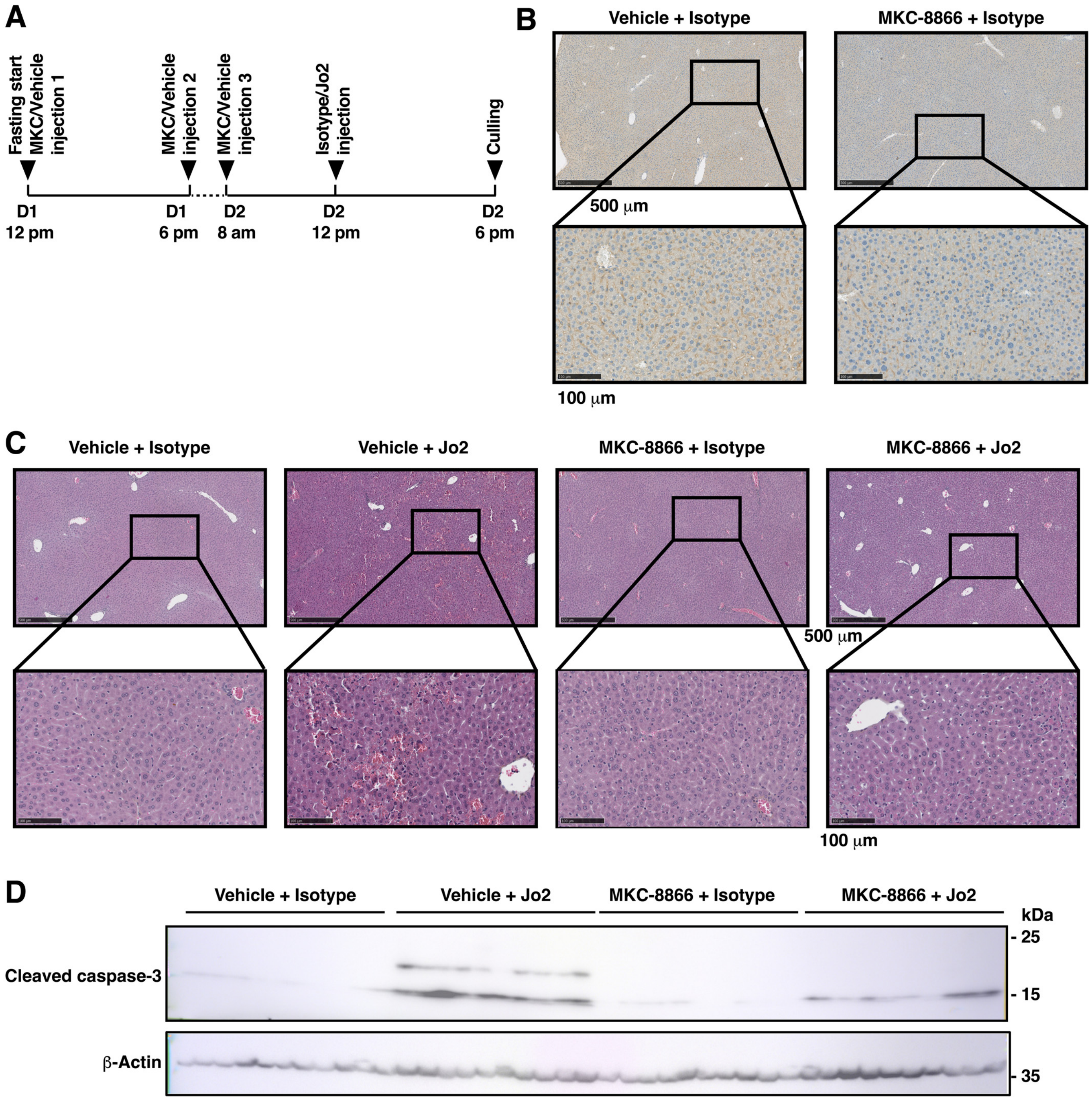

Figure EV3. IRE1 RNase inhibition limits hepatic CD95 expression and CD95-mediated cell death in mice.

(**A**) Timeline of the second in vivo experiment. 40 mice were divided in two groups of 20 and were repeatedly injected with either vehicle or MKC-8866 as indicated. On day 2 at 12 pm, each of this initial groups were further divided in two groups of 10 mice which were injected with either an anti-CD95 antibody or with an isotype control as indicated. (**B**) CD95 expression was evaluated by IHC in mice injected with vehicle or MKC-8866 and the isotype control antibody. One representative image is shown for each of these two groups. (**C**) HES staining was performed on liver tissue sections from mice of each of the four groups described in (**A**). One representative image is shown for each of these groups. (**D**) Western blot analysis of liver lysates from mice treated as indicated.

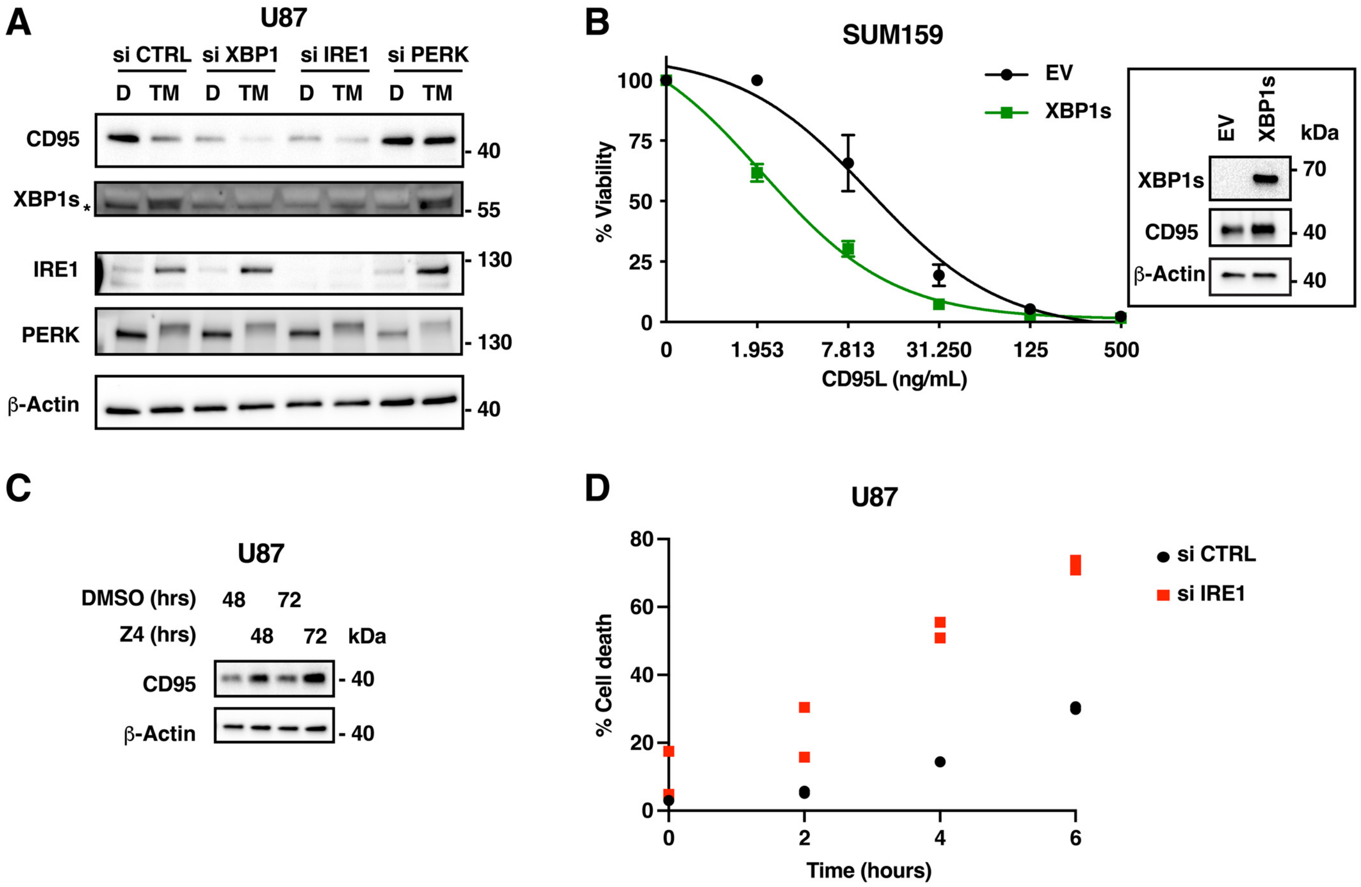

Figure EV4. IRE1 RNase dually controls CD95 expression and CD95L-induced cell death.

(**A**) U87 cells were transfected with siRNA control or targeting XBP1, IRE1 or PERK as indicated. 16 h later, cells were treated with 2.5 μg/mL thapsigargin for 8 h. Lysates were analysed using western blot. One experiment representative of at least three independent ones is shown. (**B**) SUM159 cells were transfected with a plasmid coding for FLAG-XBP1s (XBP1s) or an empty vector (EV). 48 h later, cells were treated with the indicated concentrations of CD95L for 48 h. Viability was assessed using MTT assay and normalized to untreated cell values. Mean ± SEM of three independent experiments. Inset: western blot analyses of cell lysates 48 h post-transfection. (**C**) U87 cells were treated with DMSO or Z4 (25 μM) for the indicated times. Lysates were analysed using western blot. One experiment representative of three independent ones is shown. (**D**) U87 were transfected with siRNA control or targeting IRE1 as indicated. 72 h later, cells were treated with 100 ng/mL CD95L. % of cell death was defined as the % of Cytotox red-positive cells as detected by the Incucyte. Two independent experiments are shown. Source data are available online for this figure.

© The Author(s)

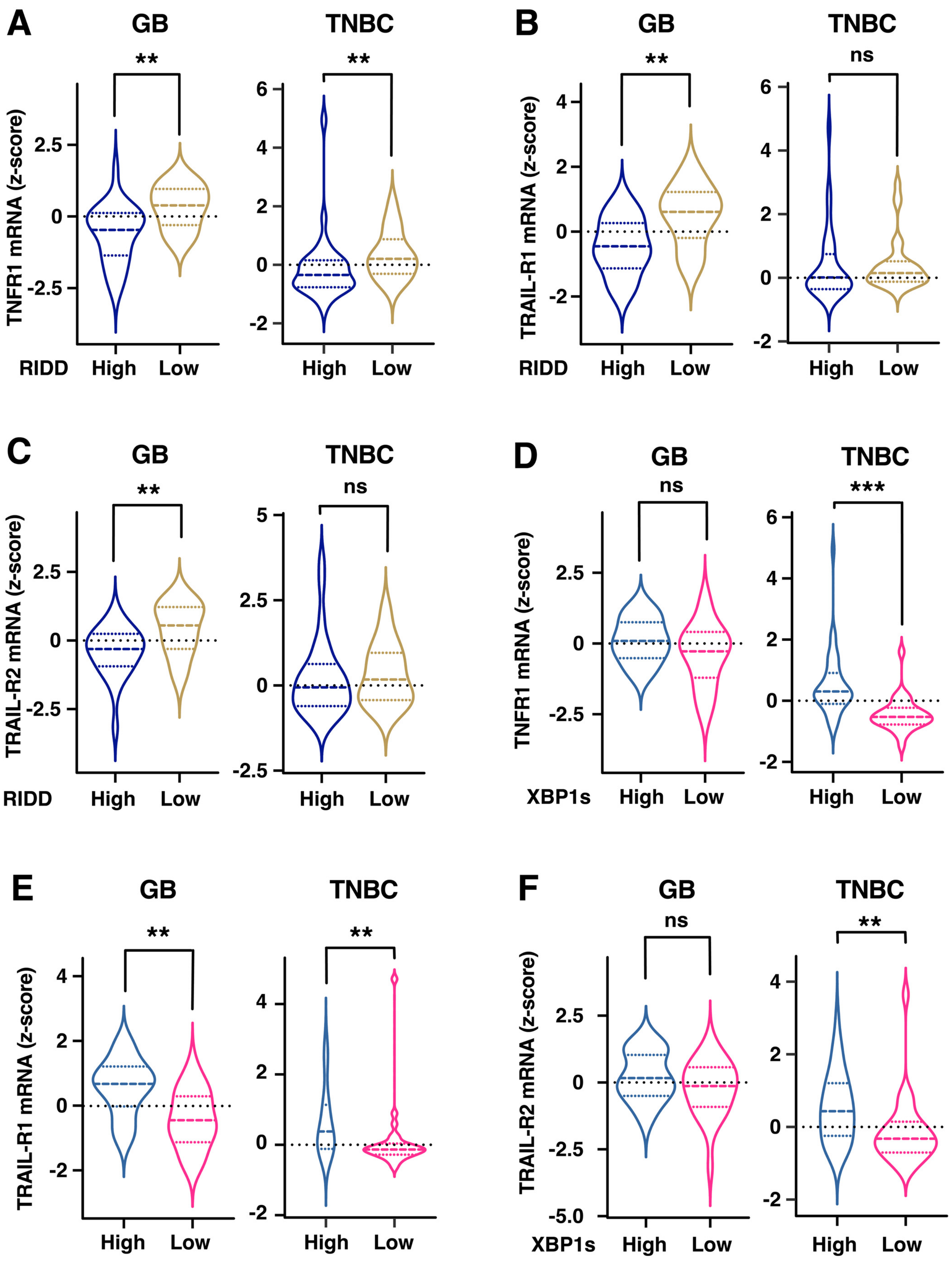
A
GB
TNBC
**
**
TNFR1 mRNA (z-score)
RIDD
High
Low
B
GB
TNBC
**
ns
TRAIL-R1 mRNA (z-score)
RIDD
High
Low
C
GB
TNBC
**
ns
TRAIL-R2 mRNA (z-score)
RIDD
High
Low
D
GB
TNBC
ns
***
TNFR1 mRNA (z-score)
XBP1s
High
Low
E
GB
TNBC
**
**
TRAIL-R1 mRNA (z-score)
High
Low
F
GB
TNBC
ns
**
TRAIL-R2 mRNA (z-score)
XBP1s
High
Low

Figure EV5. Low RIDD activity and high XBP1s activity in tumours correlate with the expression of DR.

(**A–F**) TNFR1 (**A**,**D**), TRAIL-R1 (**B**,**E**), TRAIL-R2 (**C**,**F**) expression z-scores of 45 GB and 62 TNBC tumours were plotted according to the RIDD or XBP1 activity score. The distribution of z-score is represented as violin plots. For GB $n = 45$; for TNBC $n = 62$. Statistical difference of expression between groups was calculated using Mann–Whitney tests and the p-value is indicated ((**A**) $^{**}p = 0.0016$ for GB and $^{**}p = 0.0025$ for TNBC; (**B**) $^{**}p = 0.0017$ for GB and $p = 0.13$ for TNBC; (**C**) $^{**}p = 0.0044$ for GB and $p = 0.34$ for TNBC; (**D**) $p = 0.18$ for GB and $^{***}p = 2e-06$ for TNBC; (**E**) $^{**}p = 0.0011$ for GB and $^{**}p = 0.0017$ for TNBC; (**F**) $p = 0.23$ for GB and $^{**}p = 0.0016$ for TNBC).

 © The Author(s)
